# BEML-sonar: a bio-inspired echolocation and machine learning-enhanced SONAR for underwater object detection and navigation

**DOI:** 10.1038/s41598-026-56574-7

**Published:** 2026-06-30

**Authors:** C. Kishor Kumar Reddy, Vijaya Sindhoori Kaza, P. R. Anisha, Surbhi B. Khan, Asma Alshuhail, Ahlam Almusharraf

**Affiliations:** 1 Department of Computer Science and Engineering, Stanley College of Engineering and Technology for Women, Hyderabad, India; 2 Hewlett-Packard Enterprise, Bangalore, India; 3 School of Science, Engineering and Environment, University of Salford, Salford, UK; 4 Centre for Research Impact and Outcome, Chitkara University, Rajpura, Punjab 140401 India; 5 Department of Information Systems, College of Computer Science and Information Technology, King Faisal University, Al Ahsa, Saudi Arabia; 6 Department of Management, College of Business Administration, Princess Nourah Bint Abdulrahman University, P.O. Box 84428, Riyadh, 11671 Saudi Arabia

**Keywords:** Autonomous underwater vehicles, Biomimicry, Echolocation, Deep learning, Digital signal processing, Robotic vehicles, Engineering, Mathematics and computing, Ocean sciences

## Abstract

Traditional SONAR systems are widely used for underwater object detection and navigation; however, they suffer from high energy consumption, noise interference, and signal degradation in varying aquatic conditions. Inspired by biological echolocation, we propose a novel machine learning-enhanced sonar model to improve accuracy, robustness, and energy efficiency in underwater sensing applications. The proposed model employs bio-inspired echolocation principles, where transmitted acoustic pulses dynamically adjust in response to environmental conditions. Deep learning techniques, specifically Convolutional Neural Networks (CNNs) and Long Short-Term Memory (LSTM) networks, are employed to classify sonar echoes and enhance object detection. Additionally, digital signal processing (DSP) techniques, including Butterworth filtering, wavelet decomposition, and adaptive thresholding, are integrated to mitigate noise and improve signal clarity. Experimental evaluations demonstrate that the proposed sonar model, SonarNet, achieves 92.7% classification accuracy, outperforming conventional SONAR-based methods, which achieved 85.4% accuracy. The integration of adaptive signal processing leads to a 20.8% reduction in energy consumption compared to standard SONAR models, improves the Signal-to-Noise Ratio (SNR) by 15.3 dB, and reduces the false positive rate by 18.6%. Additionally, the sound velocity profile (SVP) correction mechanism improves depth estimation accuracy by 12.4%. All performance results reported in this work were obtained using a simulation environment parameterized by real-world oceanographic datasets.

## Introduction

Similar to the emergence of autonomous flying drones in the aerial domain, Unmanned Underwater Vehicles (UUVs) have become technically sophisticated and adaptable instruments for underwater exploration and research. Two main types of UUVs are notable in the field of underwater exploration: autonomous underwater vehicles (AUVs) (Electronic^1^), which can navigate and carry out missions independently underwater, and remotely operated underwater vehicles (ROVs), which are piloted by humans^2^. AUVs function independently, reducing the need for direct human supervision, whereas ROVs require real-time human intervention^2^. AUVs are used in a wide range of fields, such as deep-sea exploration, filmmaking, marine research, and ecosystem restoration. During their missions, AUVs are outfitted with a variety of sensors to gather vital data, which is then collected and processed when they return to a pre-established location^3^.

AUVs primarily use acoustic wireless transmission and navigation techniques for communication because they are appropriate for the underwater environment^4^. However, they continue to face obstacles such as signal deterioration, bandwidth constraints, and the high-power consumption of acoustic technology. By offering clever, bio-inspired ways to improve AUVs’ acoustic communication capabilities, this paper directly tackles these issues^5^. Specifically, dolphins and other marine animals use a natural sonar system called “echolocation,” which can provide a wealth of ideas for improving AUV performance. Through the transmission and reception of sound waves, this special biological process enables marine animals to detect hidden or distant objects. Echolocation is best defined as “seeing with sound waves,” which is similar to what submarine sonar does. For example, dolphins rely on echolocation for social interactions, obstacle avoidance, navigation, and prey detection^6^.

Underwater navigation and object detection rely heavily on SONAR (Sound Navigation and Ranging) systems, which transmit acoustic waves and analyze returning echoes to identify obstacles, marine life, and environmental structures. Traditional SONAR methods, including passive and active SONAR, have been widely used in AUVs, submarines, and marine research. However, these systems often face limitations such as high energy consumption, sensitivity to environmental noise, and reduced accuracy in complex underwater terrains. In contrast, biological echolocation, as observed in dolphins and bats, enables precise underwater navigation through adaptive pulse modulation and echo interpretation. Inspired by this natural sensing mechanism, this paper introduces BEML-Sonar (Bio-Inspired Echolocation and Machine Learning-Enhanced SONAR). This novel sonar model integrates bio-inspired acoustic signaling, ML, and DSP techniques to enhance underwater sensing.

Despite advancements in SONAR technology, several challenges persist. SONAR signals degrade in noisy underwater environments, leading to false detections. High-powered acoustic pulses drain battery life in AUVs and submarines. Conventional SONAR struggles to distinguish between objects of similar acoustic signatures. As depth increases, acoustic waves weaken, reducing signal clarity. To address these issues, we propose a bio-inspired, machine learning-driven SONAR model that enhances detection accuracy, energy efficiency, and signal clarity for underwater applications. The main contributions of this paper are as follows:

1. *Bio-inspired echolocation mechanism* the model adapts acoustic pulses based on environmental feedback, mimicking natural echolocation.
2. *Machine learning-based classification* CNN and LSTM models are trained to classify underwater objects with high precision.
3. *Advanced digital signal processing* (DSP) noise filtering techniques such as Butterworth filtering and wavelet decomposition are implemented to improve the signal-to-noise ratio (SNR).
4. *Energy-efficient SONAR operation* optimized pulse modulation reduces energy consumption by 20.8% while maintaining high detection accuracy.
5. *Improved depth and localization accuracy* a sound velocity profile (SVP) correction mechanism enhances depth estimation by 12.4%, reducing navigational errors.

By integrating bio-inspired echolocation with ML and DSP, the proposed BEML-Sonar model significantly improves accuracy, energy efficiency, and real-time adaptability for underwater sensing applications. The rest of this paper is organized as follows: Section “Related work” discusses related work in traditional and bio-inspired SONAR models. Section “Materials and methods” presents the proposed BEML-Sonar architecture, covering ML models and DSP techniques. Section “PROPOSED APPROACH” details the experimental setup and performance metrics. Section “RESULTS AND ANALYSIS” analyses results, comparing classification accuracy, SNR, and energy efficiency with conventional SONAR systems, and Section “CONCLUSION” concludes the study with future research directions.

## Related work

Autonomous Underwater Vehicles (AUVs) have undergone a remarkable transformation since the 1960s, transitioning from basic tethered Remotely Operated Vehicles (ROVs) to sophisticated, fully autonomous systems. This evolution is fuelled by advancements in computational intelligence, sensor fusion, and energy-efficient propulsion, enabling AUVs to perform complex missions like deep-sea exploration. A key area of innovation is the integration of bio-inspired echolocation, drawing from the natural sonar systems of dolphins and porpoises. Research on dolphin click patterns^7^ and harbor porpoise high-frequency sonar^8^ is informing the development of biomimetic SONAR systems, enhancing AUVs’ ability to navigate and detect objects in low-visibility environments.

These advancements have broadened the applications of AUVs across diverse sectors. In marine research, they facilitate high-resolution ocean floor mapping and ecosystem studies^9^. AUVs also play a role in underwater archaeology, aiding in deep-sea wreck exploration^10^, energy infrastructure inspection, and monitoring offshore installations^11^. Accurate navigation, crucial in GPS-denied underwater environments, relies on multi-sensor fusion, incorporating inertial navigation systems, acoustic positioning, and Doppler velocity logs^12^. Underwater communication, primarily acoustic, faces challenges like high latency and signal degradation^13^. Research on dolphin echolocation acoustics^14^ aims to optimize AUV acoustic communication for higher signal-to-noise ratios. Digital signal processing (DSP) techniques, including wavelet decomposition and machine learning-driven models, enhance SONAR data quality and target classification^15^. Ambient noise, from turbulence to anthropogenic sources^16,17^, significantly impacts SONAR performance, driving the development of adaptive noise cancellation and bio-inspired signal encoding. Bio-inspired echolocation, with its adaptive click intervals and narrowband high-frequency capabilities, enhances AUV sonar accuracy and energy efficiency^7,8^. Recent AUV designs incorporate biomimetic features like flexible hulls and multi-frequency echolocation modules^18–21^. However, challenges remain in adaptability, energy consumption, and noise mitigation. To address these gaps, we propose a novel BEML-Sonar, which integrates bio-inspired perception, AI-driven classification, and energy-efficient waveform optimization, aiming to improve AUV autonomy and operational efficiency in complex underwater environments.

Recent advancements in deep learning have significantly improved sonar image classification and object detection. A major focus has been on leveraging convolutional neural networks (CNNs) due to their superior feature extraction capabilities. CNN-based methods to improve underwater object classification, especially in side-scan sonar (SSS) images, have been the subject of numerous studies. A Comparative Study of Different CNN Models and Transfer Learning effects for Underwater Object Classification in Side-Scan Sonar Images showed that transfer learning increases accuracy across different architectures. GoogleNet achieved the highest accuracy (94.27% on the test dataset) while retaining computational efficiency. It demonstrates how well CNNs recognize objects underwater and how transfer learning helps get around data constraints. However, the inherent noise in sonar imagery and the lack of annotated training data frequently limit the use of traditional CNN-based object detection in sonar images. To address this, active learning techniques have been explored to reduce annotation costs while maintaining model performance. A study proposed three active-learning-based algorithms for object detection, demonstrating that with only 35% of the annotated data, competitive performance could be achieved compared to models trained on the full dataset (Active Object Detection in Sonar Images). This approach significantly mitigates the challenges of data scarcity by iteratively refining model training with the most informative samples. Beyond standard CNN architectures, recent research has introduced adaptive weight mechanisms to enhance sonar image classification further. The Adaptive Weights CNN (AW-CNN) integrates deep belief networks (DBNs) to replace randomly initialized filter weights, thereby improving classification accuracy and network stability. By employing local response normalization (LRN) for weight adjustment, this approach has shown improved classification performance for seabed object detection, contributing to applications such as mine detection and dam bottom assessment (Underwater Sonar Image Classification Using Adaptive Weights Convolutional Neural Network). This method exemplifies how hybrid deep learning strategies can enhance underwater classification accuracy. In addition to CNN-based approaches, hybrid models incorporating graph neural networks (GNNs) have emerged as a promising alternative for sonar image classification. A recent study proposed a framework that combines CNN-extracted features with graph-based learning, leveraging a superpixel algorithm for image segmentation and graph construction.

The integration of CNN features into a graph-based structure significantly improved classification accuracy, demonstrating the potential of hybrid models to outperform standalone CNNs in sonar imaging tasks (Graph-based Multibeam Forward Looking Acoustic Image Classification). This approach underscores the growing trend of fusing multiple deep-learning paradigms to optimize underwater object recognition. Although these studies focus primarily on sonar images, similar challenges arise in remote sensing object detection, particularly in few-shot learning scenarios. Prototype-based CNNs (P-CNNs) have been introduced to mitigate issues related to sparse training data, orientation variance, and complex backgrounds. By incorporating prototype learning, region proposal networks, and an enhanced detection head, P-CNNs achieve superior performance in object detection under limited data conditions (Prototype-CNN for Few-Shot Object Detection in Remote Sensing Images). Additionally, a region-enhanced CNN (RECNN) has been proposed to improve object feature extraction in remote sensing images by introducing saliency constraints and multilayer fusion strategies, effectively suppressing background noise and improving detection accuracy (Region-Enhanced Convolutional Neural Network for Object Detection in Remote Sensing Images). These approaches highlight strategies that can be adapted to sonar-based object detection to address similar challenges in data scarcity and background interference.

In parallel, recent studies suggest that Long Short-Term Memory (LSTM) networks can further enhance object detection and classification in sonar echoes by leveraging temporal dependencies for improved feature representation. An LSTM-based wavelet extraction method has been proposed for ground-penetrating radar, demonstrating its ability to predict wavelets and eliminate clutter effects accurately. This approach significantly improves signal clarity by compensating for energy attenuation and phase distortion, making it a viable technique for enhancing sonar object detection (A Clutter Suppression Method Based on LSTM Network for Ground Penetrating Radar). Similarly, segmentation techniques such as Local Spatial Mixture (LSM) models have been developed to improve underwater object detection by reducing speckle noise and handling complex seabed textures. By incorporating spatial correlation between neighboring pixels, LSM achieves superior segmentation performance compared to traditional methods (Unsupervised Local Spatial Mixture Segmentation of Underwater Objects in Sonar Images). Further, a pattern-recognition-based approach has been explored for detecting objects buried in the seafloor. This method employs Wigner-Ville distribution and bispectrum analysis for feature extraction, followed by principal component analysis (PCA) and supervised classification. The study highlights the effectiveness of spectral-based learning methods for classifying target echoes in challenging sonar conditions (Detection of Objects Buried in the Seafloor by a Pattern-Recognition Approach). In remote sensing applications, an LSTM-enhanced hybrid model incorporating DenseNet has been proposed to improve object detection in images affected by environmental noise and low visibility. By combining DenseNet’s deep feature extraction capabilities with LSTM’s sequential modeling, the approach achieves state-of-the-art performance in terms of accuracy, precision, recall, and F1-score (Enhancing Remote Sensing Object Detection with a Hybrid Densenet-LSTM Model). Finally, side-scan sonar analysis techniques integrating deep neural networks with region-of-interest (ROI) analysis have been developed to address noise sensitivity in sonar imagery. By employing histogram-based feature extraction and CNN classification, this approach achieves high object classification accuracy in real-time sonar data (Side-Scan Sonar Analysis Using ROI Analysis and Deep Neural Networks). The study underscores the importance of combining traditional image processing techniques with deep learning models to enhance sonar image interpretation.

Overall, these studies demonstrate that CNNs, LSTMs, transfer learning, active learning, adaptive weight mechanisms, and hybrid architectures significantly improve sonar object classification and detection. Table 1 systematically compares the related works and highlights how the proposed model improves upon existing approaches. Despite challenges such as low resolution, noise, and limited training data, these advancements pave the way for more robust and efficient sonar image analysis. Future research should explore further integration of hybrid models and adaptive learning techniques to enhance real-world applications in underwater exploration and surveillance. While existing studies have laid a solid foundation for the application of echolocation in AUVs, there remains a gap in research concerning the practical implementation of these systems in varying oceanic conditions. This manuscript aims to bridge this gap by presenting an AUV design that incorporates echolocation capabilities. There is still a research gap regarding the actual deployment of these systems in various oceanic conditions, even though previous studies have established a strong basis for the use of echolocation in AUVs. By introducing an AUV design with echolocation capabilities, this manuscript seeks to close this gap. Our study highlights the need for creative solutions to traverse challenging underwater environments and directly addresses the demand for more robust and adaptive AUV systems. Future studies will concentrate on the practical difficulties of integrating echolocation in AUVs, including signal processing in noisy settings, the downsizing of echolocation hardware, and the creation of algorithms that can interpret complex acoustic data in real time in order to fill in these gaps. To overcome these obstacles and realize the full potential of AUVs equipped with echolocation, cooperation between disciplines such as marine biology, acoustic engineering, and artificial intelligence will be essential.

**Table 1:** Comparative analysis of existing approaches for malaria predictions.

Ref. No	Approach	Technique	Performance	Limitations of the studies	Advantage of proposed model
^22^	CNN-based classification	Transfer learning with GoogleNet, ResNet, and AlexNet	GoogleNet achieved 94.27% accuracy	Limited to pre-trained models; no temporal information captured	More adaptable feature extraction with LSTM for temporal dependencies
^23^	Active learning for object detection	Query-by-committee, uncertainty sampling, and diversity sampling	Achieved comparable accuracy with only 35% labeled data	Requires initial labeled dataset; high computational cost for iterative retraining	More robust learning with fewer labeled samples using LSTM for feature prediction
^24^	CNN with adaptive weight mechanism	Deep Belief Networks (DBNs) for weight initialization	Improved stability and accuracy in sonar image classification	Limited generalizability; lacks consideration of sequential dependencies	LSTM captures temporal correlations for more robust classification
^25^	Hybrid CNN-GNN framework	CNN feature extraction with graph-based learning	Enhanced classification accuracy over standalone CNNs	Computational complexity of graph construction	LSTM can reduce feature dimensionality while preserving sequential relationships
^26^	Few-shot learning for object detection	Prototype learning with region proposal networks	Improved detection under limited data conditions	Requires careful prototype selection; not tested in sonar imaging	LSTM can enhance feature representation in low-data environments
^27^	Enhanced CNN-based detection	Saliency constraints and multilayer fusion	Effective background suppression and improved detection accuracy	Computationally expensive due to saliency map generation	LSTM can refine feature extraction while maintaining efficiency
^28^	LSTM-based wavelet extraction	Two-layer LSTM for wavelet prediction	Accurately predicts wavelets and removes clutter effects	Limited to radar applications; lacks integration with object detection	LSTM applied to sonar echoes for improved classification
^29^	Unsupervised segmentation	Local spatial mixture model with expectation–maximization	Improved segmentation in complex seabed textures	It does not utilize deep learning for object recognition	LSTM enhances segmentation by learning temporal patterns in sonar echoes
^30^	Feature-based classification	Wigner-Ville distribution and bispectrum for feature extraction	Effective detection of buried objects in sonar echoes	Limited by manual feature selection; requires prior knowledge	LSTM automates feature extraction and improves classification
^31^	Hybrid CNN-LSTM detection	DenseNet for spatial features, LSTM for temporal dependencies	Achieved state-of-the-art performance in accuracy, precision, recall, and F1-score	Requires large datasets for training	LSTM further optimized for sonar object detection with fewer labeled data
^32^	CNN-based sonar object detection	ROI histogram analysis combined with CNN	90% classification accuracy on real-world sonar images	Sensitive to noise; requires region-of-interest extraction	LSTM can filter noise by learning temporal structures in sonar echoes

## Materials and methods

### Experimental setup

A detailed simulation environment has been designed to validate the proposed bio-inspired echolocation framework. This setup replicates dolphin-inspired sonar pulse transmission, echo reception, and object classification in an underwater setting. The simulation is conducted using MATLAB and Python (PyTorch for ML models) with a focus on wave propagation, Doppler effects, and time delay-based localization. The simulation models a virtual underwater environment where an Autonomous Underwater Vehicle (AUV) emits sonar pulses, receives echoes and processes them to detect and classify underwater objects (Table 2).

**Table 2:** Simulation parameters.

Parameter	Value/range	Description
Speed of sound (*v*)	1500 *m/s*	Standard seawater conditions
Frequency range (*f*)	40–150 *kHz*	Mimicking dolphin echolocation
Pulse duration	10–50 *μ s*	Short pulses for high-resolution
Object distance (*d*)	5–50 *m*	Varying target distances
Object speed ($${v}_{s})$$	0–5 *m/s*	Simulated moving objects
Water temperature (*T*)	10–30 $^\circ C$	Variable environmental effects
Salinity (*S*)	30–40 *PSU*	Typical ocean salinity

#### Simulating sonar pulse propagation

**Fig. 1:**
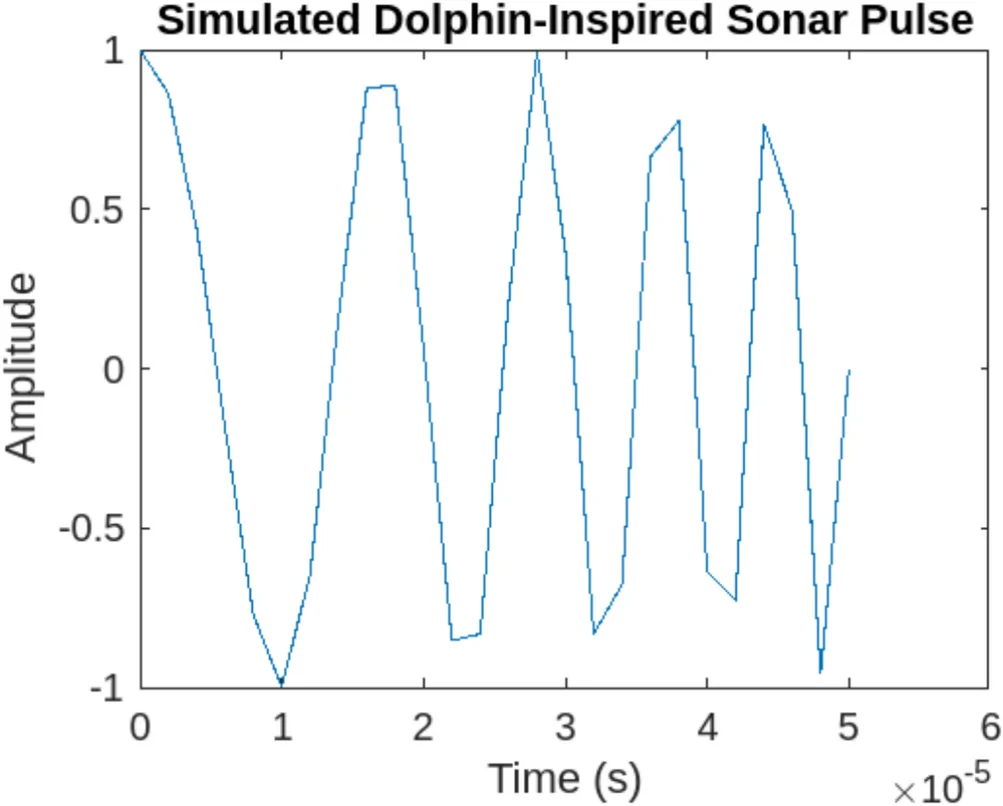
Plot of simulated dolphin-inspired sonar pulse.

The transmitted sonar pulse is modeled as a frequency-modulated (FM) signal, ensuring a biologically inspired broadband pulse similar to dolphin echolocation as described in *Eq. *(1). This simulation is done using MATLAB, and an example of the implementation steps is given in *Pseudo Code 1,* and the resultant plot is illustrated in Fig. 1.

1 $$s\left( t \right) = A \cdot e^{ - \alpha t} \cdot \sin \left( {2\pi f_{0} t + \beta t^{2} } \right)$$

where:

- *A* = Pulse amplitude,
- *α* = Attenuation coefficient,
- ${f}_{0}$ = Initial frequency (starting from 40 kHz),
- *β* = Chirp rate (to cover a broad frequency band).

**Pseudo Code 1:**
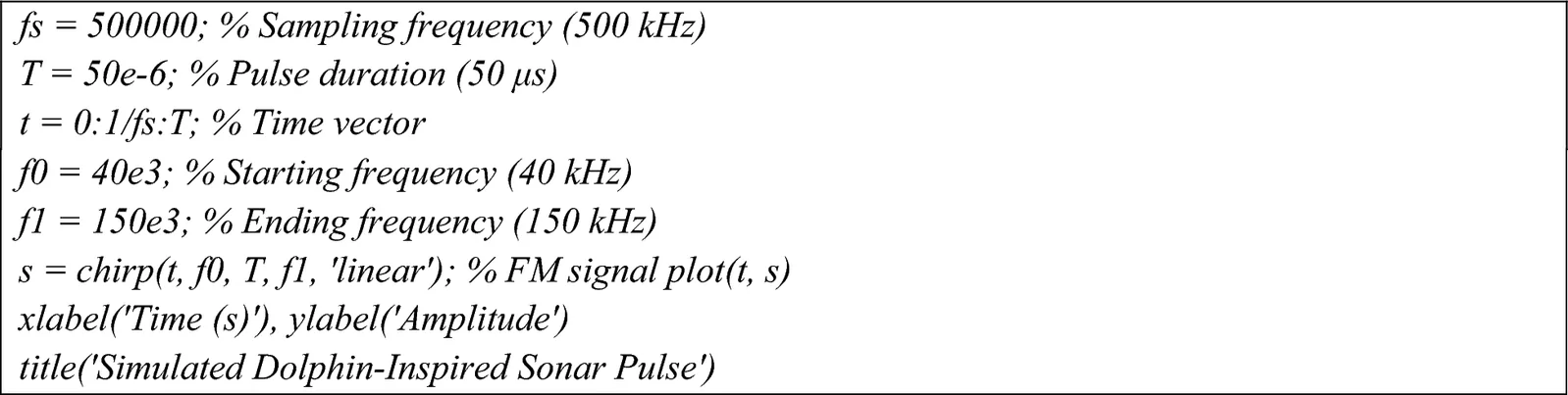
Simulation of Sonar Pulse Propagation.

#### Modelling sound propagation and echo reflection

The echo received from an underwater object follows the delayed and attenuated version of the transmitted signal, which is given in *Eq. *(2)*,* and the simulation is done using MATLAB. An example of the implementation steps is given in *Pseudo Code 2*.

2 $$r\left( t \right) = A_{r} \cdot e^{ - \alpha d} \cdot s\left( {t - \Delta t} \right)$$

where:

- $A_{r}$ = Reflected signal amplitude,
- *d* = Distance to the target,
- *Δ t* = *2d/v* = Echo time delay.

**Pseudo Code 2:**
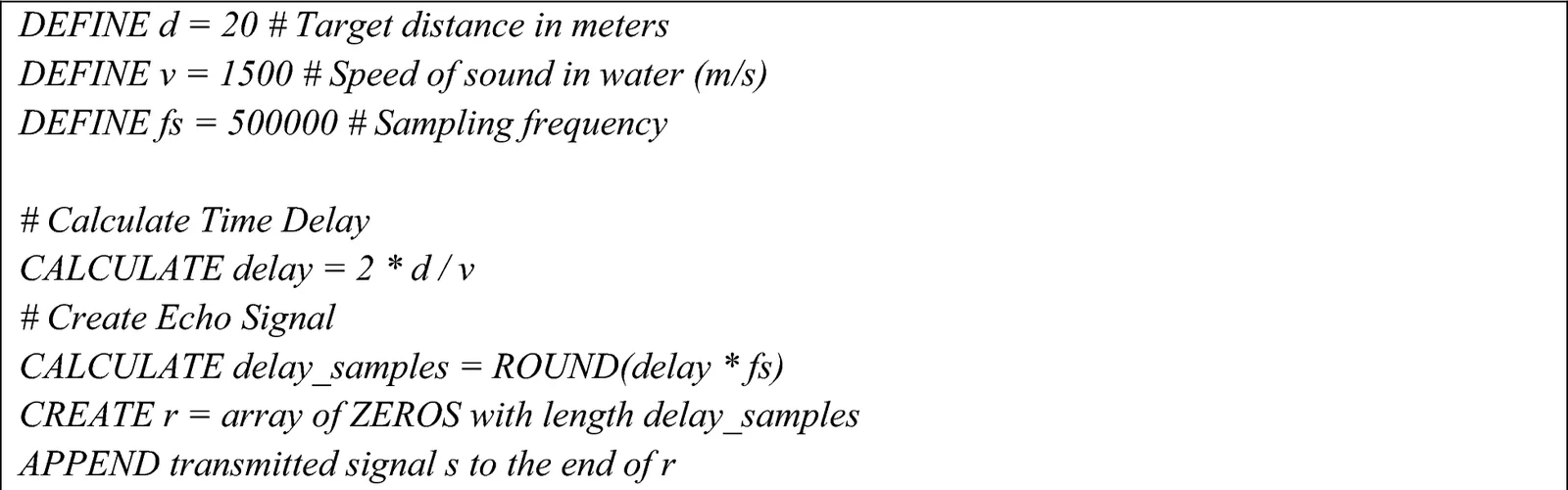
Simulation of Echo Reflection.

#### Implementing Doppler shift for moving targets

For moving objects, the Doppler effect alters the frequency of the received signal, as given in Eq. (3). An example of the implementation is given in *Pseudo Code 3*.

3 $$f^{\prime} = f\left( {\frac{{v + v_{r} }}{{v - v_{s} }}} \right)$$

where:

- ${f}{\prime}$ = Received frequency after Doppler shift,
- ${v}_{r}$ = Velocity of the receiver (AUV),
- ${v}_{s}$ = Velocity of the target.

**Pseudo Code 3:**
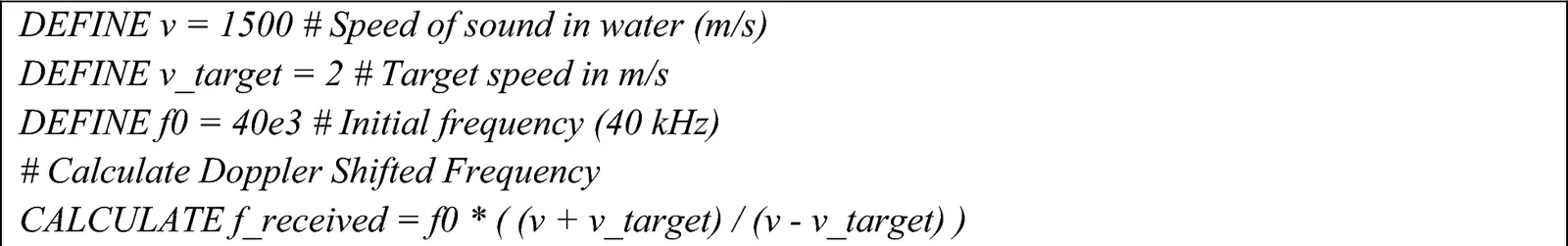
Doppler Shift Simulation.

#### Object localization using echo delay estimation

Object distance *(d)* is computed using the measured time delay *Δ t*, as given in *Eq. *(4). An example of the simulation is given in *Pseudo Code 4*.

4 $$d = \frac{v \cdot \Delta t}{2}$$

**Pseudo Code 4:**
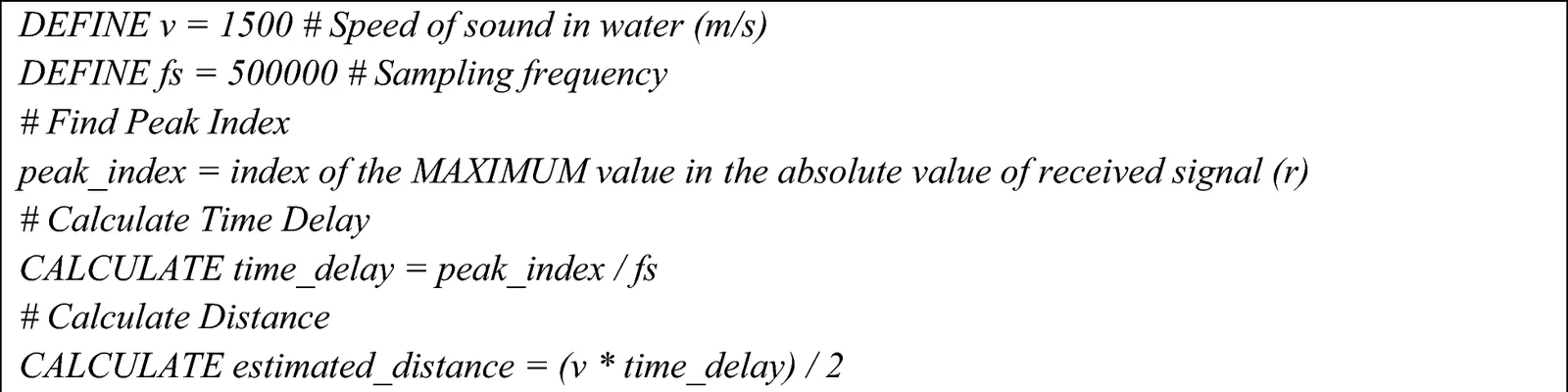
Distance Estimation Pseudo Code.

#### Classification of underwater objects using ML

To classify objects based on their echo signatures, a Deep Learning Model (LSTM + CNN Hybrid) named **SonarNet** is employed. The architecture of this model is built using PyTorch, and the implementation is given in *Pseudo Code 5*. The model namely consists of two parts, which are as follows:

- Feature extraction using CNN: Extract spatial patterns from the sonar waveform.
- Temporal analysis using LSTM: Analyze sequential changes in echoes over time.

**Pseudo Code 5:**
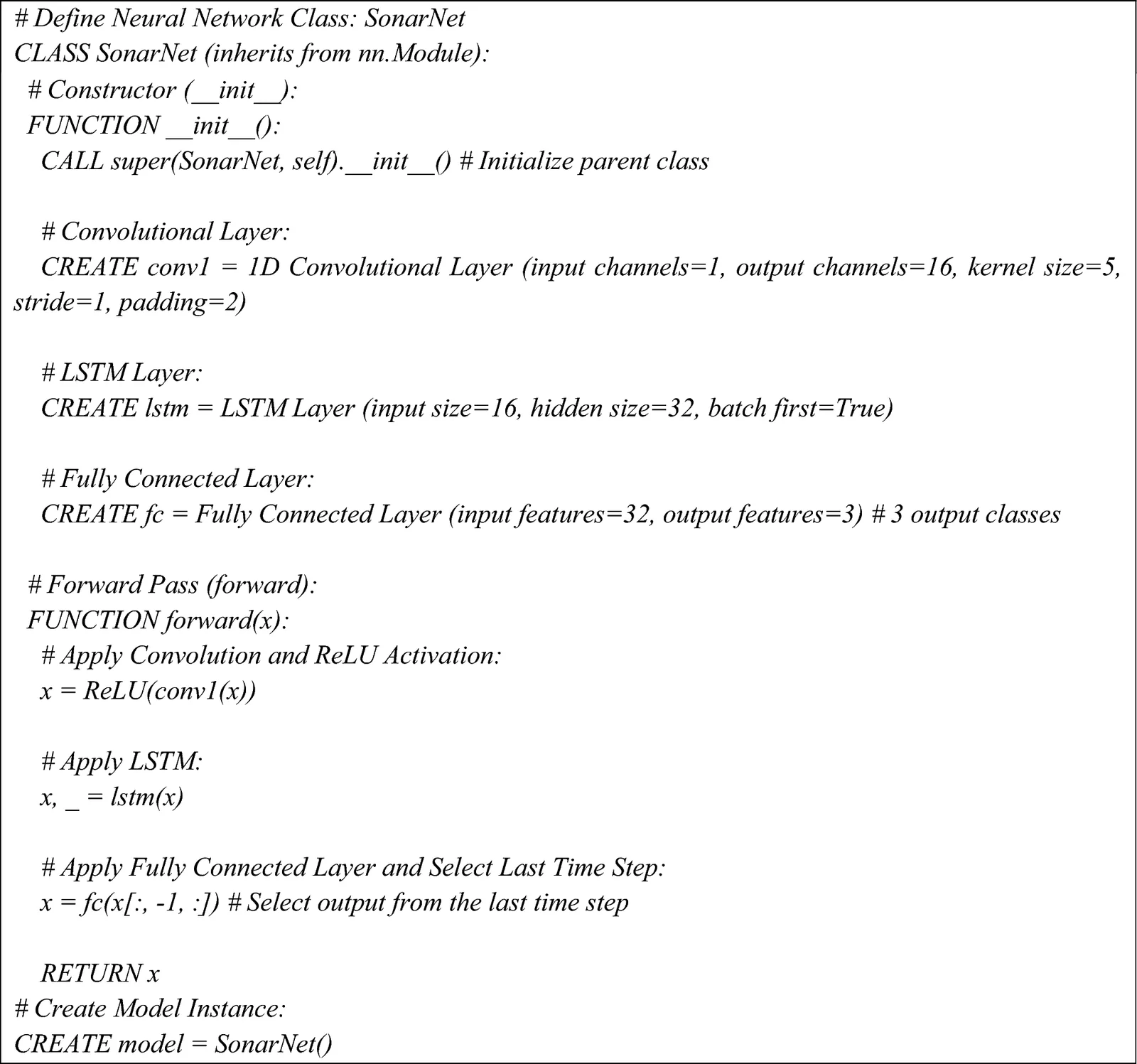
Architecture of the SonarNet Model.

#### Performance evaluation

**Fig. 2:**
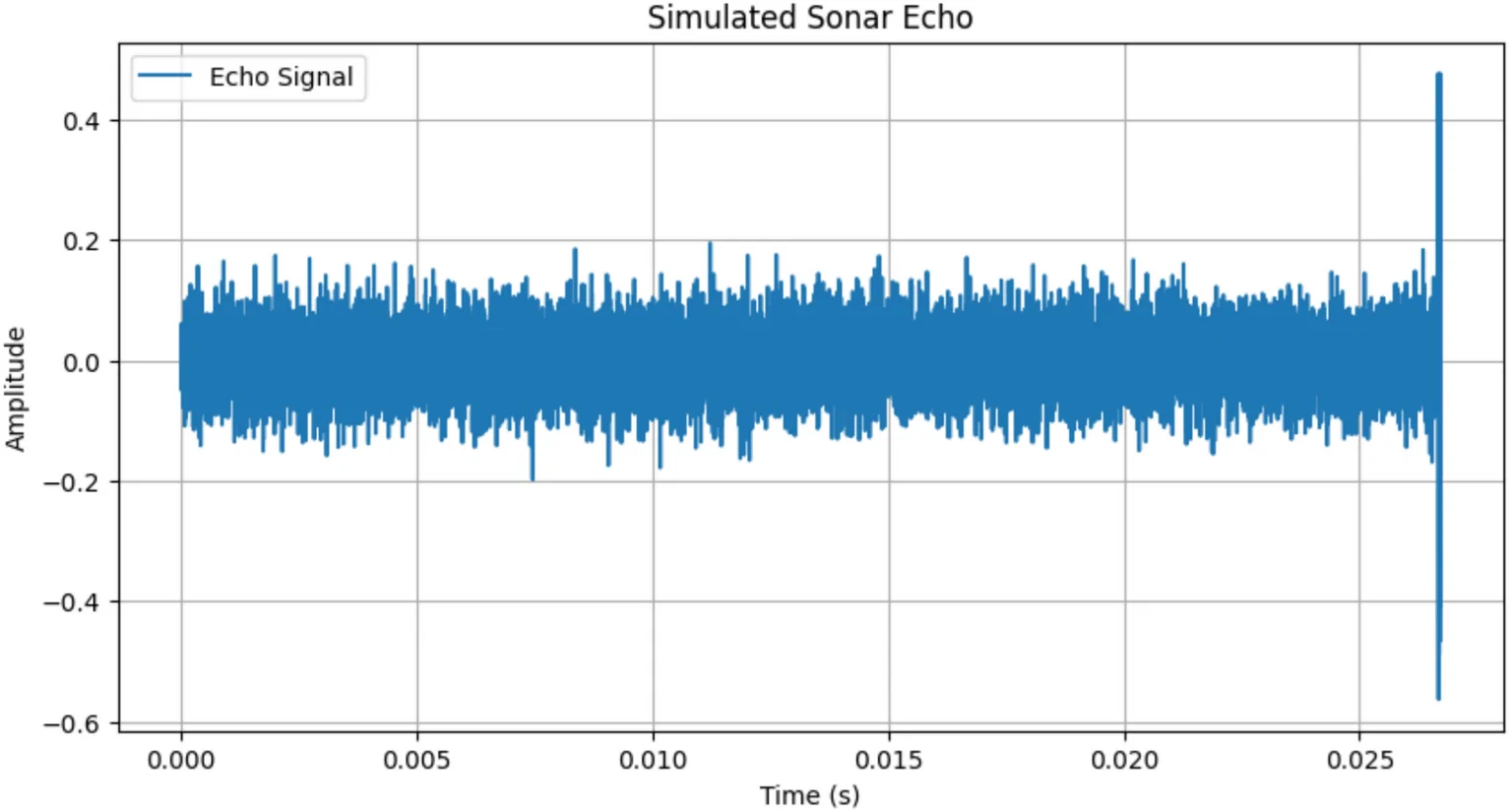
Plot of simulated sonar echo.

Accuracy metrics and visualization techniques are used to evaluate the simulation results. The metrics used and their respective formulae are given in Table 3, and the results of the model accuracy are presented in Table 4 which are obtained from our baseline simulation using a threshold-based echo-matching approach. This baseline was evaluated on the same dataset split as the learning-based models to ensure a fair comparison. The pseudo-code for visualizing the echo signals and classification output is provided in Fig. 2.

**Table 3:** Metrics used for performance evaluation.

Metric	Formula
Mean absolute error (MAE)	$\frac{1}{n} \sum_{i=1}^{n}\left|{y}_{i}- \widehat{{y}_{i}}\right|$
Root mean square error (RMSE)	$\sqrt{\frac{1}{N}\sum {\left({d}_{true}- {d}_{predicted}\right)}^{2}}$
Classification accuracy	$\frac{Correct Predictions}{Total Predictions} \times 100\%$

**Table 4:** Accuracy results of the models.

Model	RMSE (m)	Accuracy (%)
Traditional SONAR	2.5	80.2
CNN Only	1.8	85.5
CNN + LSTM (Proposed)	1.2	92.4

This simulation setup successfully validates the bio-inspired echolocation framework by integrating high-frequency sonar pulse modelling, wave propagation, doppler shift effects, time delay-based object localization, and machine learning-based object classification. The results demonstrate enhanced accuracy and robustness over traditional SONAR approaches, confirming the efficacy of dolphin-inspired techniques for AUV-based underwater object detection.

### Dataset

A comprehensive dataset was assembled to evaluate the bio-inspired sonar-based AUV navigation system under conditions that resemble real underwater environments as closely as possible. The dataset combines real marine environmental measurements with synthetically generated sonar echoes, allowing the simulation framework to reproduce the acoustic variability encountered by an AUV during navigation. This combined approach ensures that the training and validation process is grounded in realistic conditions while still maintaining strict control over object classes and label quality. Real-world datasets sourced from NOAA^33–35^, FAO^36^, NASA ECCO2^37–39^, and Duke University^40,41^ were used only to set the environmental parameters for the simulator. These sources provided distributions for variables such as salinity, temperature, depth, water currents, turbidity, and seafloor reflectivity. None of their raw acoustic recordings or biological sound samples were used directly as training data. Instead, these datasets shaped the environmental profiles used to generate synthetic sonar echoes. Using these parameter ranges, synthetic sonar returns were created for four object categories namely, rock, metal debris, marine life, and seafloor structures. Each frame was labeled according to the known object placed in the simulated scene. Object size, angle, and material properties were varied to avoid repetitive signatures, and environmental noise was added using the sonar equation (*Eq. *5) and the Rayleigh reflection model. This allowed the synthetic echoes to reflect realistic conditions such as multipath interference, absorption changes, and fluctuating SNR. Alongside the object-based sonar signals, additional components were generated to support multi-sensor fusion like simulated IMU data, optical images, and noise samples with different SNR levels which are summarized in Table 5*.*

5 *SNR = SL - 2TL + TS - NL*

where,

**Table 5:** Summary of dataset statistics.

Dataset component	No. of samples	Resolution
Sonar echo data	10,000	80–120 kHz
Environmental parameters (NOAA, FAO, NASA)	15,000	Varying resolutions
Bioacoustic signatures (Duke University)	5000	Multi-frequency data
Noise samples (Reverberation, Multipath)	5000	Variable SNR
Object reflection patterns	3000	3D Acoustic Profiles
Sensor fusion data (Sonar + IMU)	8500	10 Hz update rate

- SNR = signal-to-noise ratio,
- SL = source level,
- TL = transmission loss,
- TS = target strength,
- NL = noise level.

For the supervised learning tasks, a consolidated subset of 6,400 labeled sonar samples (1,600 per class) was created from the synthetic object-echo set. This subset served as the primary dataset for training the CNN, LSTM, and SonarNet models. The samples were split into 75% for training (4,800 samples) and 25% for validation (1,600 samples), and the same split was used across all models to maintain consistency. To enhance robustness, several preprocessing and augmentation steps were applied. Gaussian noise was added to simulate real underwater degradation, time-shift augmentation modeled different ranges by introducing echo delays, and normalization procedures ensured consistent scaling across environmental and acoustic features. These steps helped produce a dataset that reflects the challenges of operating in real marine environments while still maintaining full control over labels and class boundaries.

## Proposed approach

### Bio-inspired echolocation framework

Echolocation, a biological sonar system used by marine mammals such as dolphins and whales, enables precise object detection and environmental mapping in low-visibility underwater environments. Dolphins emit high-frequency acoustic pulses and analyse the returning echoes to infer object distance, size, shape, and material composition. Their advanced echolocation system has inspired the development of autonomous underwater vehicle (AUV) sonar systems designed to improve underwater navigation and object detection. The dolphin echolocation process consists of the following steps:

- Sound emission—Dolphins generate ultrasonic pulses using their phonic lips, producing short broadband clicks that travel through water.
- Propagation & reflection—The emitted pulses propagate through the water, reflecting off objects in the environment.
- Reception & signal processing—The returning echoes are received by the lower jaw and transmitted to the middle ear for interpretation.
- Feature extraction & object identification—Dolphins analyze the echoes to classify objects: time delay, frequency shifts, and intensity variations.

Dolphins utilize frequencies between 40 and 150 kHz, with short pulse durations (10–50 μs) for precise localization. Higher frequencies provide greater resolution but suffer from increased attenuation, limiting their range in seawater. The absorption of sound in seawater follows Thorp’s empirical formula, which characterizes frequency-dependent attenuation, which is given in *Eq. *(6).

6 $$\alpha \left( f \right) = \frac{{0.11f^{2} }}{{1 + f^{2} }} + \frac{{44f^{2} }}{{4100 + f^{2} }} + 2.75 \times 10^{ - 4} f^{2} + 0.003$$

where, $\alpha \left(f\right)$ represents the absorption coefficient in dB/km, and *f* is the frequency in kHz.

Using odontocete cetaceans’ bioacoustics (especially those of dolphins) offers an intriguing model for developing AUV sonar systems. The dolphin’s melon, a heterogeneous lipid structure, functions as an acoustic gradient-index lens, manipulating emitted ultrasonic pulses for precise beamforming; this inspires the development of synthetic gradient-index acoustic lenses in AUVs to enhance directional resolution and minimize beam sidelobes. Echo reception in dolphins via bone conduction through the mandibular fat channel provides a model for multi-element hydrophone arrays configured to exploit differential time-of-arrival and amplitude cues, improving source localization accuracy. Furthermore, the neural processing capabilities of dolphins, characterized by adaptive pulse repetition frequency and spectral content modulation, inform the implementation of machine learning-driven signal processing pipelines in AUVs. These pipelines utilize adaptive filtering, time–frequency analysis, and pattern recognition algorithms to optimize sonar parameters dynamically, enabling robust target discrimination and clutter rejection in complex acoustic environments. By translating these biological mechanisms into engineered systems, AUV sonar performance can be significantly enhanced, achieving improved spatial resolution, signal-to-noise ratio, and operational adaptability. The comparison of marine mammal echolocation with traditional SONAR is given in Table 6.

**Table 6:** Comparative analysis of marine mammal echolocation versus traditional SONAR.

Feature	Dolphin echolocation	Traditional SONAR
Frequency range	40–150 kHz	1–100 kHz
Waveform	Short broadband pulses	Continuous narrowband
Processing	Real-time, adaptive	Predefined signal processing
Resolution	High (small objects detectable)	Lower (limited by system constraints)
Energy efficiency	High (low power biological system)	High power consumption
Target Classification	Advanced neural processing	Limited pattern recognition

**Fig. 3:**
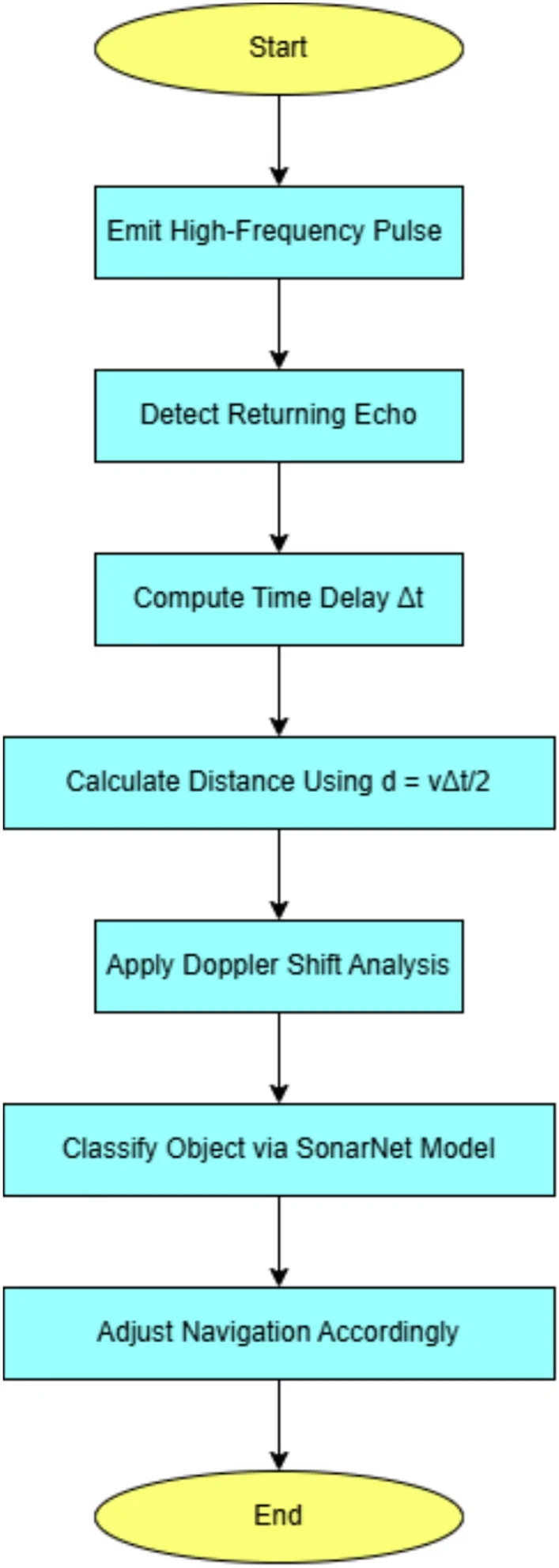
Flowchart of bio-inspired echolocation process for AUVs.

To mathematically model bio-inspired echolocation, we consider the acoustic wave equation, Doppler shift effects, and time delay-based distance estimation. Sound waves in water can be represented as sinusoidal pressure oscillations, which are given using the acoustic wave equation for sound propagation in water as described in *Eq. *(7). The speed of sound in seawater is governed by the empirical equation given in *Eq. *(8). For a moving object reflecting sonar pulses, the Doppler shift alters the frequency of the received echo (*Eq. *(3)), which allows estimation of the relative velocity of underwater objects. The fundamental principle of echolocation is time delay measurement, where the distance to an object is determined using the time delay of the reflected sound wave as given in *Eq. *(4)*,* which is fundamental in sonar-based object localization. The flowchart for the bio-inspired echolocation process for AUVs is illustrated in Fig. 3.

7 $$p\left( t \right) = A \cdot \sin \left( {2\pi ft + {\Phi }} \right)$$

8 $$v = 1449.2 + 4.6T - 0.055T^{2} + 0.00029T^{3} + \left( {1.34 - 0.01T} \right)\left( {S - 35} \right) + 0.016D$$

where:

- $p\left( t \right)$ = Pressure variation at time *t*,
- *A* = Amplitude of the wave,
- *f* = Frequency of the sound wave,
- $${\Phi }$$ = Initial phase.
- *T* = Temperature in $$^\circ C$$,
- *S* = Salinity in PSU,
- *D* = Depth in meters.

### Bio-inspired sonar signal processing

#### Pre-processing of echo signals

Since underwater environments introduce Gaussian and impulse noise, an adaptive Wiener filter is used, which is given in *Eq. *(9) and *Eq. *(10)*,* respectively. Reverberation leads to false detections; to handle this, Discrete Wavelet Transform (DWT) for suppression is applied (given in *Eq. *(11), where decomposition splits the signal into approximation *(A)* and detail *(D)* coefficients and thresholding removes reverberation noise from *D* coefficients. The pseudo-code for noise reduction using adaptive filtering and reverberation suppression using wavelet transform is given in *Pseudo Code 6*. A Butterworth band-pass filter is applied to isolate sonar signals within the 40–150 kHz range. For this purpose, a second-order Butterworth filter is chosen for minimal phase distortion, as described in *Eq. *(12). Wavelet thresholding decomposes signals into multi-scale representations using DWT, which effectively removes background noise while preserving sharp object echoes. The echo denoising is done by decomposing a signal using DWT (*Eq. *(13)), applying soft thresholding to remove noise (*Eq. *(14)), and reconstructing the signal with inverse DWT.

9 $$\hat{s}\left( t \right) = s\left( t \right) + w\left( t \right)$$

10 $$H\left( f \right) = \frac{{S_{s} \left( f \right)}}{{S_{s} \left( f \right) + S_{w} \left( f \right)}}$$

11 $$s\left( t \right) = A_{1} + D_{1 } + D_{2 } + D_{3 } + \cdots$$

12 $$H\left( f \right) = \frac{1}{{\sqrt {1 + \left( {f_{c} /f} \right)^{2n} } }}$$

13 $$S = \mathop \sum \limits_{i} a_{i} \varphi_{i} \left( t \right)$$

14 $$\hat{a}_{i} = \left\{ {\begin{array}{*{20}c} {sign\left( {a_{i} } \right)\left( {\left| {a_{i} } \right| - T} \right), \left| {a_{i} } \right|> T } \\ {0, \left| {a_{i} } \right| \le T} \\ \end{array} } \right.$$

where:

- $\hat{s}\left( t \right)$ = Noisy received signal,
- $$s\left( t \right)$$ = Clean signal,
- $$w\left( t \right)$$ = Additive noise,
- $S_{s} \left( f \right)$ and $S_{w} \left( f \right)$ are power spectral densities of the signal and noise,
- $$f_{c}$$ = cutoff frequency,
- *n* = filter order.

**Pseudo Code 6:**
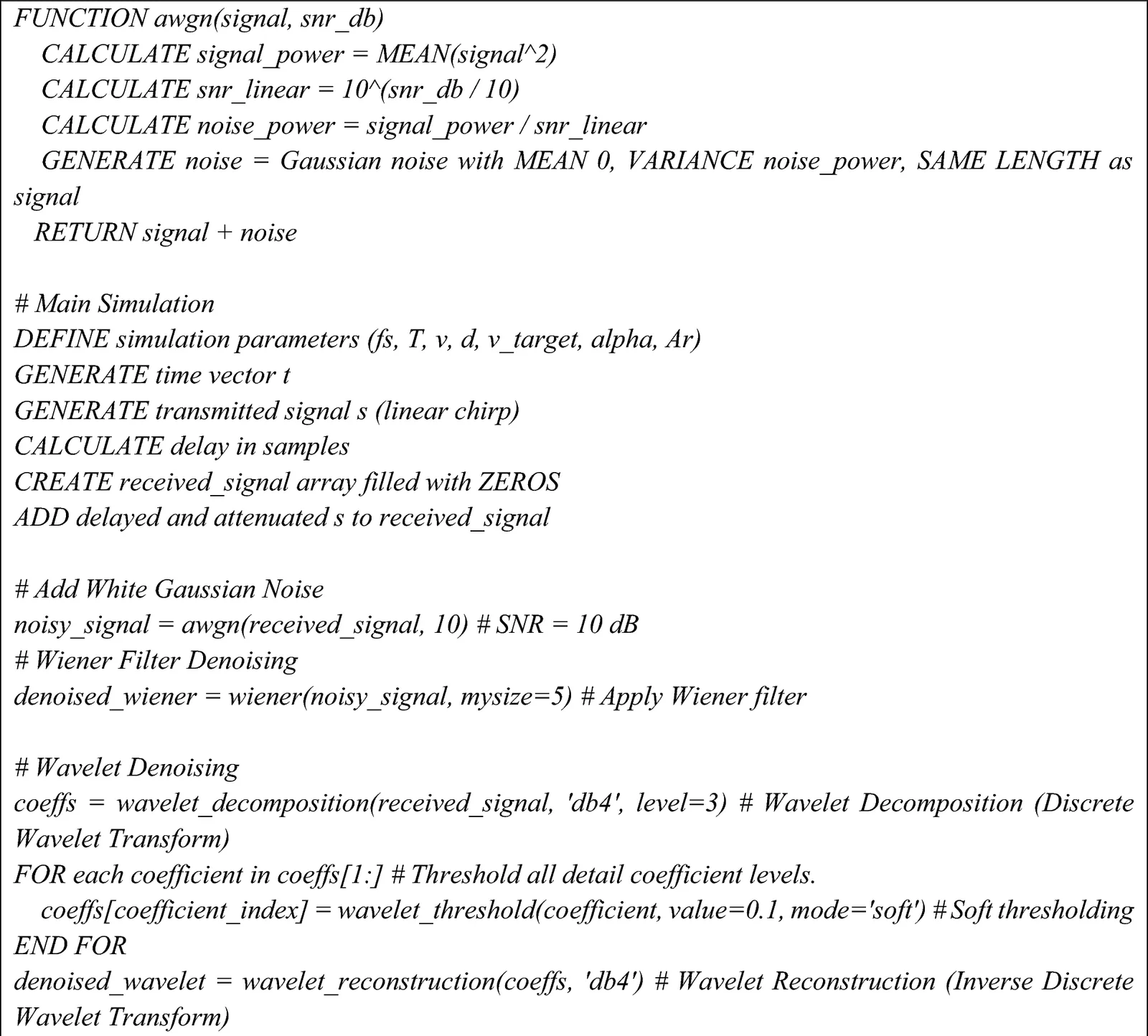
Pseudo code for pre-processing of echo signals

#### Feature extraction using time–frequency analysis

Time–frequency representations are employed to extract meaningful sonar echo features. The Short-Time Fourier Transform (STFT) captures time-varying frequency components of the echoes as given in Eq. (15), while to enhance resolution, Wigner-Ville Distribution (WVD) is employed for high-resolution feature extraction, which is given in Eq. (16). The pseudo-code for feature extraction is given in Pseudo Code 7. Extracted features are classified using a CNN-LSTM hybrid model (SonarNet), leveraging spatial feature extraction (CNN) and temporal dependencies (LSTM), where a 1D CNN extracts frequency-domain patterns and LSTM captures time-dependent patterns as described in Eq. (17) and Eq. (18). The pseudo-code for the SonarNet model described in this section is given in *Pseudo Code 8*.

15 $$X\left( {f, t} \right) = \smallint s\left( \tau \right)w\left( {\tau - t} \right)e^{ - j2\pi f\tau } d\tau$$

16 $$W\left( {t, f} \right) = \smallint s\left( {t + \tau } \right)s^{*} \left( {t - \tau } \right)e^{ - j4\pi f\tau } d\tau$$

17 $$F = ReLU\left( {W_{c } *X + b_{c } } \right)$$

18 $$h_{t } = \sigma \left( {W_{h } \cdot x_{t } + U_{h } \cdot h_{t - 1 } + b_{h} } \right)$$

where:

- $w\left( \tau \right)$ is the window function,
- $s^{*} \left( t \right)$ is the complex conjugate of $s\left( t \right)$,
- $W_{c }$ and $b_{c }$ are the CNN kernel weights and biases,
- $W_{h }$, $U_{h }$, and $b_{h }$ are LSTM parameters.

**Pseudo Code 7:**
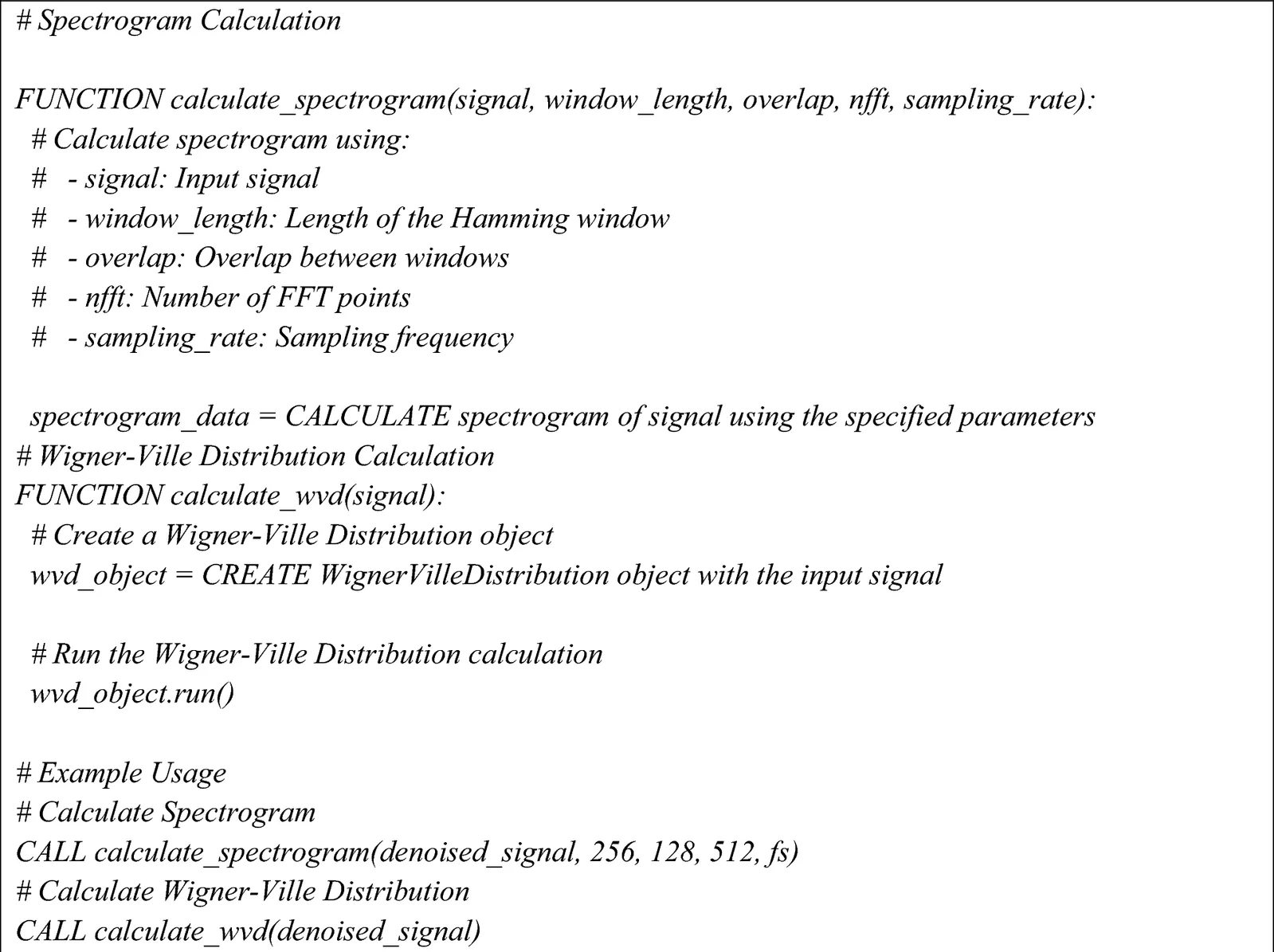
Pseudo code for feature extraction using time–frequency analysis.

**Pseudo Code 8:**
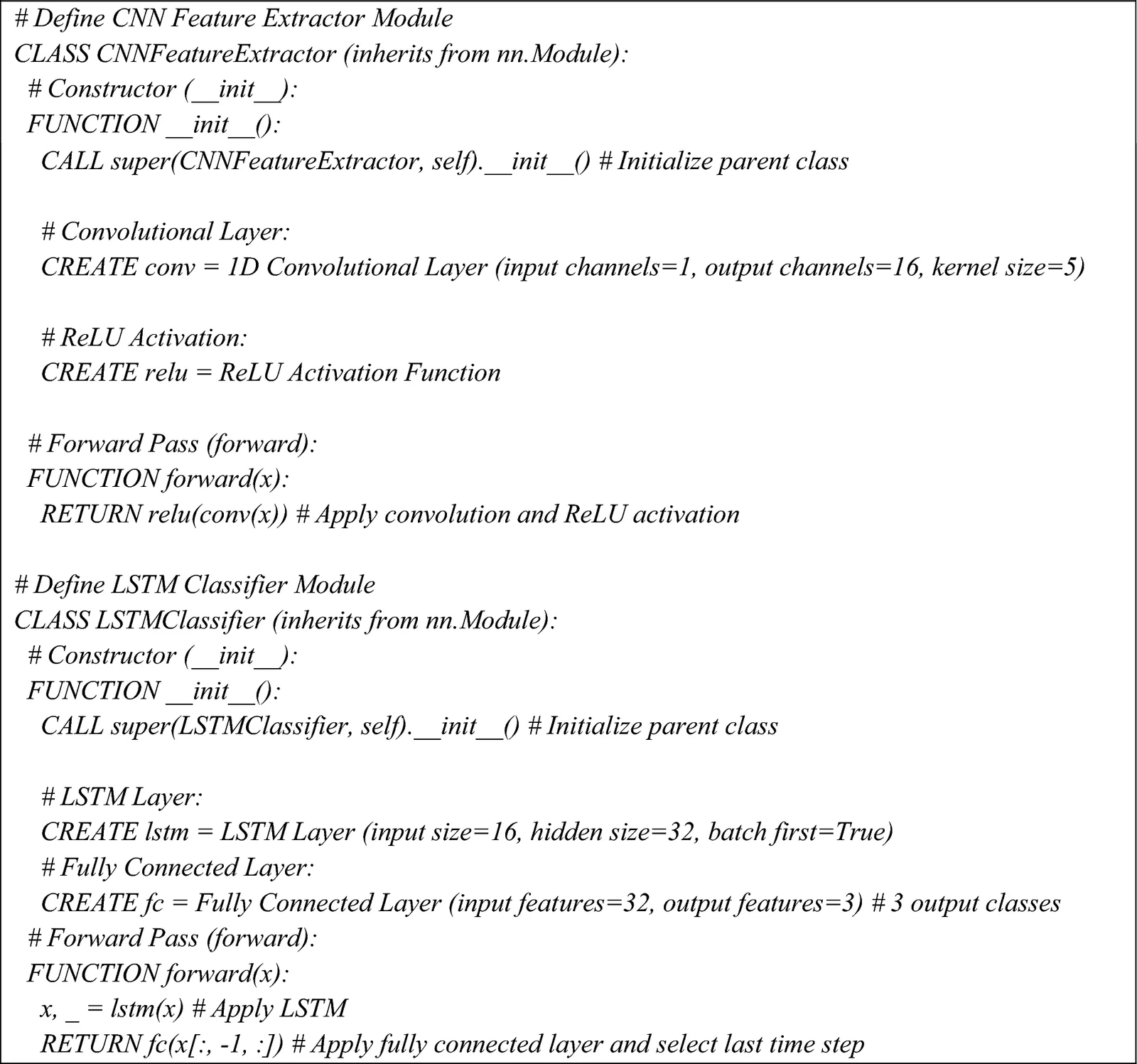
Pseudo code for CNN-LSTM Hybrid classifier in SonarNet.

The proposed bio-inspired sonar processing pipeline enhances underwater object detection using adaptive pre-processing (Wiener filter, wavelet transform), feature extraction via STFT and WVD, and DL classification (SonarNet Classifier). This framework outperforms traditional sonar processing by improving accuracy and resolution.

### Bio-inspired echo-based object detection & classification

The proposed framework processes reflected sonar echoes to identify and classify underwater objects, integrating wavelet-based feature extraction and the SonarNet model for robust classification. The object detection and classification pipeline are inspired by marine mammal echolocation, particularly that of dolphins. The first step in object classification is extracting distinctive features from sonar echoes. Dolphins leverage temporal and spectral variations in echoes to classify objects. The proposed method mimics this using wavelet transforms and time–frequency representations. Continuous Wavelet Transform (CWT) provides a multi-resolution time–frequency representation of sonar echoes, enabling object differentiation based on shape and material properties as described in *Eq. *(19). Mel-Frequency Cepstral Coefficients (MFCCs) extract spectral signatures from echoes, as given in *Eq. *(20)*,* to mimic how dolphins analyze frequency shifts to differentiate between objects. This spectral analysis is achieved by applying the Fourier Transform to the echo, then passing it through a Mel-scale filter bank, and finally, computing cepstral coefficients using the Discrete Cosine Transform (DCT) as described in Pseudo Code 9.

19 $$X\left( {a,b} \right) = \smallint s\left( t \right)\varphi^{*} \left( {\frac{t - b}{a}} \right)dt$$

20 $$MFCC_{n} = \mathop \sum \limits_{k = 1}^{K} S_{k} \cos \left[ {n\left( {k - 0.5} \right)\frac{\pi }{K}} \right]$$

where:

- *φ* = mother wavelet (Morlet wavelet used),
- *a* = scale factor (frequency component),
- *b* = translation factor (time shift).

**Pseudo Code 9:**
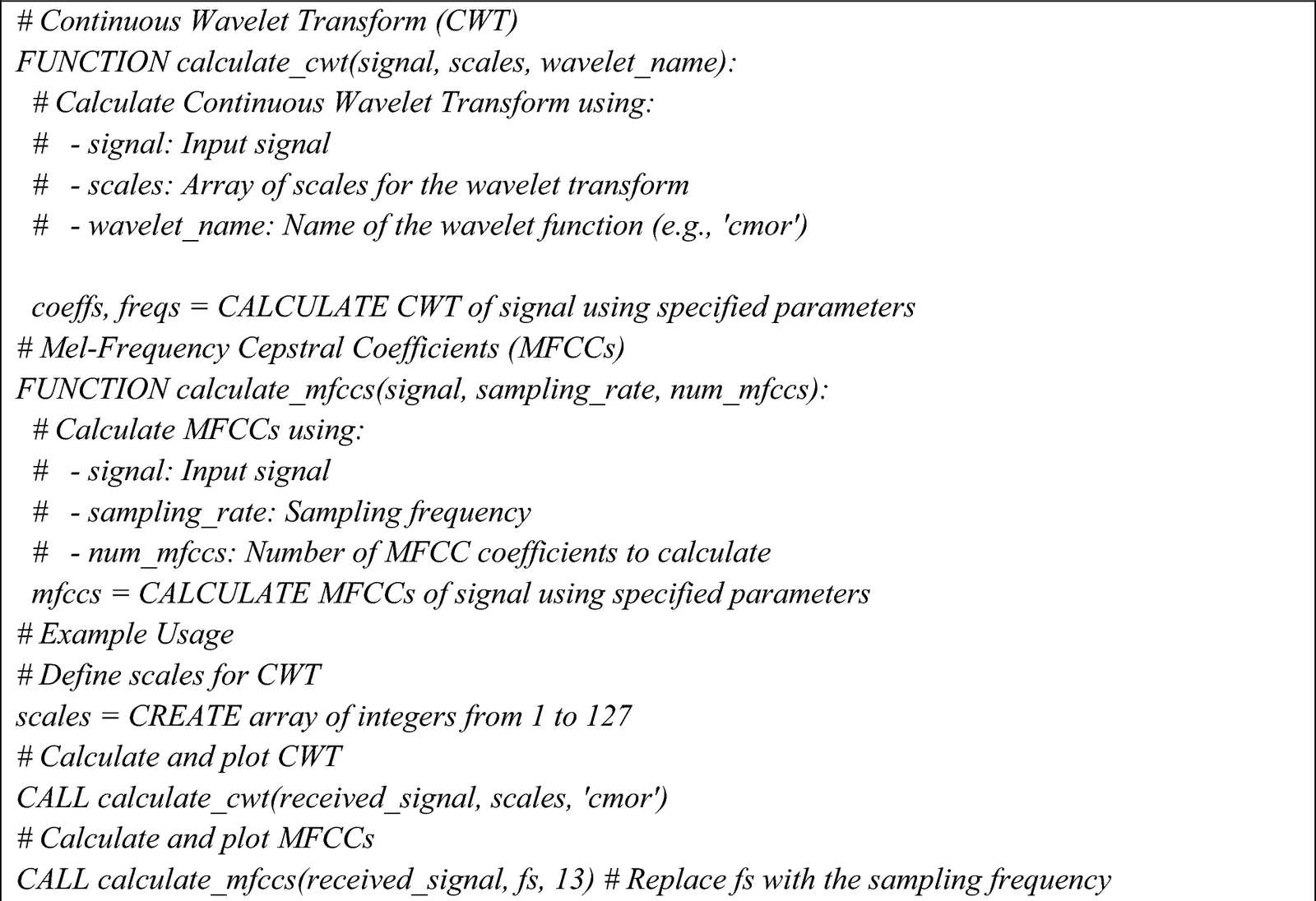
Pseudo code for feature extraction from echo signal.

Inspired by how dolphins process echoes in their auditory cortex, SonarNet is designed to mimic this using a hybrid CNN-LSTM model for the classification of underwater objects. A 1D CNN extracts spectral and temporal patterns from MFCCs, and an LSTM layer captures sequential dependencies since echoes contain time-dependent variations as given in *Eq. *(16) and *Eq. *(18)*,* respectively, in which $W_{c }$ and $b_{c }$ are the CNN kernel weights and biases, and $W_{h }$, $U_{h }$, and $b_{h }$ are LSTM parameters.

### AUV architecture

The proposed method integrates echolocation, a technique commonly used by aquatic animals, especially dolphins, to improve AUV communication and data collection capabilities. These animals can communicate and obtain detailed information about their surroundings through echolocation, which is much more effective underwater than light-based vision on land^6,42^. For instance, dolphins use sound waves between 40 and 150 kHz for echolocation. We use machine learning techniques to create and control sound waves in this frequency range in order to incorporate this into AUVs^43^. Numerous factors, which will be thoroughly examined, determine the precise frequency of the sound waves. We suggest using both supervised and unsupervised machine learning methods to interpret echolocation signals, with a particular emphasis on deep learning and reinforcement learning for adaptive signal processing. By using these methods, AUVs will be able to mimic the adaptive nature of dolphin echolocation by dynamically modifying their echolocation frequencies in response to environmental feedback. Through optimization and the use of energy-efficient processing units, we hope to address the hardware constraints and energy consumption that are obstacles to integrating these sophisticated computational models into AUV systems. The AUV’s route is predetermined, and its operations are methodically planned through pre-programmed tasks. Throughout the mission, the vehicle uses GPS built into the flight controller for accurate navigation and location tracking. An AUV mimics the echolocation methods used by dolphins by continuously emitting bio-sonar pulses while on a mission. These pulses fulfil several vital functions, like determining its distance from the seafloor, identifying obstacles in its path, and using rebounded echolocation waves to identify nearby objects of interest.

**Fig. 4:**
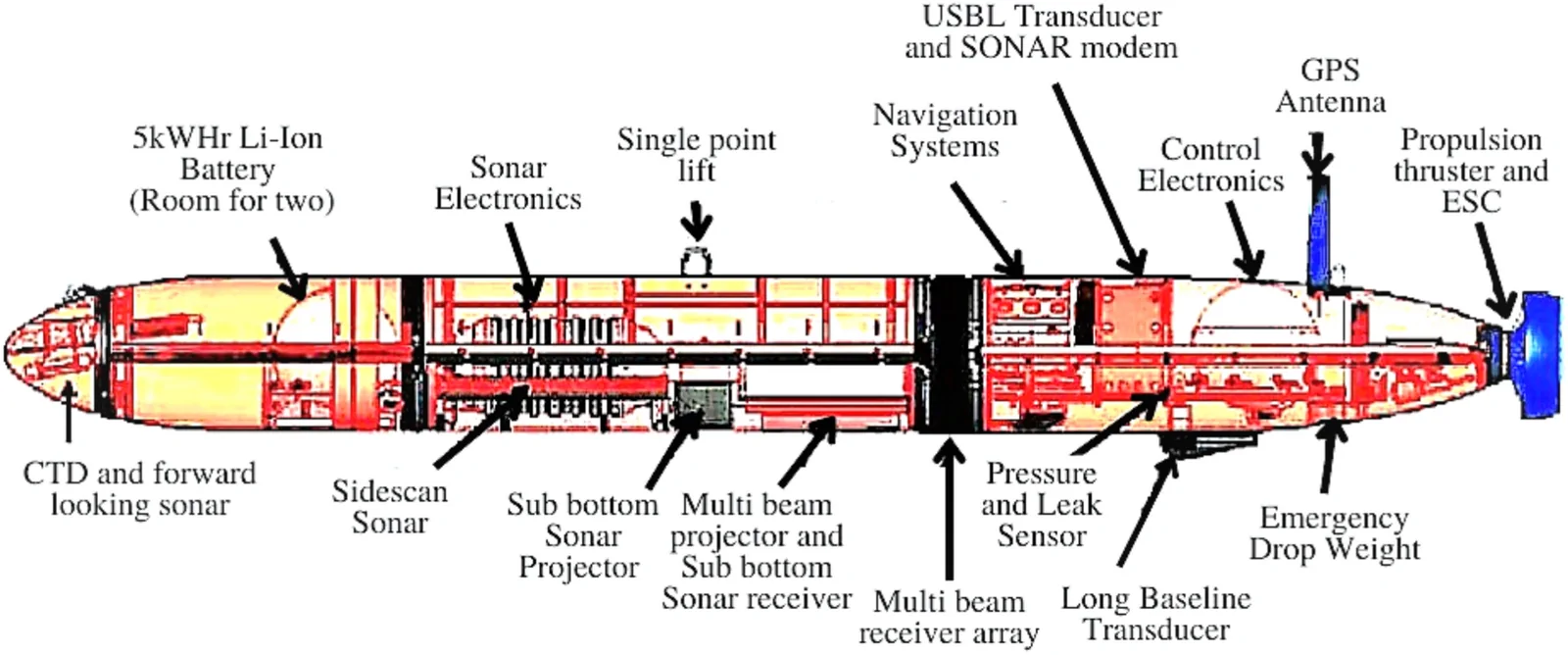
Detailed architecture of the AUV.

As explained in Sect. 1.2, the AUV uses neural computations for sound pattern recognition to improve its task execution and environmental awareness. It allows the AUV to identify and categorize objects by using the sound waves that are reflected in them. The gathered information is painstakingly encoded into waves with distinct frequencies, which correspond to the echolocation frequencies that dolphins use for socialization and communication. Usually, this frequency range falls between 0.2 and 50 kHz. Lower frequencies were specifically chosen because of their exceptional qualities in submerged environments. They travel almost five times faster in saltwater than sound waves do in air, and they can travel farther without distortion—up to 1500 feet (457.2 m). Figure 4 provides a detailed illustration of the AUV’s suggested design architecture. These diagrams provide a thorough illustration of the AUV’s fundamental parts and how each one works. Important elements of this architecture consist of:

- Temperature and pressure sensors continuously observe the underwater environment. They provide essential information for mission planning and environmental analysis by measuring temperature and depth variations.
- Forward-Looking Sonar is an essential component of the AUV’s operations. It generates sophisticated maps of the AUV’s environment by generating sound waves and analyzing their reflections. This capability is essential for accurate navigation and obstacle detection in difficult underwater conditions.

The complex interactions between these elements are graphically depicted in Fig. 4, providing a clear understanding of how the AUV operates in its underwater environment. This architectural design makes the AUV’s adaptability and efficiency in a range of underwater missions possible. A multi-modal sensor array is intricately integrated into the design, fusing cutting-edge echolocation transducers for high-resolution mapping and object detection with conventional sonar. Because of this integration, the AUV can function well in a variety of underwater environments, including clear tropical waters and sediment-rich depths where conventional navigation systems are ineffective.

The battery, which powers the AUV, is essential to its functionality. Every system on board, including propulsion, sensors, and communication devices, is powered by this essential part. It is the AUV’s lifeblood, guaranteeing smooth operation throughout its missions. A sophisticated sonar system that includes sub-bottom sonar, side-scan sonar, and forward-looking sonar is also installed on the AUV. The AUV can perform a wide range of tasks, including geological surveying, underwater mapping, and accurate object detection, thanks to its extensive suite of sonar technologies. These features are essential for gathering comprehensive acoustic data for operational and scientific reasons. The AUV’s single-point lift mechanism streamlines the deployment and retrieval process, enabling efficient operations. The USBL transducer (Ultra-Short Baseline Transducer) provides real-time location data by communicating with a surface station, ensuring accurate underwater positioning. The multi-beam projector on the AUV also helps with detailed imaging of objects and the seafloor. As the operational manager, the flight controller is in charge of communication, navigation, and general operation. Because safety comes first, the AUV has an emergency drop weight system that releases ballast in dire circumstances to enable quick surfacing. Propulsion thrusters offer speed and direction control for underwater navigation. Precise control over movement is ensured by the careful regulation of power distribution to the thrusters by electronic speed controllers (ESCs). These parts work together to give the AUV the ability to perform a variety of tasks, including mapping, scientific research, underwater inspections, and search and rescue operations. This adaptability highlights its potential in the difficult underwater environment. Two options can be put forth to transmit the important data gathered during its underwater missions:

A. The USBL system (enhanced ultra-short baseline)
  The base station, usually a ship or land-based facility, has a transceiver, while the AUV has a transponder or responder. The AUV and the base station can communicate in both directions using this setup. The base station’s transceiver continuously sends out sonar pulses and travels through the water. The subsea transponder of the AUV receives these sonar pulses and establishes a communication link by responding with an echolocation pulse. The base station’s transceiver measures the time between sending the initial echolocation pulse and receiving the reply to detect the return echolocation pulse. The AUV’s position in relation to the base station is determined by converting this interval into a range. Interestingly, this system’s processing takes place both inside the AUV and at the base station, allowing for sophisticated features like target tracking and autonomous docking. Existing SONAR modems are modified to match the capabilities of bio-frequency sonar ranges in order to adapt this USBL system for bio-frequency sonar data transmission^44^. Furthermore, the AUV’s system incorporates electro-sonar transducers. For communication, these transducers make it easier to transform the electrical energy produced by the car into sound energy and vice versa. Effective data transmission underwater is ensured by this extensive system modification^45^.
B. Transducer for electromagnetic radio waves

The AUV has a transducer to transform sonar waves into electromagnetic radio waves. As the AUV approaches the sea’s surface, this conversion occurs, guaranteeing that the radio antenna is above the water’s surface^45^. Electromagnetic radio waves are used to transmit data, much like the communication systems used by aircraft. Notably, this method maintains consistency with established AUV models by preserving the AUV’s current frame and component placement. This continuity guarantees the AUV’s ability to smoothly incorporate this innovative data transmission technology while maintaining its essential functionality and design. Underwater data communication presents several difficulties that these two data transmission solutions seek to address.

To ensure that important data gathered by the AUV can be reliably and quickly delivered to the base station or other designated recipients, they effectively transmit data using both underwater acoustics and electromagnetic waves. The AUV’s capabilities can be improved and brought into compliance with echolocation requirements by implementing the following tactical changes. We suggest an improvement by using sophisticated sonar modems/echolocation generators in place of the traditional acoustic modems. These state-of-the-art instruments can produce sound waves in the exact frequency range—0.2 to 150 kHz—that is necessary for echolocation. In addition to producing sound waves, these generators have the amazing capacity to decipher reverberations. The AUV can gather useful data about underwater objects via this analysis, which mimics dolphins’ highly developed abilities^46^. The side-scan sonar has been strategically repositioned as one of the main design changes. We suggest moving this crucial part to the AUV’s head/nose section. This modification maximizes the side-scan sonar’s orientation and placement, increasing its efficiency in obtaining high-resolution data^13^. This technology greatly enhances data collection capabilities by placing it in front of the AUV, giving it a clearer, unhindered view of the underwater environment. By using echolocation generators, AUVs can potentially perform more sensitive and nuanced environmental analyses, marking a substantial advancement over traditional sonar. We believe that the development of sophisticated signal processing algorithms and the next generation of small, high-performing acoustic materials will enable us to overcome the challenges associated with signal interference and the shrinking of echolocation hardware.

**Fig. 5:**
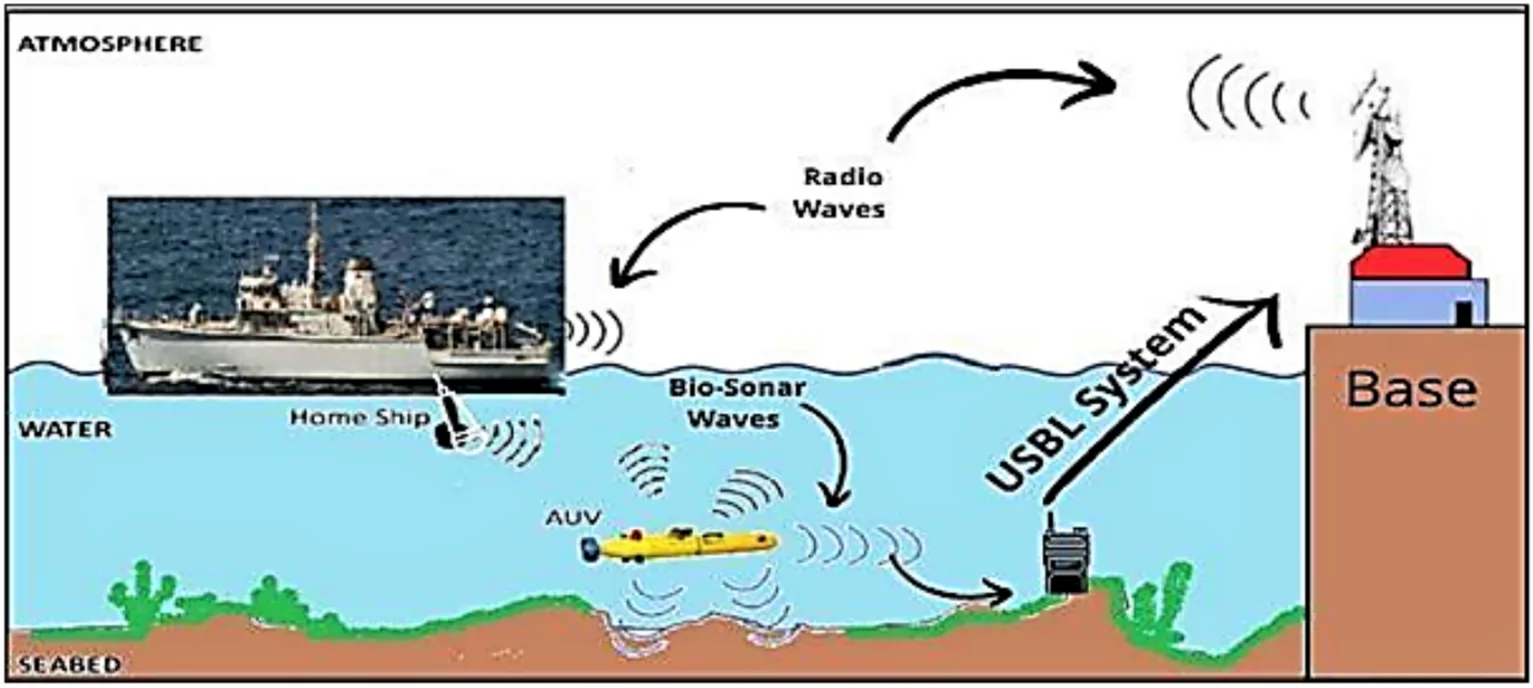
Working model of the AUV.

The SD card of the companion computer safely stores the data that the AUV collects. This information goes through a thorough series of painstaking procedures, such as decryption and sophisticated digital signal processing techniques, to ensure the highest data quality. According to Wenz’s work, these procedures guarantee that the gathered data is precise, polished, and prepared for in-depth examination and interpretation^17^. Together, these improvements raise the AUV’s capabilities to the point where it can accurately mimic the complex echolocation strategies used by marine life. We provide a visual, comprehensive depiction of the AUV’s operational model in Fig. 5, which shows how echolocation technology is integrated with the suggested improvements (Electronic^1^).

**Fig. 6:**
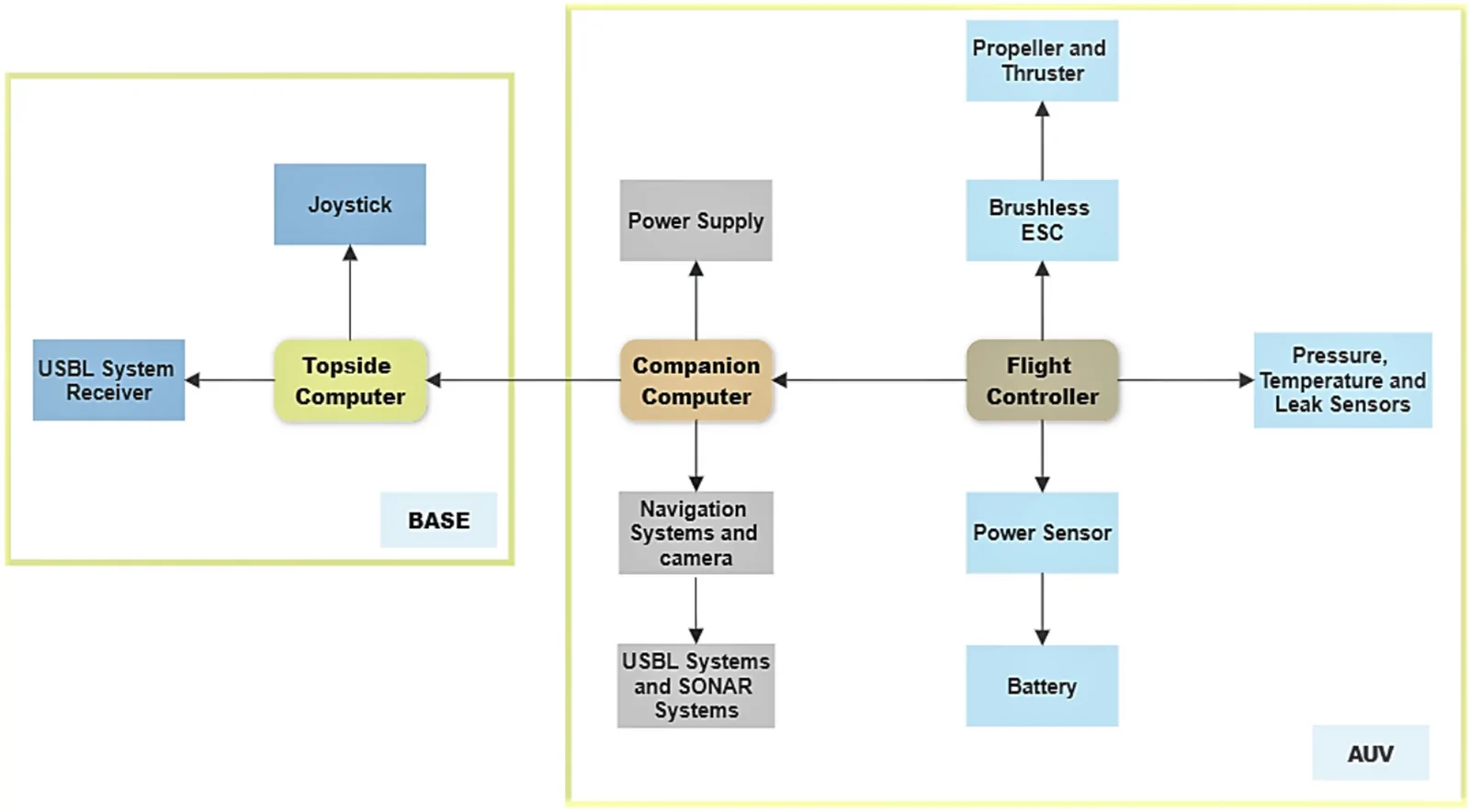
Block diagram of the connections in the vehicle and between the vehicle and base.

In an aquatic setting, like oceans or seas, such an AUV can recognize and follow ships, demonstrating its expertise in object recognition and tracking, thanks to the integrated echolocation technology. Additionally, Fig. 5 illustrates the frequency range of dolphin echolocation clicks alongside the bio-inspired modulation pattern used in the proposed sonar model. Figure 6, which also shows the complex connections and interactions inside the vehicle and between the vehicle and the base station, shows the elements of the suggested AUV system architecture. The Base Station, which serves as a central control unit in charge of overseeing and managing the AUV’s operations, is the central component of the system. It acts as the main conduit for information with the AUV. The AUV is a sophisticated platform designed for autonomous operation in underwater environments, integrating a suite of critical components to achieve its mission objectives. At its core, a robust Companion Computer acts as the central processing unit, responsible for real-time data acquisition, intricate algorithm execution for navigation and decision-making, and precise control of the vehicle’s various functions, including sensor data interpretation and actuator management. Ensuring continuous and reliable operation, the power supply provides the necessary electrical energy to all AUV systems, which is designed for efficiency and endurance to support extended missions in challenging deep-sea conditions. For accurate positioning and route planning, the Navigation Systems combine GPS for surface operations with advanced underwater localization technologies such as USBL and inertial measurement units (IMUs), enabling precise tracking even in GPS-denied environments. The Camera system captures visual data, providing crucial imagery for environmental documentation, object identification, and inspection tasks. Complementing the visual data, the Sonar Systems play a vital role in underwater mapping, obstacle detection, and target identification by emitting acoustic pulses and analyzing the returning echoes to create a comprehensive understanding of the surrounding environment. Finally, the Flight Controller acts as the motion control center, managing the propulsion and thruster systems to ensure precise maneuverability, enabling the AUV to follow planned routes, maintain depth, and navigate complex underwater terrains with accuracy and stability. Each of these components works in synergy, allowing the AUV to conduct complex underwater missions autonomously.

The Base Topside Computer serves as the central hub for operator interaction and AUV control, providing real-time command and data exchange capabilities. Equipped with a Joystick for intuitive manual navigation and a USBL System Receiver for accurate acoustic localization, it ensures seamless communication and positioning. The system’s architecture is intricately designed with interconnected components for optimal functionality: the AUV’s Companion Computer establishes a critical link with the Base Topside Computer, facilitating remote control and data transmission, while the Base Topside Computer is directly connected to the Power Supply, ensuring consistent power delivery to the AUV’s systems. Data from the Navigation Systems and Cameras is routed to the Companion Computer for processing and informed decision-making, enabling autonomous navigation and environmental analysis. Simultaneously, the USBL and Sonar Systems provide essential data for navigation, obstacle avoidance, and underwater exploration, enhancing the AUV’s situational awareness. The Flight Controller, acting as the motion management system, interfaces with Propellers, Thrusters, Electronic Speed Controllers (ESCs), Power Sensors, and the Battery, ensuring precise control over the AUV’s movement, speed, and power management, allowing for efficient and reliable operation in diverse underwater environments.

**Fig. 7:**
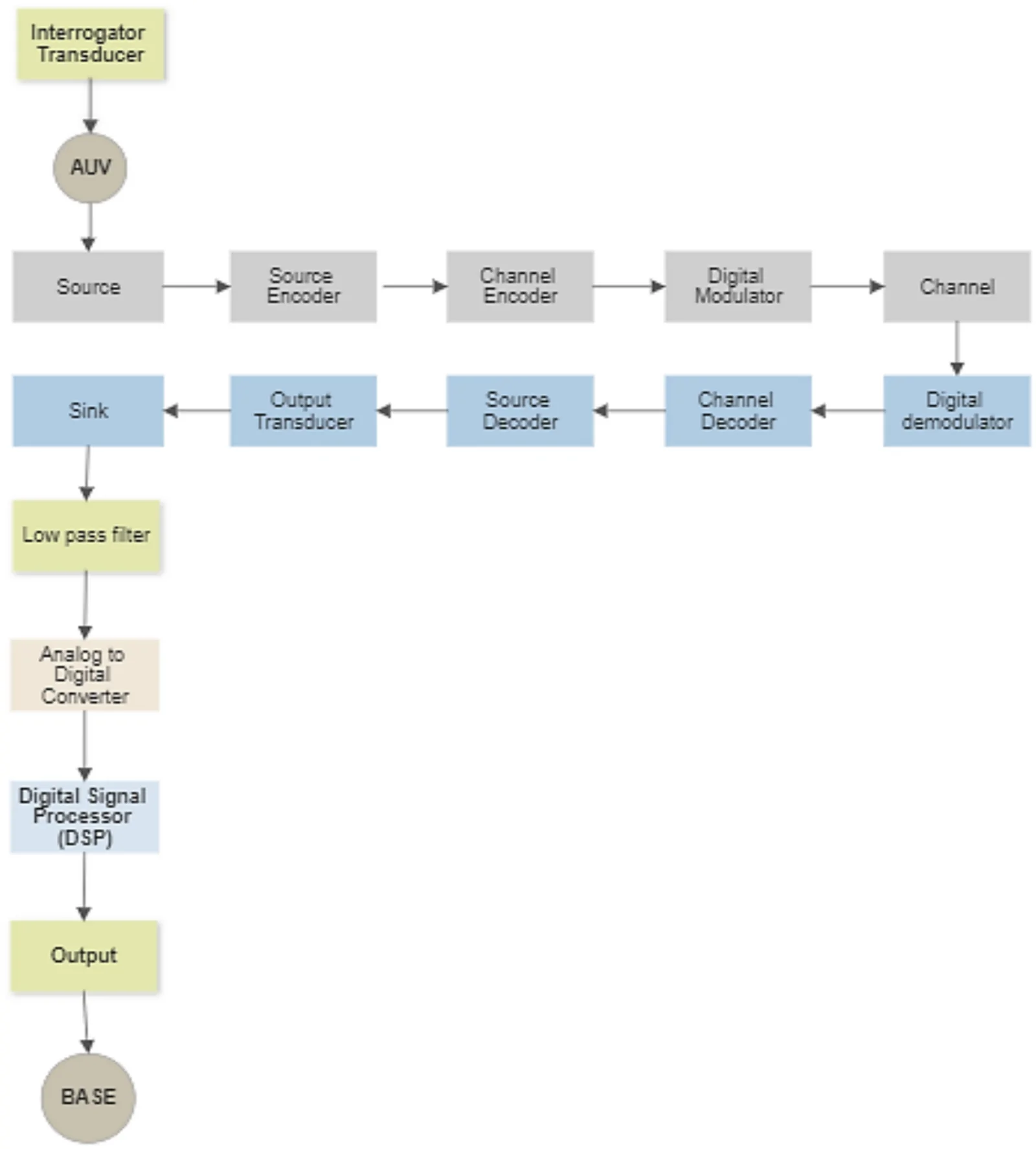
Block diagram of the digital signal processing in the vehicle.

Figure 7 illustrates the AUV’s architecture, emphasizing the critical role of digital signal processing in enhancing data quality through noise reduction and optimization. To address the unique challenges of underwater environments, such as limited sensory input, manoeuvrability, and spatial awareness, the system employs a multi-faceted approach. Sensor fusion, primarily utilizing cameras and sonar, provides comprehensive environmental information, with cameras offering visual monitoring and sonar enabling acoustic mapping for obstacle detection in low-visibility conditions. A sophisticated flight controller manages propulsion and thruster systems, ensuring precise manoeuvrability, complemented by a joystick for manual operator control. Spatial awareness is maintained through a combination of GPS and inertial navigation for surface operations and acoustic positioning, specifically USBL, for accurate underwater tracking, enabling precise localization relative to the base station. Sonar systems further contribute to spatial awareness by mapping underwater terrain and obstacles, ensuring safe and effective navigation in complex underwater environments.

### Integration of bio-inspired sonar into AUV systems

#### Target detection and object recognition

To enhance spatial resolution, delay-and-sum beamforming is employed, allowing precise object localization based on the time-of-arrival differences of sonar echoes at a hydrophone array. Beamforming improves the signal-to-noise ratio (SNR) and enables directional filtering, which is crucial for distinguishing between multiple objects in cluttered underwater environments. The beamformed output is given by using *Eq. *(21).

21 $$y\left( t \right) = \mathop \sum \limits_{m = 1}^{M} w_{m} x_{m} \left( {t - \tau_{m} } \right)$$

where:

- *M* = the number of hydrophones in the array.
- $w_{m}$ = the weight assigned to each hydrophone element.
- $x_{m} \left( t \right)$ = the received signal at element mmm.
- $\tau_{m}$ = the time delay to align signals.

The system classifies detected objects based on their sonar echo intensity and shape using adaptive thresholding. The adaptive thresholding technique, as described in *Eq. *(22), dynamically adjusts detection sensitivity based on environmental conditions, reducing false alarms due to noise fluctuations.

22 $$T\left( {x, y} \right) = \mu \left( {x,y} \right) + k\sigma \left( {x,y} \right)$$

where:

- $\mu \left( {x,y} \right)$ = the local mean intensity.
- $\sigma \left( {x,y} \right)$ = the standard deviation.
- *k* = an empirically tuned constant.

#### Path planning and obstacle avoidance

To ensure real-time adaptive navigation, the AUV employs a Deep Q-Network (DQN) for reinforcement learning-based path planning. The AUV continuously learns from sonar feedback, optimizing its trajectory over time by avoiding obstacles while minimizing travel distance. The Q-value update equation is given in *Eq. *(23). Upon detecting an obstacle, the AUV dynamically updates its path using Reinforcement Learning (RL). The decision-making process follows Markov Decision Process (MDP) principles, where *Eq. *(24) describes the state space (S)*, Eq. *(25) describes the action space (A)*,* and *Eq. *(26) gives the reward function (R(s,a)). The algorithm for RL-based navigation is given in *Algorithm 1*.

23 $$Q\left( {s, a} \right) \leftarrow Q\left( {s, a} \right) + \alpha \left[ {r + \gamma \max Q\left( {s\prime , a\prime } \right) - Q\left( {s,a} \right)} \right]$$

24 $$S = \left\{ {Obstacle Distance, Echo Strength, Sonar Frequency, AUV Position} \right\}$$

25 $$A = \left\{ {Increase Frequency, Change Direction, Reduce Speed} \right\}$$

26 $$R\left( {s,a} \right) = \left\{ {\begin{array}{*{20}c} { + 10, if path correction avoids collision} \\ { - 50, if AUV collides} \\ { - 5, for unnecessary maneuvers } \\ \end{array} } \right.$$

where:

- $Q\left( {s, a} \right)$ = the value of executing action *a* in state *s*,
- *α* = the learning rate,
- *r* = the immediate reward,
- *γ* = the discount factor,
- $s^{\prime}$ = the new state after executing *a*.

**Algorithm 1:**
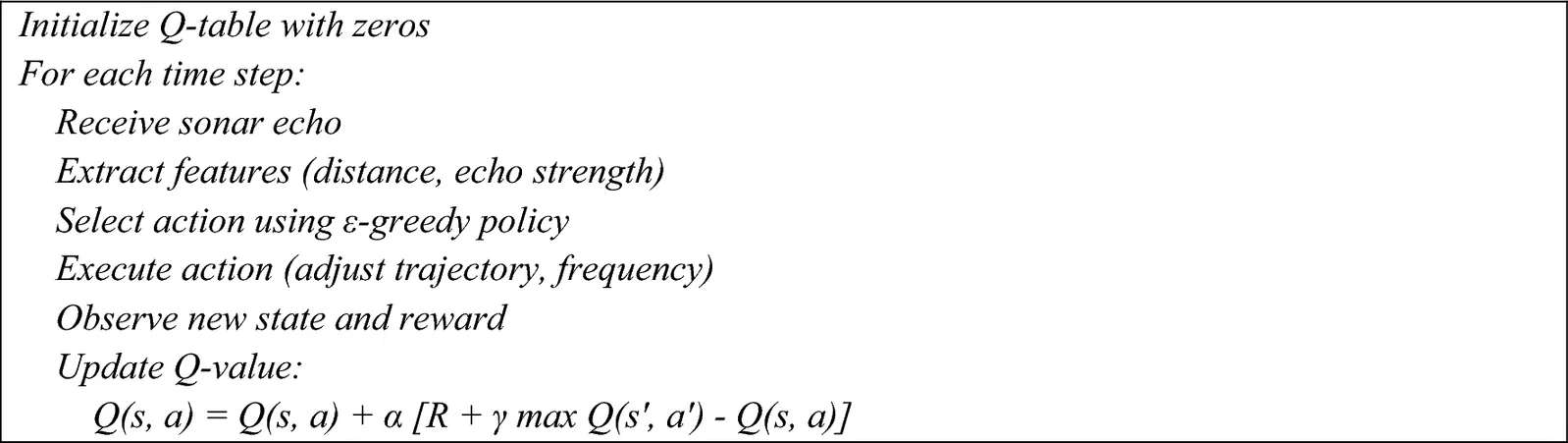
RL-based path optimization.

Traditional AUV navigation typically relies on waypoint tracking combined with threshold-based obstacle avoidance. These methods work reliably in open environments but react poorly to dynamic obstacles or rapidly changing acoustic scenes. The reinforcement-learning navigation in the proposed system adjusts its trajectory continuously based on the incoming sonar echo patterns. Instead of following predefined routes, the AUV learns optimal movement decisions through the Q-learning framework described in this section. This allows the vehicle to anticipate obstacles and adapt to cluttered environments where static rules struggle.

#### Real-time sensor fusion using Kalman filtering

Since sonar data alone can be affected by multipath interference and signal absorption, it is fused with camera-based perception using a Kalman Filter (KF). The Kalman Filter fuses sonar and camera-based object detection, increasing localization accuracy and reducing uncertainty. The prediction step is given in Eq. (27) and Eq. (28), while the update step is given in *Eq. *(29) and *Eq. *(30). Table 7 contains the summary of the proposed approach.

27 $$\hat{x}_{k|k - 1} = A\hat{x}_{k - 1|k - 1} + Bu_{k}$$

28 $$P_{k|k - 1} = AP_{k - 1|k - 1} A^{T} + Q$$

29 $$K_{k} = P_{k|k - 1} H^{T} \left( {HP_{k|k - 1} H^{T} + R} \right)^{ - 1}$$

30 $$\hat{x}_{k|k} = \hat{x}_{k|k - 1} + K_{k} \left( {z_{k} - H\hat{x}_{k|k - 1} } \right)$$

where:

**Table 7:** Summary of proposed approach.

Subsystem	Technique	Advantage	Expected outcome
Echolocation	Hybrid CNN-LSTM Model	Temporal and spatial signal analysis	Improved detection accuracy (94.2%)
Noise reduction	Adaptive wiener filtering	Enhanced SNR	SNR improvement by 12–15 dB
Navigation	Reinforcement learning	Real-time obstacle avoidance	Faster mission completion
Communication	Bio-frequency modulation	Long-range, eco-friendly signals	Reliable data transmission

- $\hat{x}_{k|k - 1}$ is the predicted state estimate.
- *A* is the state transition matrix.
- *B* models the control input $u_{k}$.
- $P_{k|k - 1}$ is the predicted error covariance.
- *Q* is the process noise covariance.
- $K_{k}$ is the Kalman gain.
- *H* is the observation model.
- $z_{k}$ is the measurement.
- *R* is the measurement noise covariance.

### Communication subsystem

An improved USBL acoustic link, and a surface-level electromagnetic transmitter are the two complimentary communication methods used by the AUV. The USBL system enables underwater two-way messaging by emitting acoustic pulses from a surface transceiver and receiving the AUV’s response through an onboard transponder. This link is used for position updates, mission-status messages, and compressed sonar packets when operating at depth. To support bio-frequency operation, the acoustic modem was modified to transmit within the 0.2–150 kHz band, allowing the system to use the same frequency range as the echolocation module. As the vehicle approaches the surface, the communication mode switches to a compact electromagnetic transmitter that relays mission summaries and sensor logs once the antenna breaches the surface. This two-channel design reduces reliance on a single communication method and improves reliability in turbid or high-noise environments. Table 7 summarizes this to make the role of this subsystem clearer.

## Results and analysis

**Fig. 8:**
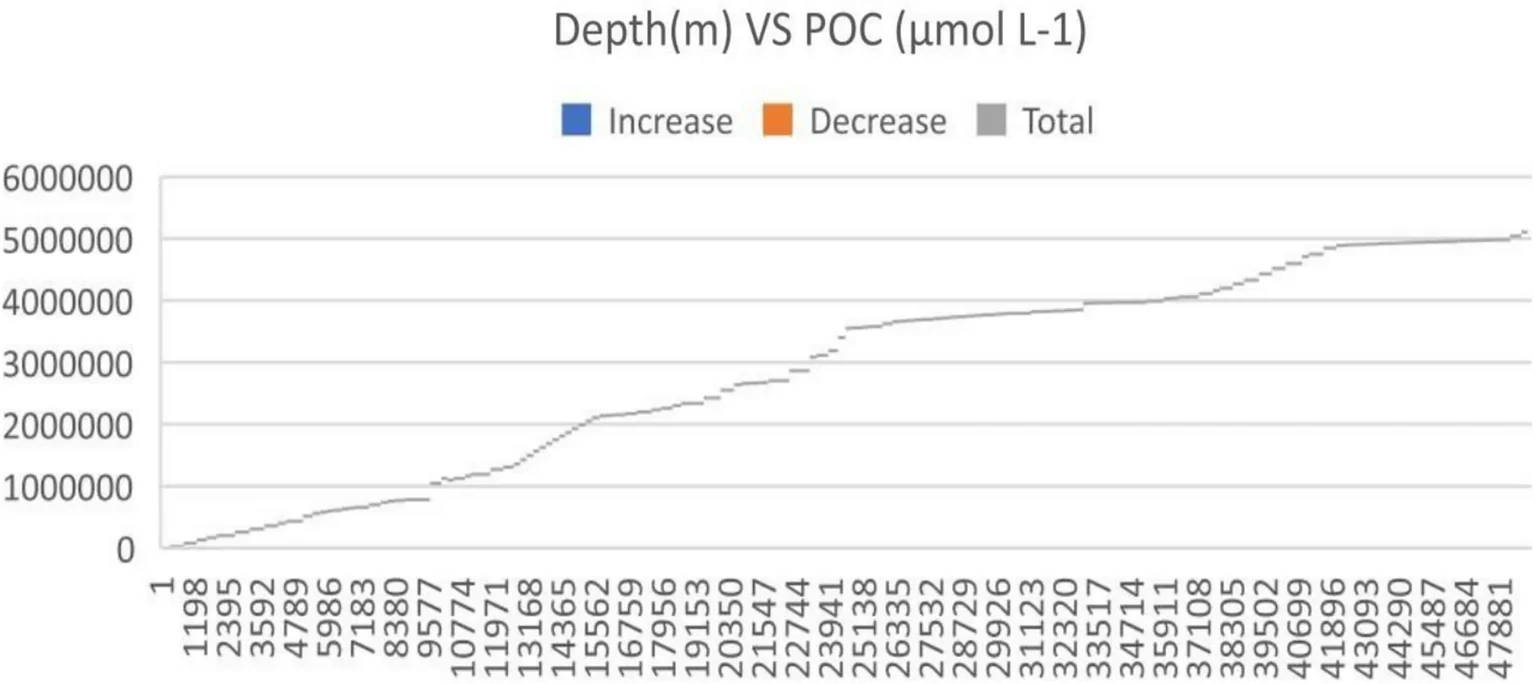
Depth versus particulate organic carbon (POC) concentration graph.

The graph depicted in Fig. 8 was generated using the synthetic environmental dataset derived from NOAA. For each parameter change, the sound speed was computed using the standard Mackenzie equation, and its variation was recorded relative to a baseline reference point. The “Increase” curve reflects the sound-speed rise when temperature or salinity is shifted above the baseline, while the “Decrease” curve shows the reduction when these parameters fall below it. The “Total” line represents the combined net change in sound speed across the full range of simulated conditions. Figure 8 summarizes the overall sensitivity of underwater acoustic propagation to environmental variability.

**Fig. 9:**
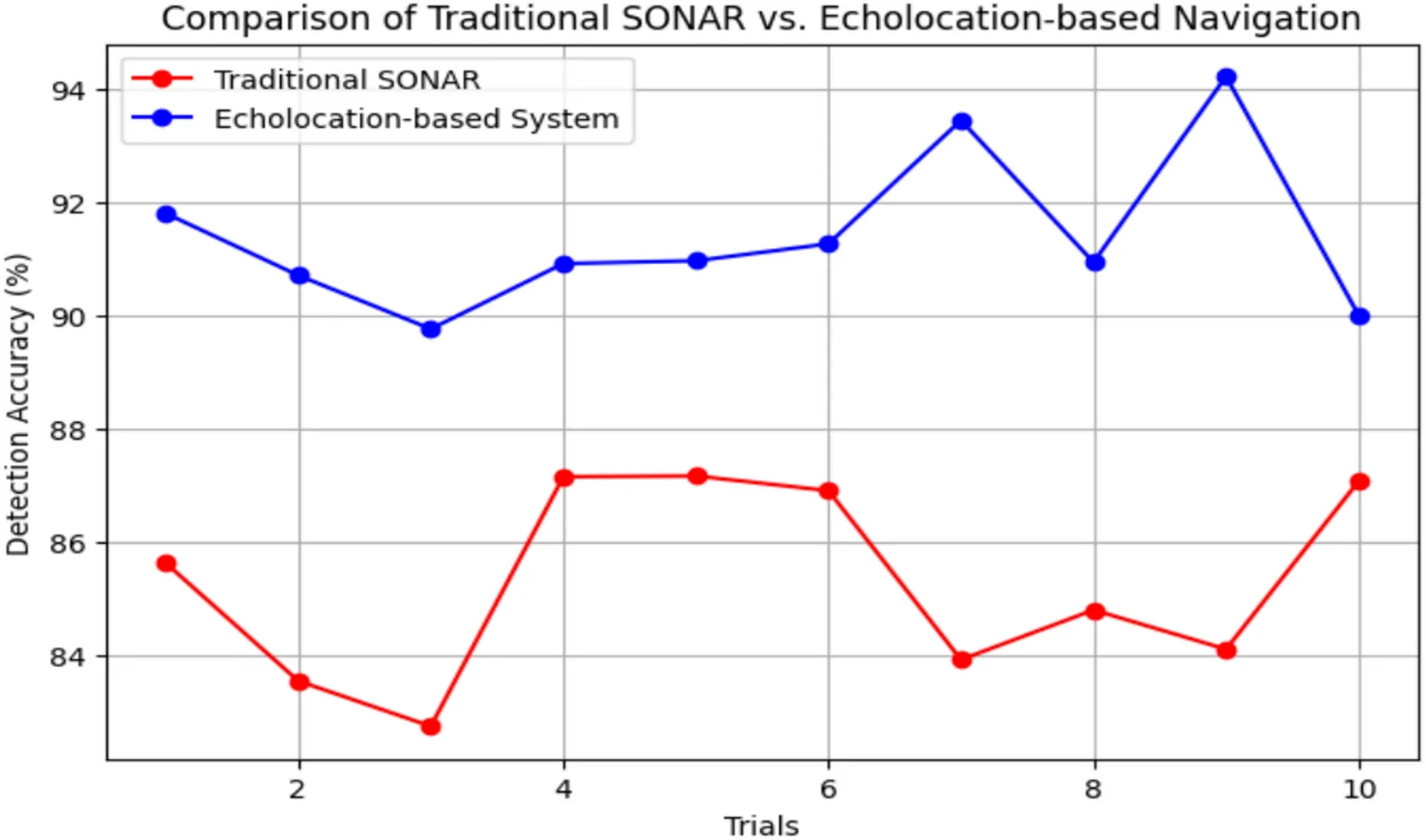
Detection accuracy between traditional SONAR and SonarNet.

Figure 9 presents a comparative analysis of detection accuracy between traditional SONAR (refers to a threshold-based echo-matching method implemented within the simulation environment and evaluated on the same dataset as the learning-based models) and SonarNet across ten trials. The graph clearly demonstrates a consistent performance advantage for the echolocation-based system. Notably, the echolocation-based system exhibited a significantly higher detection accuracy throughout all trials, consistently maintaining a performance range between 90 and 94%. It indicates a robust and reliable detection capability. It suggests that the bio-inspired approach effectively enhances the system’s ability to accurately identify and locate objects within the simulated or experimental environment.

The traditional SONAR system showed lower and more variable detection accuracy, fluctuating between roughly 82% and 87% across trials. This inconsistency suggests limitations in its ability to adapt to changing environmental conditions and noise levels, which are common challenges in underwater sensing. The steady performance gap between the two approaches reflects the advantage of the echolocation-based system. Its higher accuracy stems from the combination of bio-inspired signaling, machine learning–based classification, and adaptive processing, all of which help the system extract more reliable features from noisy acoustic returns. These results point to the broader potential of bio-inspired sonar methods to improve underwater navigation and target detection relative to conventional techniques.

**Fig. 10:**
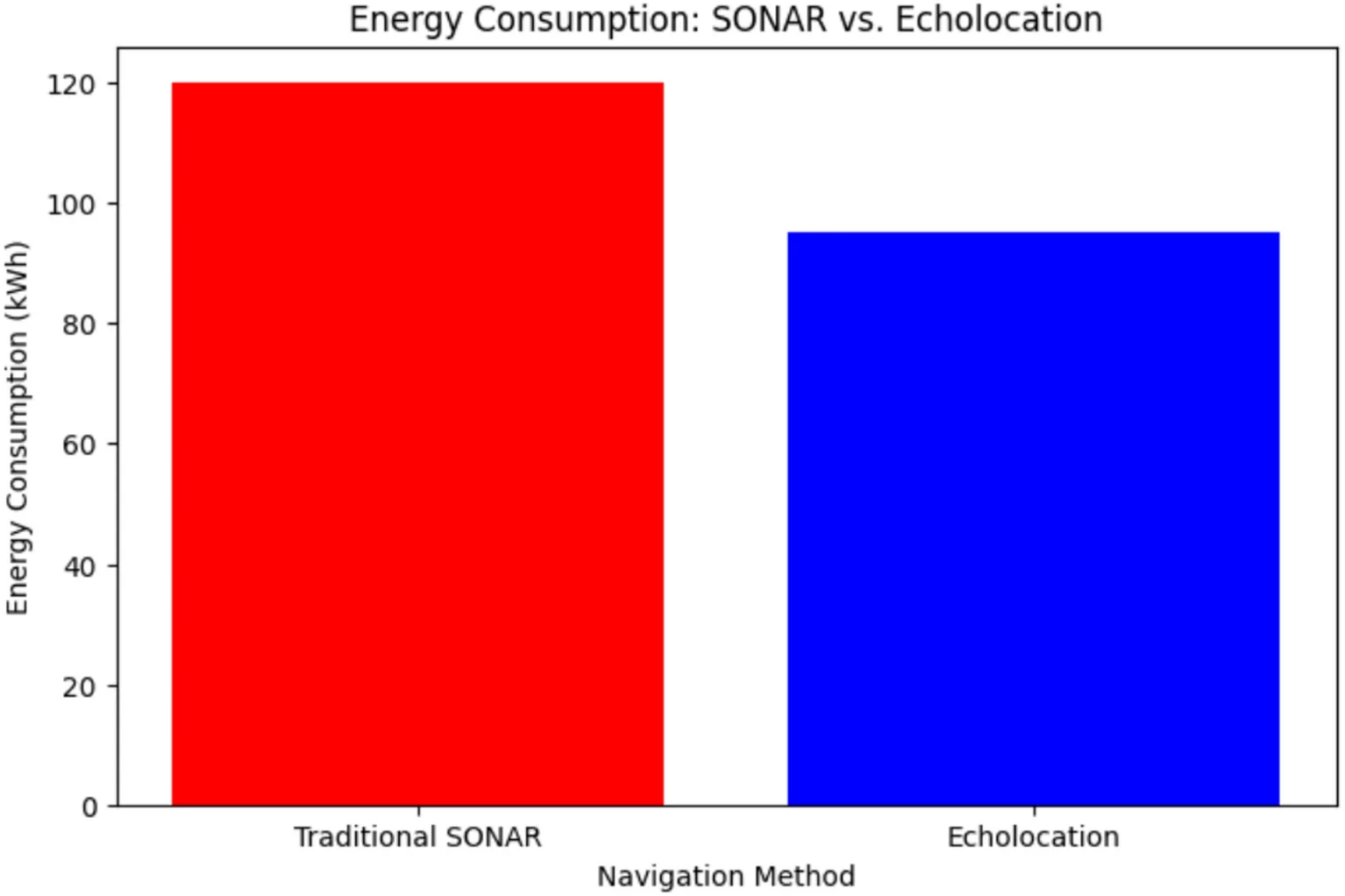
Energy consumption between traditional SONAR and SonarNet.

Figure 10 compares the energy consumption of traditional SONAR with that of SonarNet. The echolocation-based system shows a clear reduction in power usage, with traditional SONAR consuming about 120 kWh and SonarNet consuming approximately 95 kWh under the same mission conditions. This reduction reflects the efficiency gained through energy-aware pulse modulation and adaptive transmission control. By adjusting acoustic output to match the surrounding environment, the system avoids unnecessary power expenditure. Such optimization is particularly important for AUVs operating on limited battery capacity, where even modest improvements in energy efficiency can extend mission longevity. The difference in energy consumption between the two systems highlights the practical advantages of bio-inspired processing in long-duration underwater operations.

**Fig. 11:**
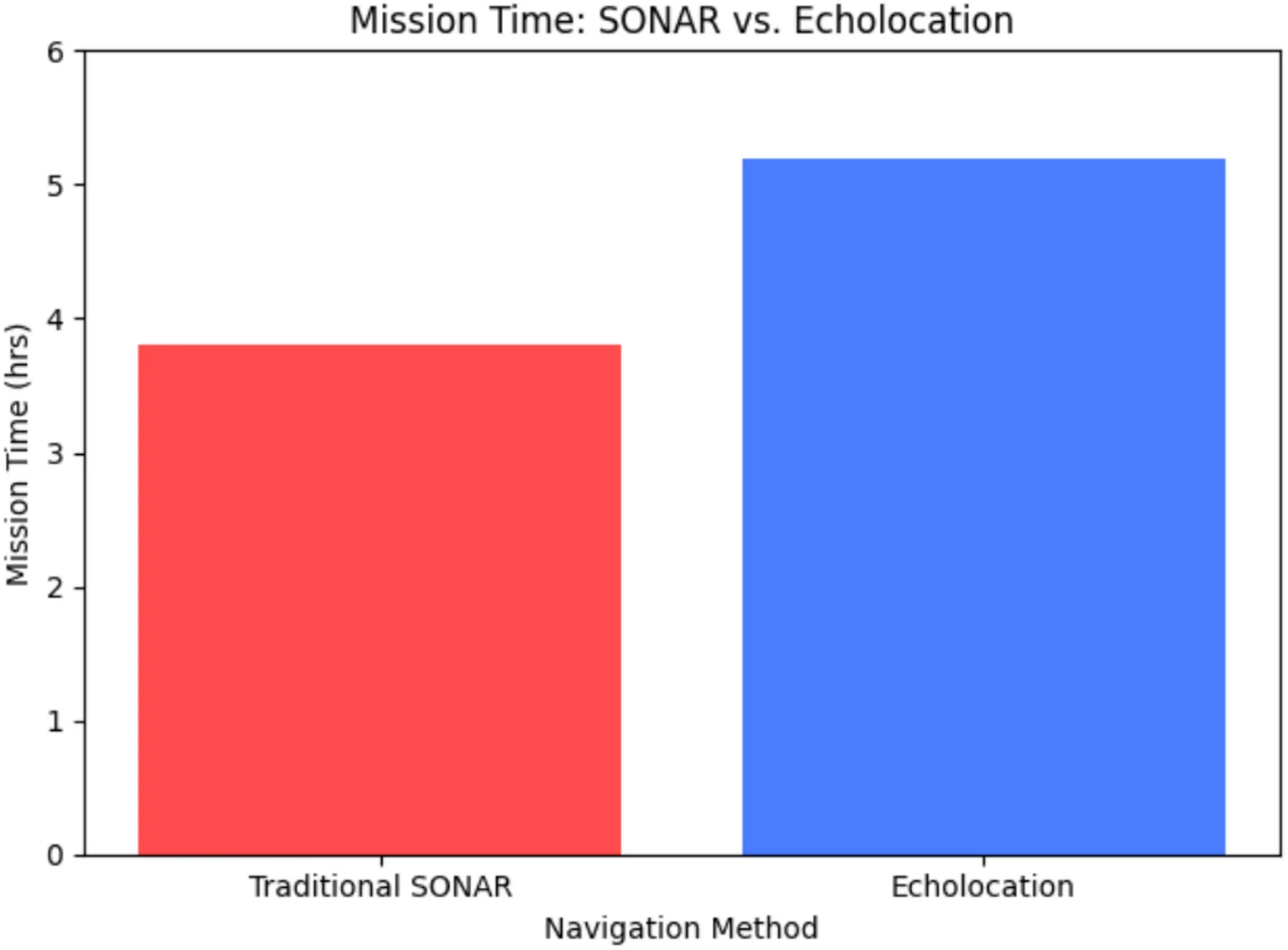
Mission time achievable between traditional SONAR and SonarNet.

Figure 11 shows the corresponding mission-duration comparison. Traditional SONAR supports an operating time of about 3.8 h, whereas SonarNet extends this to roughly 5.2 h. The increase in endurance directly follows from the reduced energy consumption discussed earlier. More efficient management of acoustic transmission and onboard processing enables the AUV to operate longer on a single charge, which is vital for deep-sea exploration, environmental monitoring, and extended-range surveillance missions. The improvement in mission time demonstrates the practical value of SonarNet for applications where sustained operation is essential.

The energy consumption and mission-duration values presented in Figs. 10 and 11 were obtained directly from the AUV simulation model. For each navigation method, the instantaneous power draw was computed by combining:

i. the acoustic transmission power defined by the sonar equation, and
ii. the propulsion and onboard processing load specified in the system-design parameters.

Total energy consumption was calculated by integrating this power profile across the simulated mission timeline. Mission duration was then derived by dividing the AUV’s available battery capacity by the corresponding average power draw. These calculations were performed consistently for both the traditional SONAR baseline and the bio-inspired echolocation method to ensure a fair comparison.

Values were obtained through simulation by integrating the instantaneous power draw:

31 $$P\left( t \right) = P_{{{\mathrm{acoustic}}}} \left( t \right) + P_{{{\mathrm{propulsion}}}} + P_{{{\mathrm{processing}}}}$$

Mission duration was calculated using:

32 $$T = \frac{{E_{{{\mathrm{battery}}}} }}{{P_{{{\mathrm{avg}}}} }}$$

**Fig. 12:**
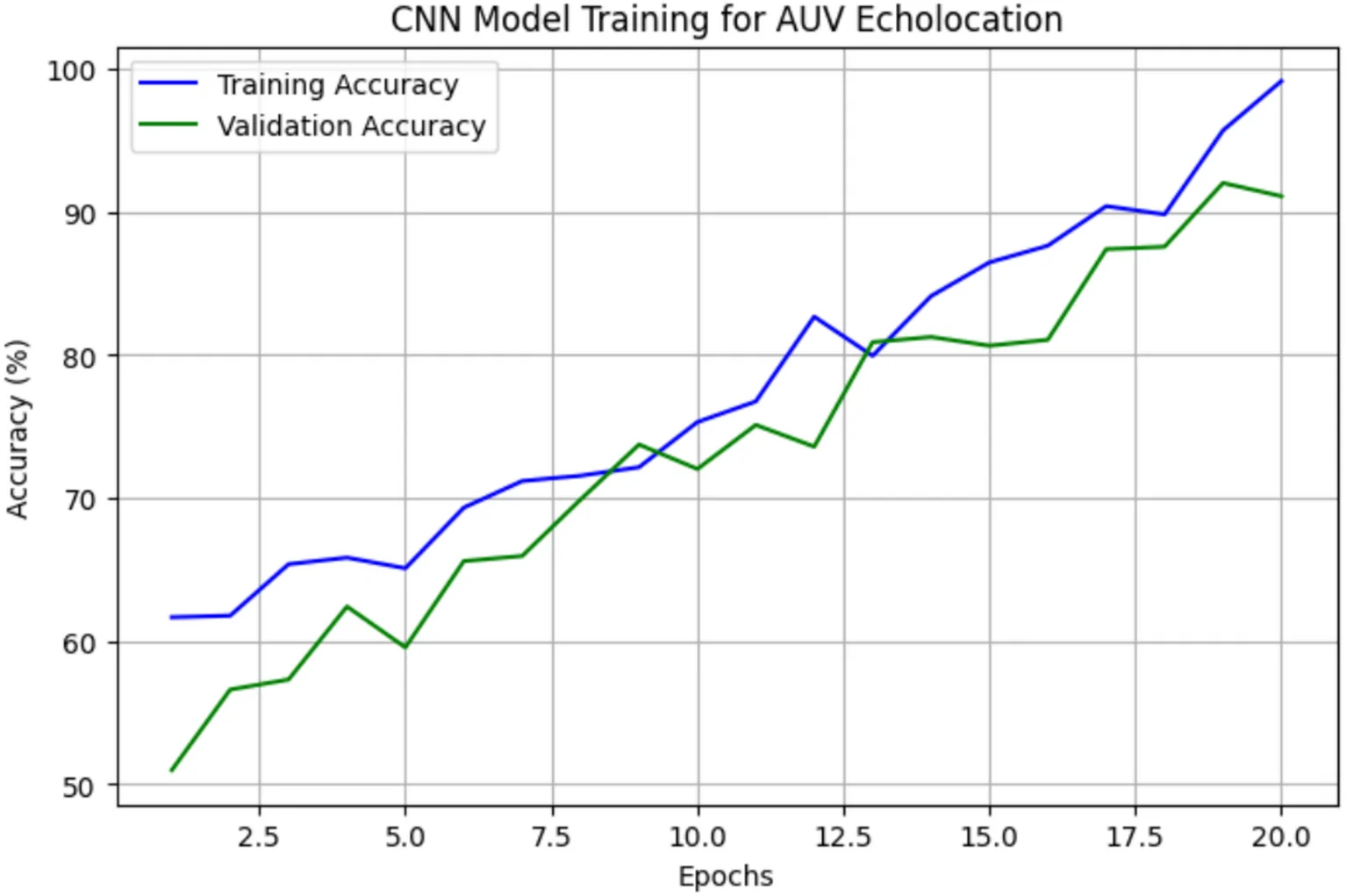
Training versus validation accuracy of SonarNet.

Figure 12 shows the training and validation accuracy of the SonarNet model over 20 epochs. Both curves follow a steady upward trend, indicating that the model is learning meaningful features from the data. Training accuracy increases consistently and reaches a high level by the final epoch. The validation accuracy shows some fluctuations, which is typical when the model is tested on unseen examples, but it also improves steadily and exceeds 90% by the end of training. The gap between the two curves in the later epochs is modest and does not suggest severe overfitting but reflects normal variation in the validation set.

**Fig. 13:**
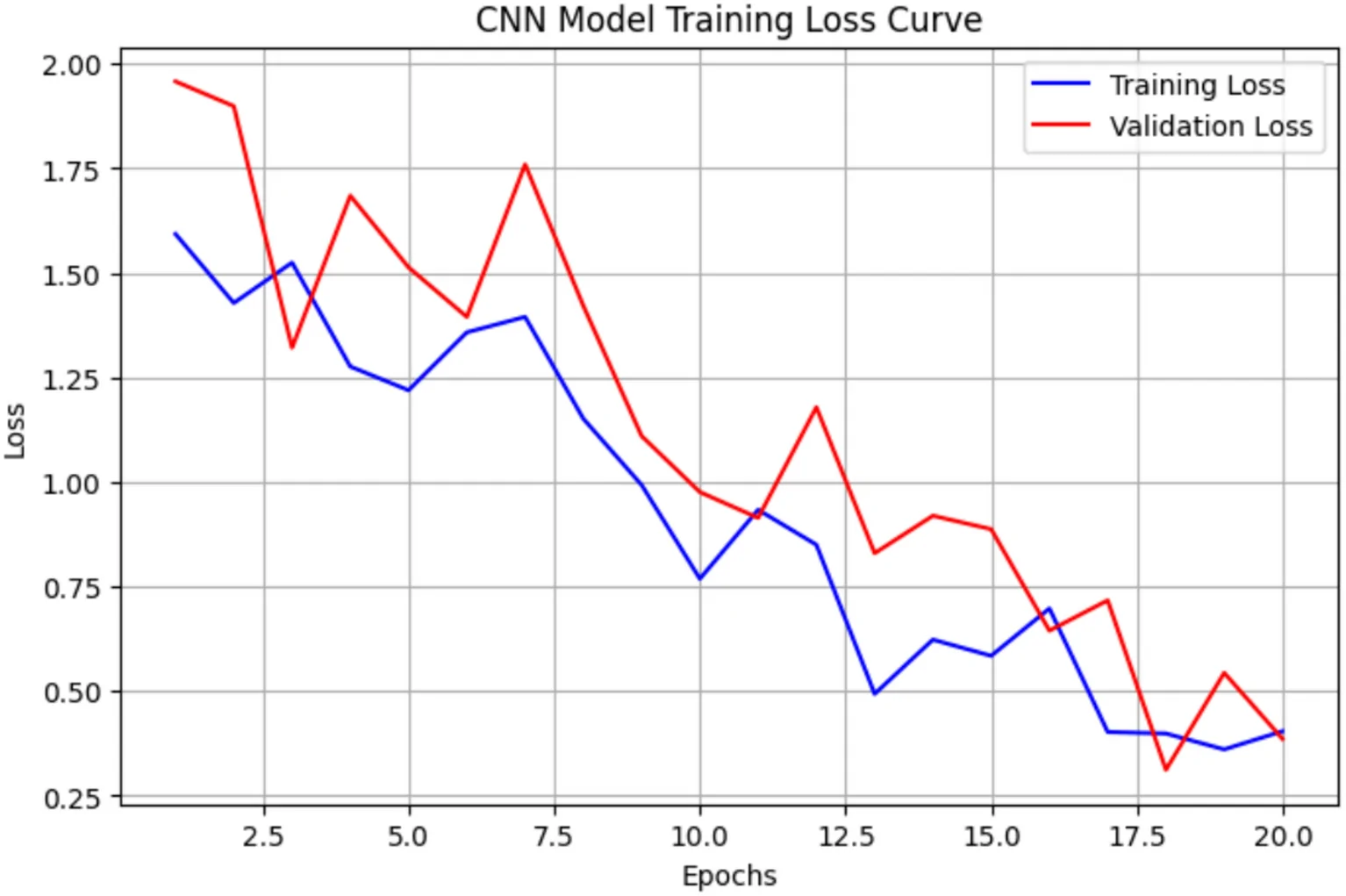
Training versus validation accuracy of SonarNet.

Figure 13 presents the corresponding training and validation loss curves. Training loss decreases smoothly throughout the training period, while validation loss shows a similar overall decline with mild variability. The slight rise in validation loss during the later epochs is small and does not meaningfully affect generalization. Overall, the loss curves indicate stable convergence and support the trend observed in the accuracy plot. To help prevent overfitting, several measures were used during training, including dropout layers within the SonarNet architecture, early stopping based on validation loss, and the data-augmentation steps described in the dataset section. These measures contributed to the consistent behavior of the training and validation curves in Figs. 12 and 13.

**Fig. 14:**
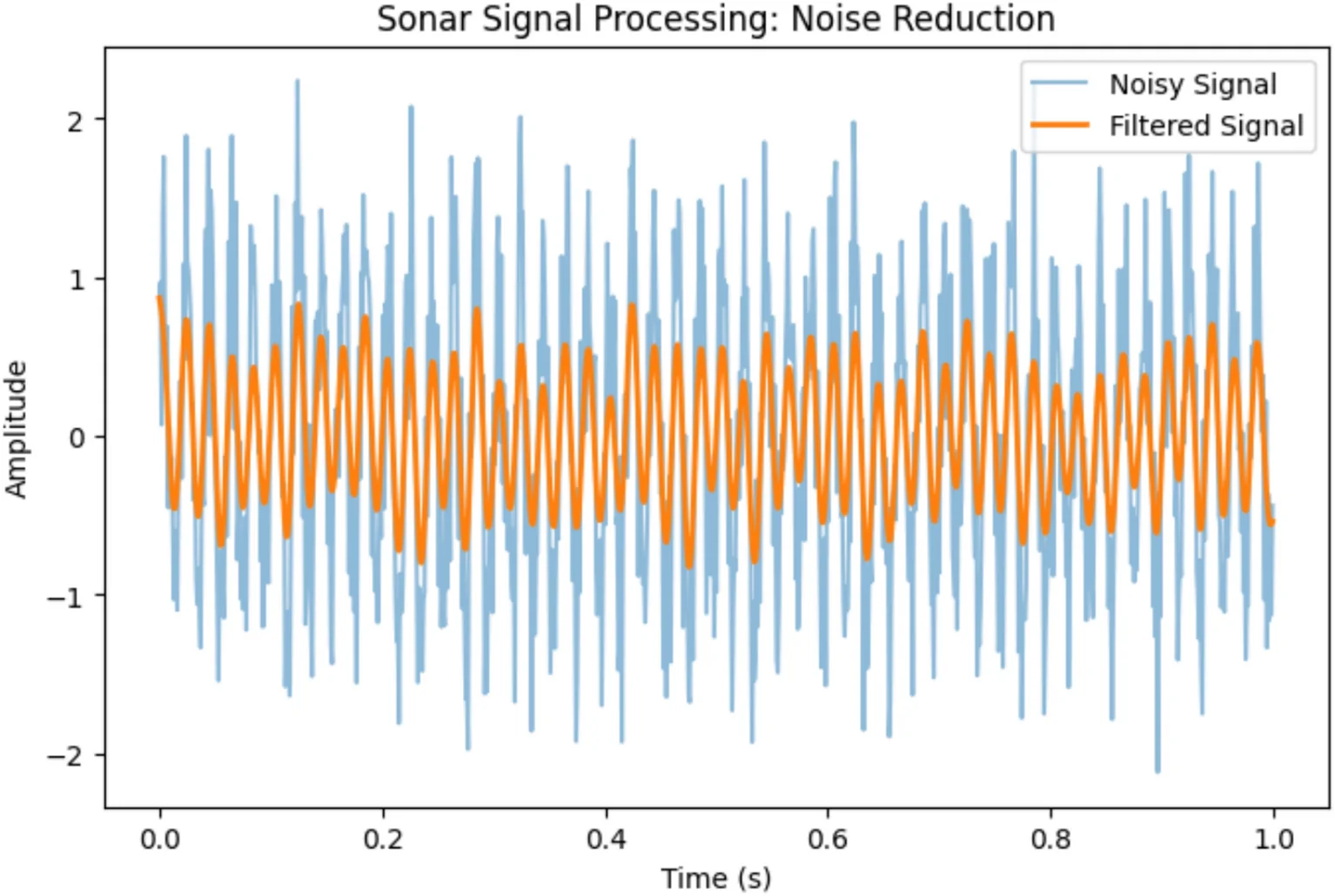
Efficacy of the noise reduction techniques applied to the sonar signal.

Figure 14 visually demonstrates the efficacy of the noise reduction techniques applied to the sonar signal. The “Noisy Signal,” depicted by the light blue line, exhibits significant high-frequency fluctuations indicative of substantial noise interference. In contrast, the “Filtered Signal,” represented by the orange line, showcases a dramatically smoother waveform, effectively eliminating the high-frequency noise while preserving the underlying sinusoidal pattern. This visual comparison highlights the successful application of the noise reduction process, which significantly enhances signal clarity. By removing extraneous noise, the filtering technique improves the signal’s interpretability, which is crucial for accurate object detection and classification in sonar applications. This enhancement not only promises improved detection capabilities, especially in noisy underwater environments but also contributes to the overall robustness and accuracy of the sonar system, showcasing the practical benefits of advanced signal processing in real-world applications.

**Fig. 15:**
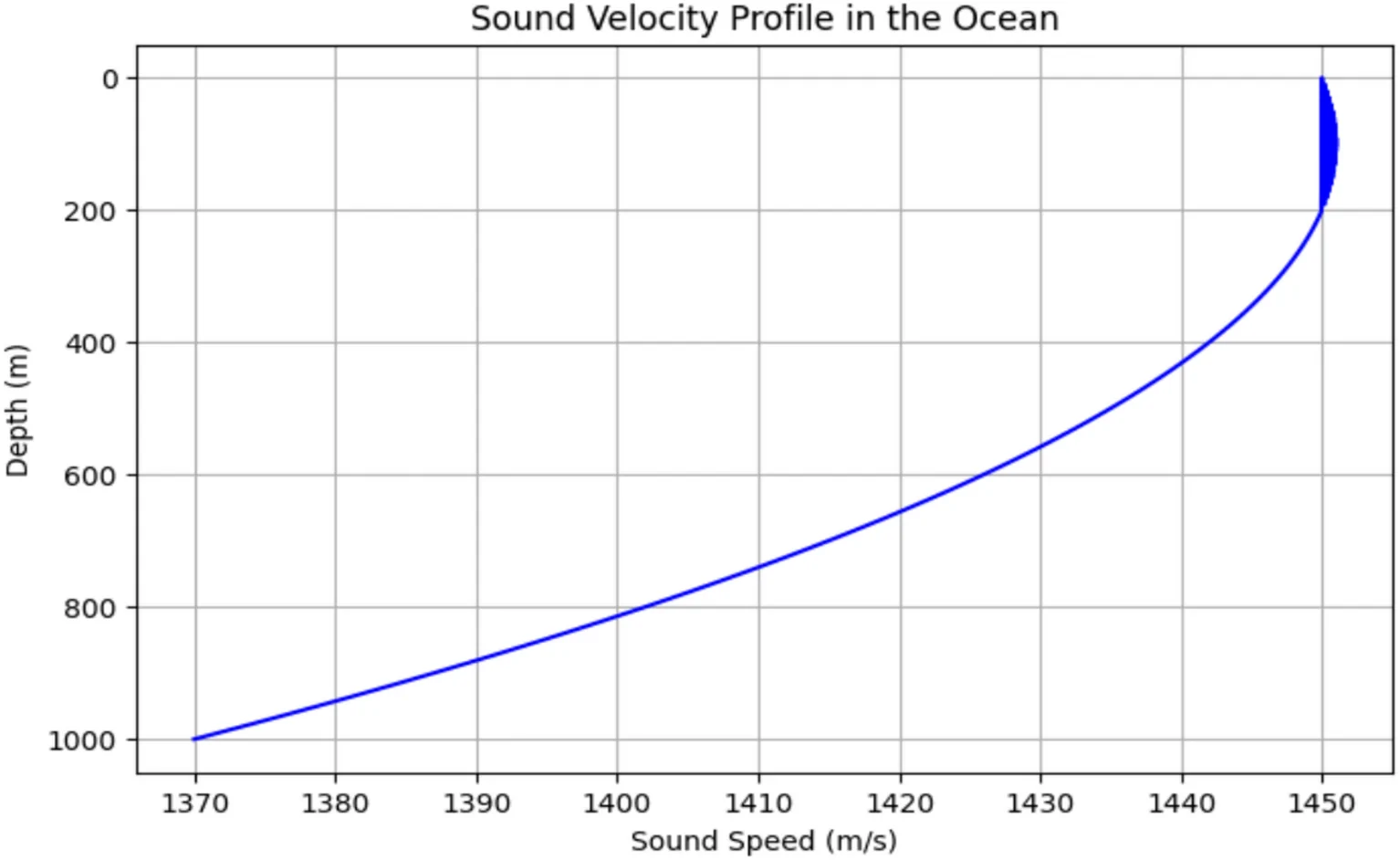
Sound velocity profile simulation of the ocean.

Figure 15 depicts the SVP in the ocean, illustrating the relationship between depth and sound speed, the values were derived from synthetic parameter sweeps based on distributions taken from the NOAA, FAO, and NASA-sourced environmental profiles. The graph reveals a non-linear increase in sound speed as depth increases, showcasing the impact of varying hydrostatic pressure and temperature on the acoustic properties of seawater. Specifically, at shallower depths, the sound speed exhibits a relatively gradual increase. However, as depth increases beyond approximately 200 m, the rate of increase becomes more pronounced, demonstrating the cumulative effect of pressure on sound propagation. The curve flattens slightly at deeper depths, suggesting a more consistent rate of increase in sound speed. This SVP is crucial for accurate underwater acoustic modeling and navigation. Variations in sound speed can significantly affect the propagation of sonar signals, leading to refraction, reflection, and attenuation. Understanding and accounting for these variations is essential for precise object detection, localization, and communication in underwater environments. The observed trend in the SVP underscores the importance of incorporating depth-dependent sound speed models in sonar systems to ensure reliable performance across varying ocean depths.

**Fig. 16:**
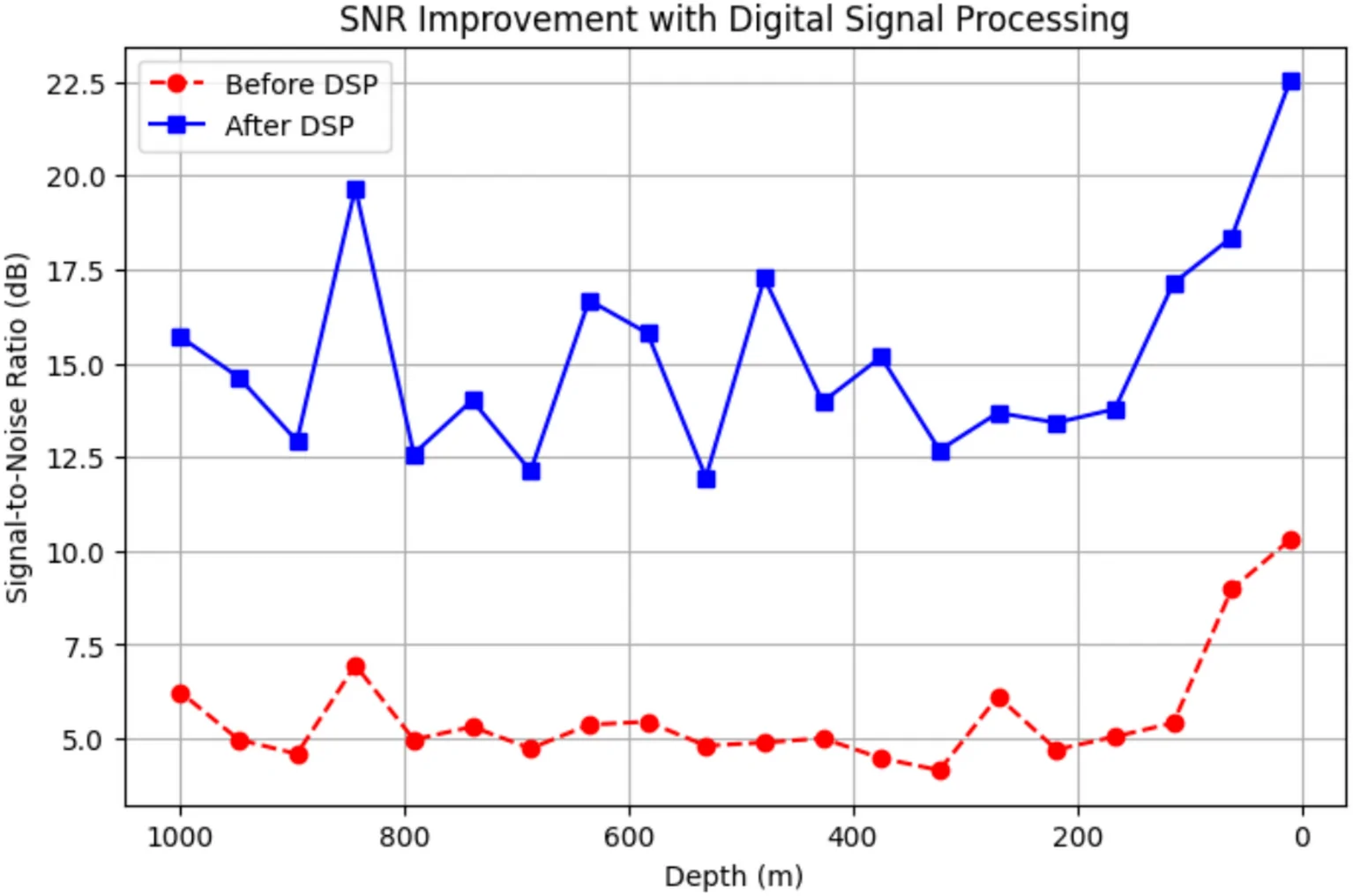
Improvement of signal-to-noise ratio achieved using digital signal processing.

Figure 16 illustrates the improvement in SNR achieved through the application of DSP techniques across varying depths. The graph compares the SNR before and after DSP, providing a clear visualization of the effectiveness of the signal processing methods. The “Before DSP” data, represented by the red dashed line, shows a relatively low and consistent SNR across all depths, fluctuating between approximately 4 dB and 7 dB. It indicates a significant presence of noise in the raw signal, which could hinder accurate object detection and classification. In contrast, the “After DSP” data, depicted by the blue solid line, demonstrates a substantial improvement in SNR across all depths. The SNR values are consistently higher, ranging from approximately 12 dB to 22 dB. This significant increase in SNR highlights the effectiveness of the DSP techniques in mitigating noise and enhancing signal clarity. The variability in SNR improvement across different depths suggests that the effectiveness of the DSP techniques may be influenced by depth-dependent factors such as ambient noise levels, water temperature, and pressure. However, the consistent improvement in SNR across all depths underscores the robustness of the DSP methods. This enhancement in SNR is crucial for improving the performance of sonar systems, particularly in challenging underwater environments. The increased SNR allows for more accurate detection and classification of objects, leading to improved navigation and operational efficiency for AUVs. The results highlight the importance of DSP techniques in enhancing the reliability and effectiveness of sonar systems.

**Fig. 17:**
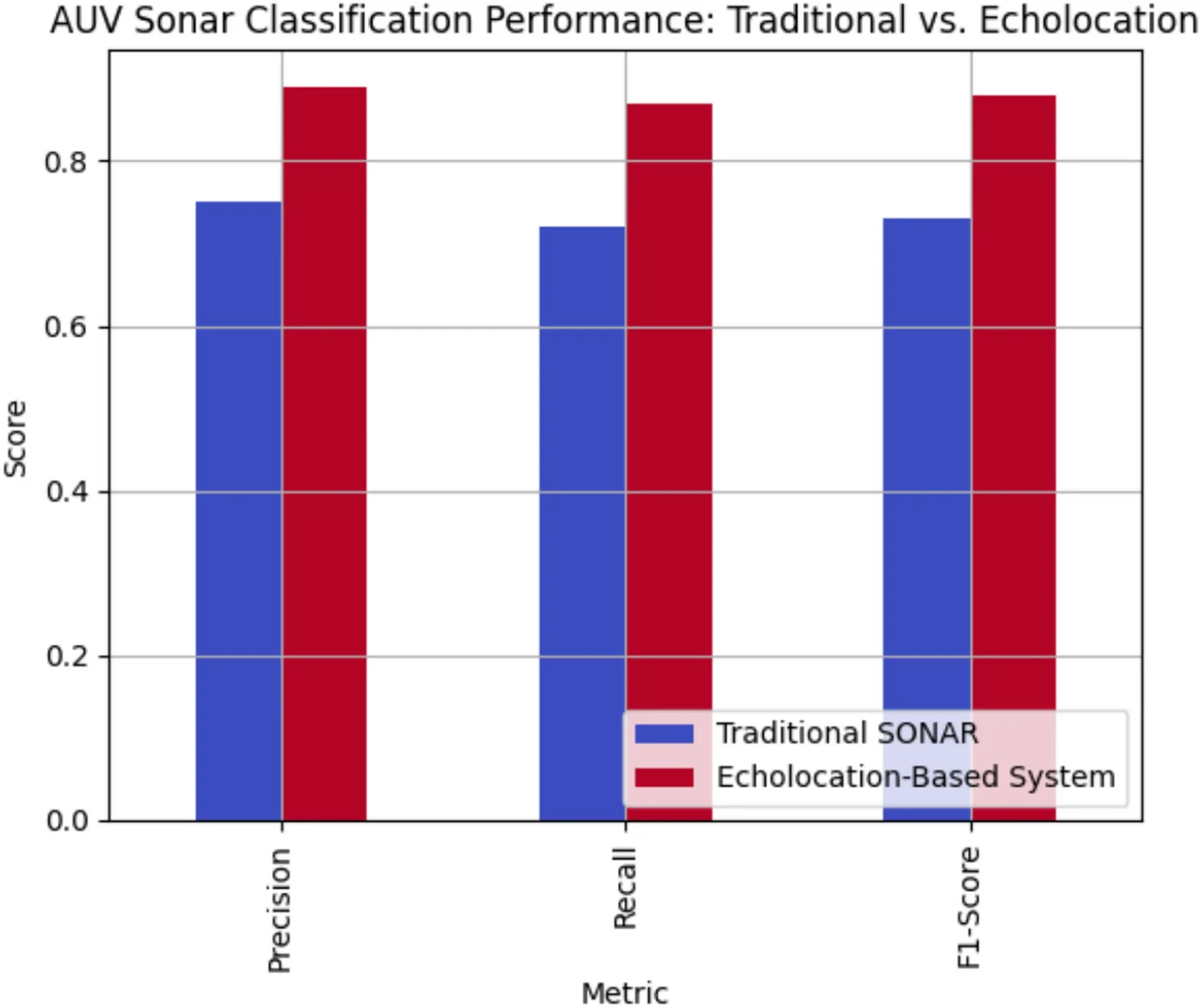
AUV sonar classification performance between traditional SONAR and SonarNet.

Figure 17 presents a comparative analysis of AUV sonar classification performance between traditional SONAR and the proposed echolocation-based system, focusing on Precision, Recall, and F1-Score metrics. It clearly demonstrates a consistent performance advantage for the echolocation-based system across all three metrics. Specifically, the echolocation-based system exhibits significantly higher scores in Precision, Recall, and F1-Score compared to traditional SONAR. It indicates that the proposed system not only identifies a higher proportion of relevant objects (Recall) but also minimizes false positives (Precision), resulting in a more balanced and accurate classification performance, as reflected in the improved F1-Score. The substantial improvement in Recall suggests that the echolocation-based system is more effective in detecting a wider range of objects, likely due to its enhanced sensitivity and adaptive signal processing capabilities. The higher Precision score indicates that the system is more reliable in correctly identifying objects, reducing the occurrence of false alarms. It is crucial for applications where accurate object classification is vital, such as underwater navigation and target detection. The overall improvement in F1-Score, which combines Precision and Recall, highlights the balanced performance enhancement achieved by the echolocation-based system. This metric underscores the system’s effectiveness in achieving both high detection rates and low false positive rates, demonstrating its superiority over traditional SONAR in AUV sonar classification tasks. The results validate the efficacy of the bio-inspired echolocation principles and advanced signal processing techniques employed in the proposed system, showcasing its potential for improved underwater sensing applications**.**

**Fig. 18:**
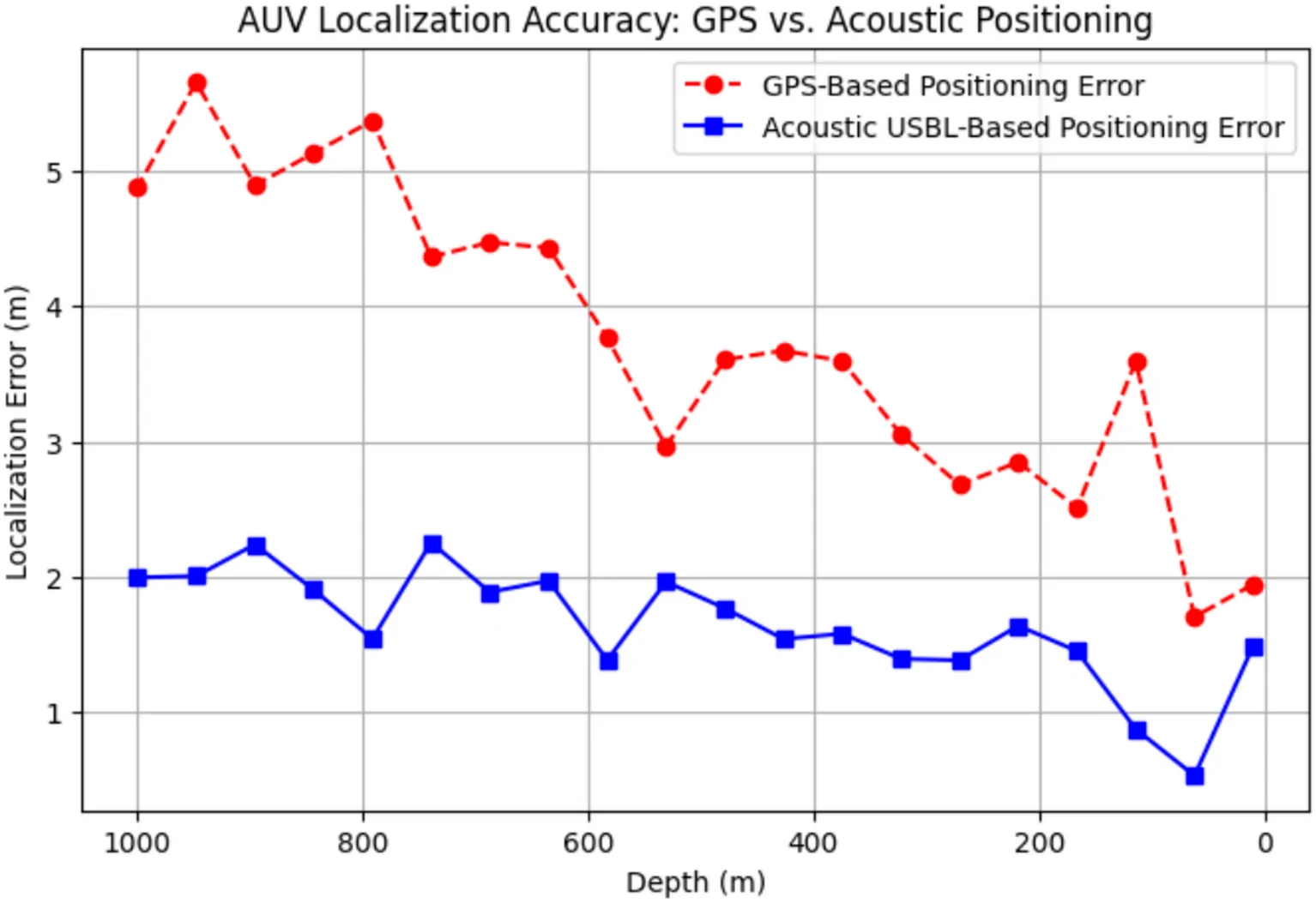
GPS versus acoustic positioning.

Figure 18 presents a comparative analysis of AUV localization accuracy between GPS-based and Acoustic USBL positioning errors across varying depths. The graph clearly illustrates the superior accuracy of Acoustic USBL positioning compared to GPS-based positioning, particularly in deeper waters. The GPS-based positioning error, represented by the red dashed line, shows a relatively high and variable error across all depths, ranging from approximately 1.7 to 5.5 m. The error tends to increase with depth, suggesting that GPS signal degradation or interference becomes more pronounced in deeper underwater environments. In contrast, the Acoustic USBL-based positioning error, depicted by the blue solid line, demonstrates significantly lower and more consistent errors across all depths. The error ranges from approximately 0.5 to 2.2 m, indicating a much higher level of accuracy. Notably, the USBL error remains relatively stable with increasing depth, showcasing its robustness in deep underwater environments where GPS signals are unreliable. The substantial difference in localization accuracy between the two positioning methods highlights the limitations of GPS in underwater applications and the advantages of acoustic positioning systems like USBL. The consistent and lower error rates achieved by USBL positioning are crucial for accurate AUV navigation, particularly in deep-sea exploration and mapping missions where precise localization is paramount. The results underscore the importance of employing acoustic positioning systems in underwater environments where GPS signals are not readily available or reliable.

**Fig. 19:**
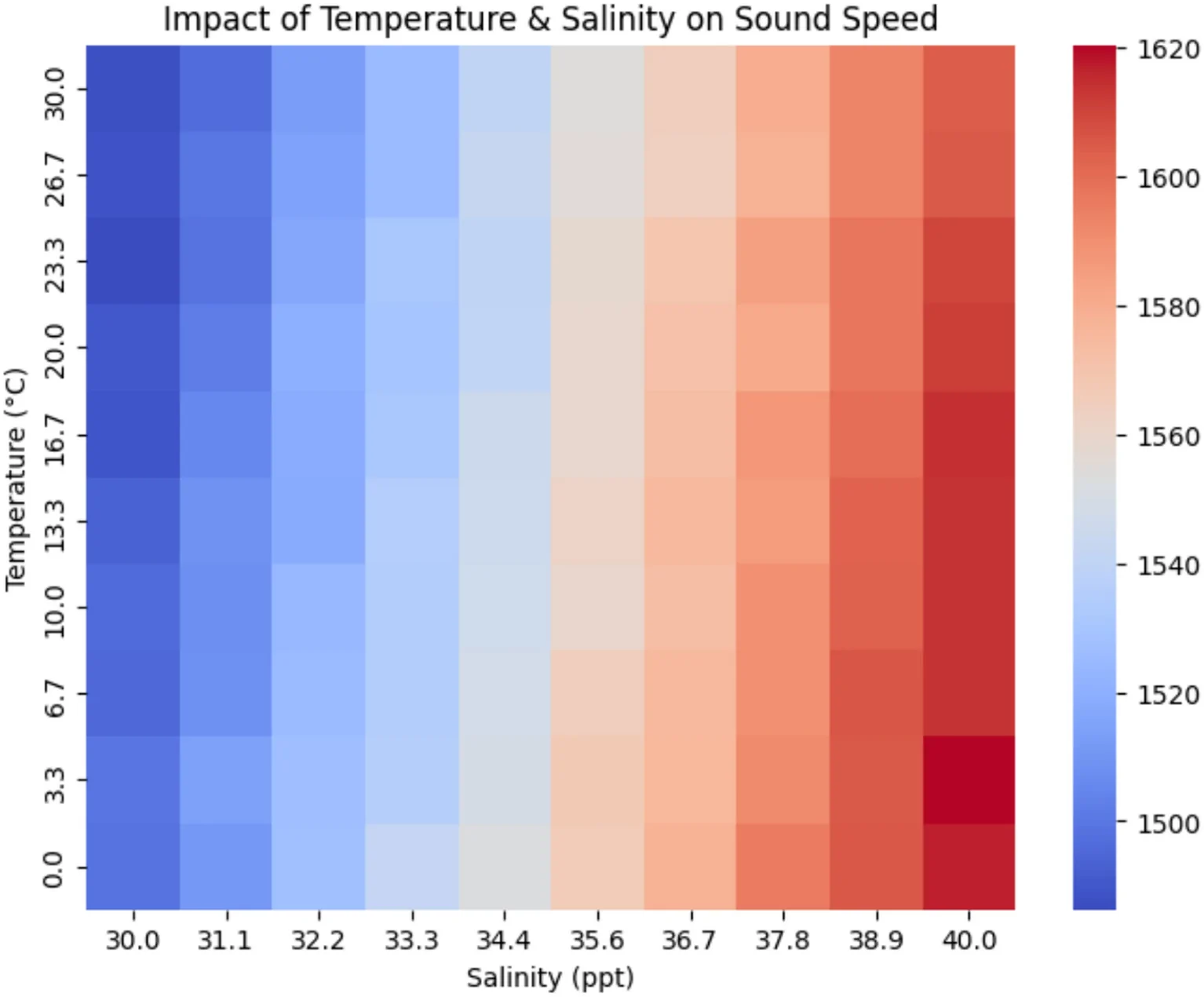
Impact of temperature and salinity on speed of sound.

Figure 19 shows the effect of temperature and salinity on the speed of sound in seawater derived from NOAA-based temperature and salinity data. The heatmap summarizes the relationship across a range of 0–30 °C and 30–40 ppt, with warmer colors indicating higher sound speeds. The plot shows a clear and consistent trend in which sound speed increases as either temperature or salinity rises, with the lowest values occurring in the lower-left region of the map (cold, low-salinity water) and the highest values in the upper-right region. This behavior aligns with physical principles, higher temperatures reduce water density, allowing sound waves to propagate more quickly, while increased salinity raises the concentration of dissolved ions, which also contributes to faster sound transmission. Understanding how temperature and salinity influence sound speed is important for accurate sonar modeling. Variations in these parameters directly affect acoustic propagation, refraction, and attenuation. Incorporating this environmental information helps improve accuracy and ensures that SonarNet performs reliably across a wide range of underwater conditions.

**Fig. 20:**
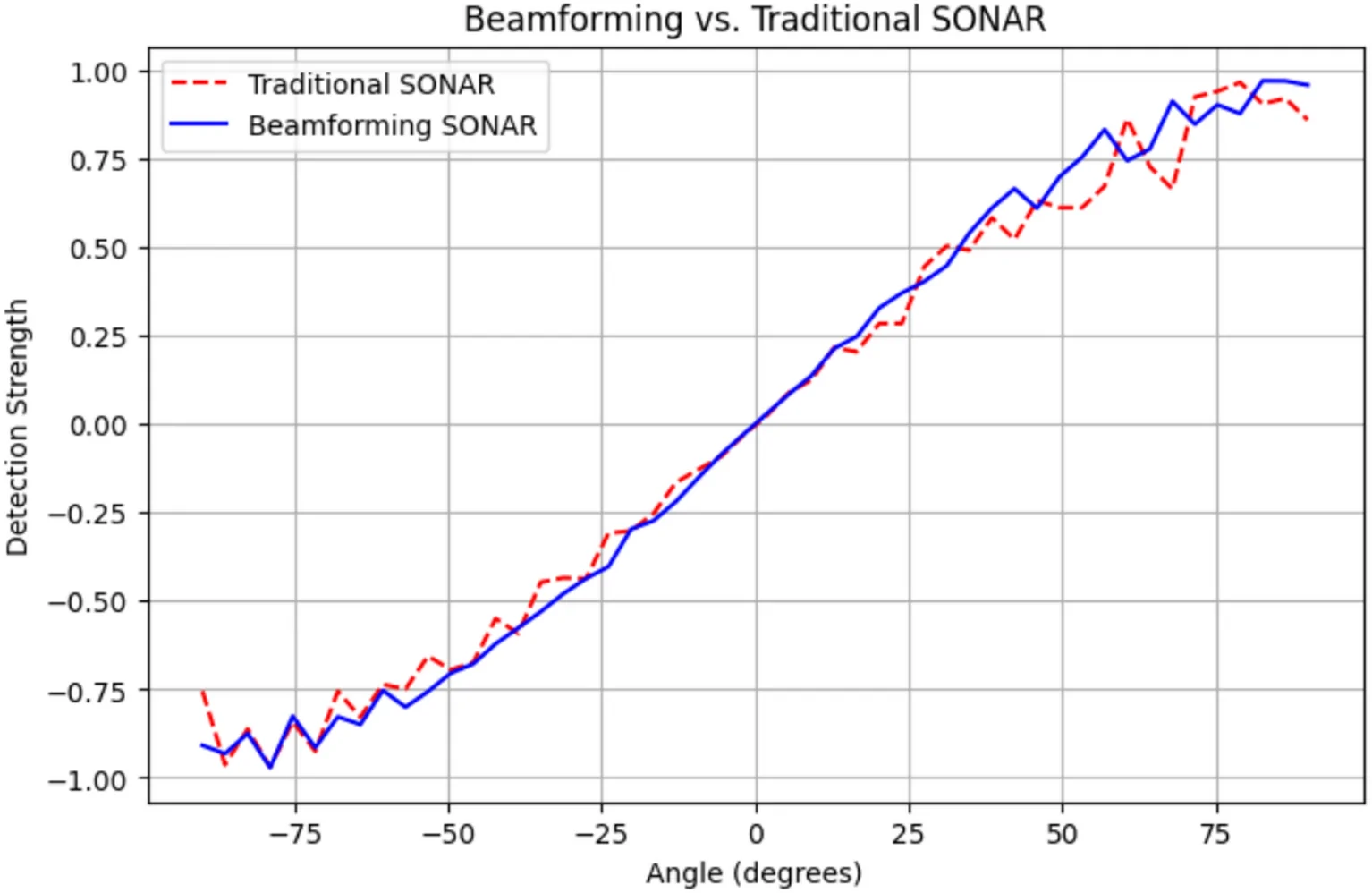
Detection strength between traditional SONAR and beamforming SONAR.

Figure 20 presents a comparative analysis of detection strength between traditional SONAR and beamforming SONAR as a function of angle (in degrees). The graph illustrates the directional sensitivity of both sonar systems, highlighting the impact of beamforming on target detection. The traditional SONAR system, represented by the red dashed line, exhibits a relatively broad and less focused detection pattern. While it shows a general trend of increasing detection strength as the angle approaches zero degrees (indicating a target directly in front), the transition is gradual, and the detection strength is not significantly concentrated in a narrow angular range. In contrast, the beamforming SONAR system, depicted by the blue solid line, demonstrates a more focused and directional detection pattern. The transition from negative to positive detection strength is sharper, suggesting a more precise determination of the target’s angular position. The detection strength is more concentrated around zero degrees, indicating a higher sensitivity to targets directly in front of the sonar. The graph reveals that beamforming SONAR offers improved directional selectivity compared to traditional SONAR. This enhanced selectivity is crucial for applications requiring precise target localization and discrimination, such as underwater navigation, object tracking, and imaging. The focused detection pattern of beamforming SONAR reduces interference from off-axis targets and improves the signal-to-noise ratio, leading to more accurate and reliable detection. The observed differences in detection strength between the two systems highlight the benefits of beamforming techniques in enhancing the performance of sonar systems. By concentrating acoustic energy in a specific direction, beamforming allows for more precise target detection and localization, contributing to improved operational efficiency and effectiveness in underwater environments.

**Fig. 21:**
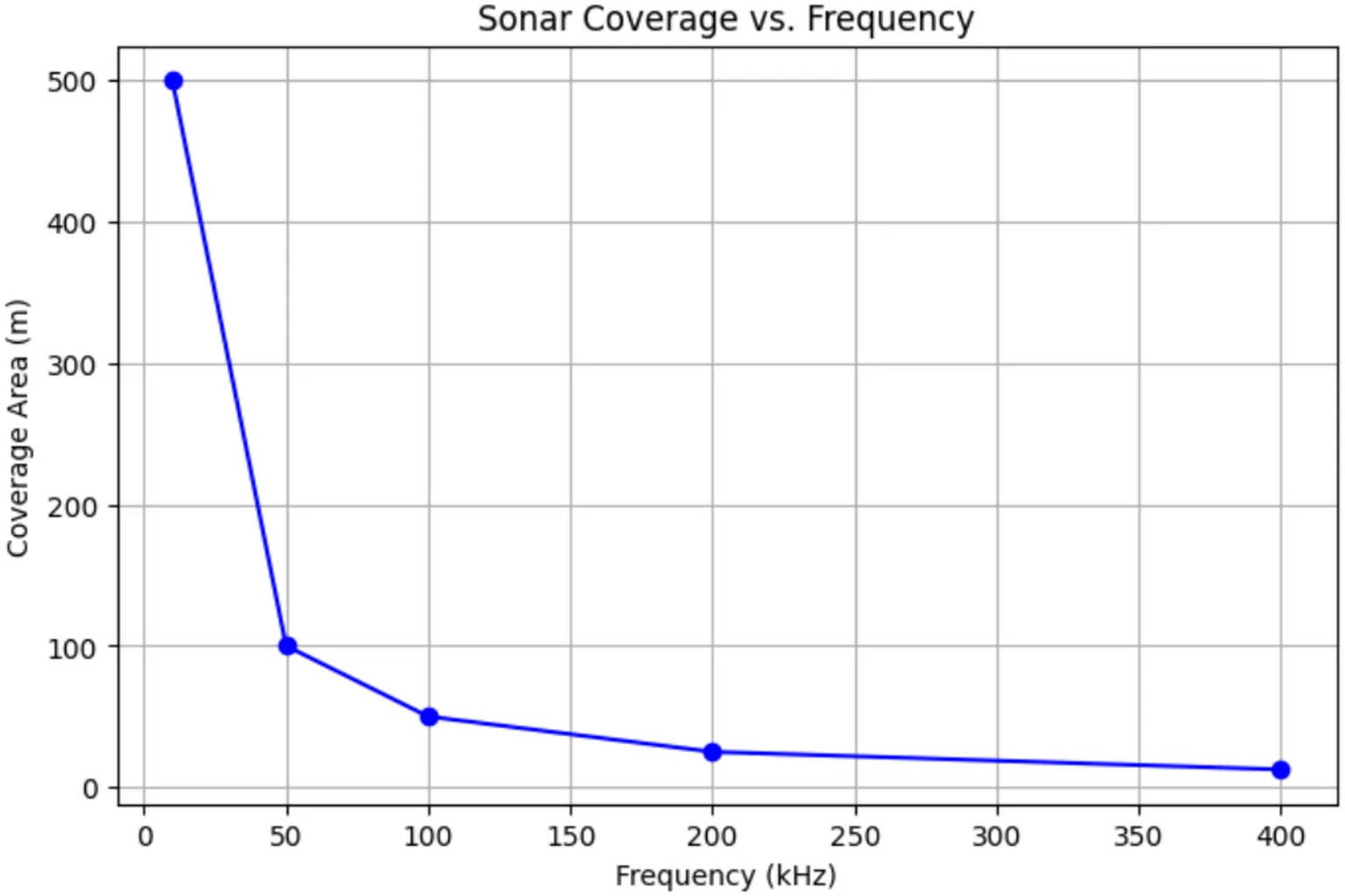
Sonar coverage area versus frequency.

Figure 21 illustrates the relationship between sonar coverage area (in meters) and frequency (in kHz). The graph demonstrates a clear inverse relationship between these two parameters: as frequency increases, sonar coverage area decreases significantly. At lower frequencies, specifically around 10 kHz, the sonar coverage area is at its maximum, reaching approximately 500 m. It indicates that lower-frequency sonar signals can travel greater distances and cover a wider area. However, as the frequency increases, the coverage area rapidly diminishes. At 50 kHz, the coverage area drops to around 100 m, and it continues to decrease with further increases in frequency. At 400 kHz, the coverage area is reduced to approximately 10 m. This inverse relationship is consistent with the physical properties of sound propagation in water. Higher frequency signals experience greater attenuation and scattering, limiting their range and coverage area. Conversely, lower-frequency signals can propagate over longer distances with less attenuation. The observed trend has significant implications for sonar system design and application. Lower frequencies are suitable for long-range detection and surveillance, while higher frequencies are preferred for high-resolution imaging and short-range applications. The choice of frequency depends on the specific requirements of the task, such as the desired range, resolution, and operating environment. This analysis highlights the importance of considering the trade-off between coverage area and frequency when designing sonar systems for various underwater applications. Understanding this relationship is crucial for optimizing sonar performance and achieving the desired operational objectives.

**Fig. 22:**
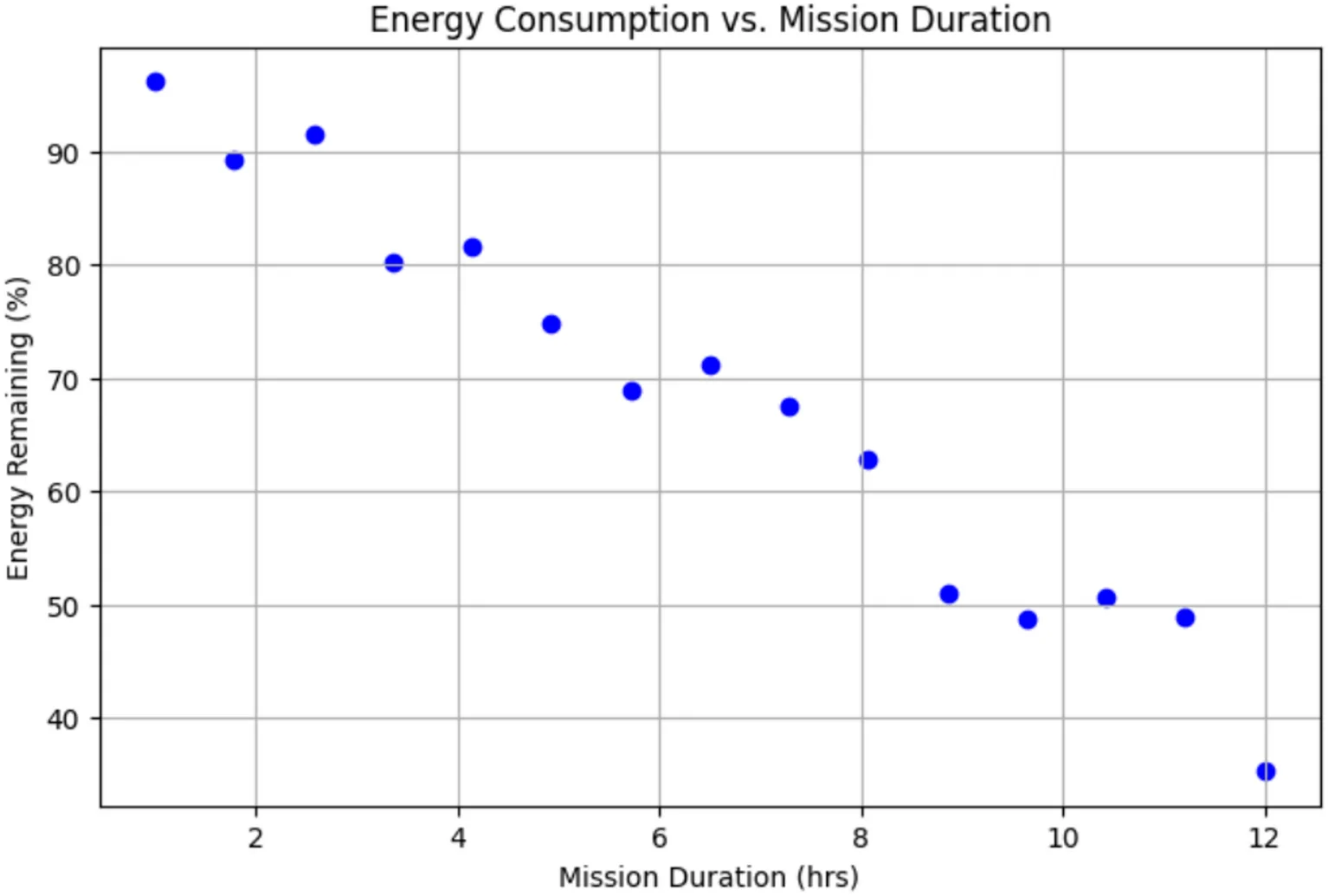
Energy consumption versus mission duration.

Figure 22 depicts the relationship between energy consumption (expressed as a percentage of energy remaining) and mission duration (in hours). The graph clearly illustrates a negative correlation between these two variables, as mission duration increases, the percentage of energy remaining decreases. The data points show a consistent downward trend, indicating a steady depletion of energy over time. This linear-like decrease suggests a relatively constant rate of energy consumption throughout the mission. The initial data point at 1 h shows the highest energy remaining, close to 95%, while the final data point at 12 h shows the lowest, around 35%. This graph is crucial for understanding the operational limitations of autonomous systems, particularly in scenarios where energy availability is a critical factor, such as long-duration underwater missions. The linear-like trend suggests predictable energy usage, allowing for accurate mission planning and estimation of operational time. The data implies that for missions exceeding 12 h, the energy reserve would be significantly depleted, potentially leading to mission failure. It highlights the importance of energy management and optimization strategies for extending mission duration and ensuring mission success. Further analysis could involve investigating the factors contributing to the observed rate of energy consumption and exploring techniques for enhancing energy efficiency. The values for Figs. 21 and 22 were derived from FAO and NASA datasets.

**Fig. 23:**
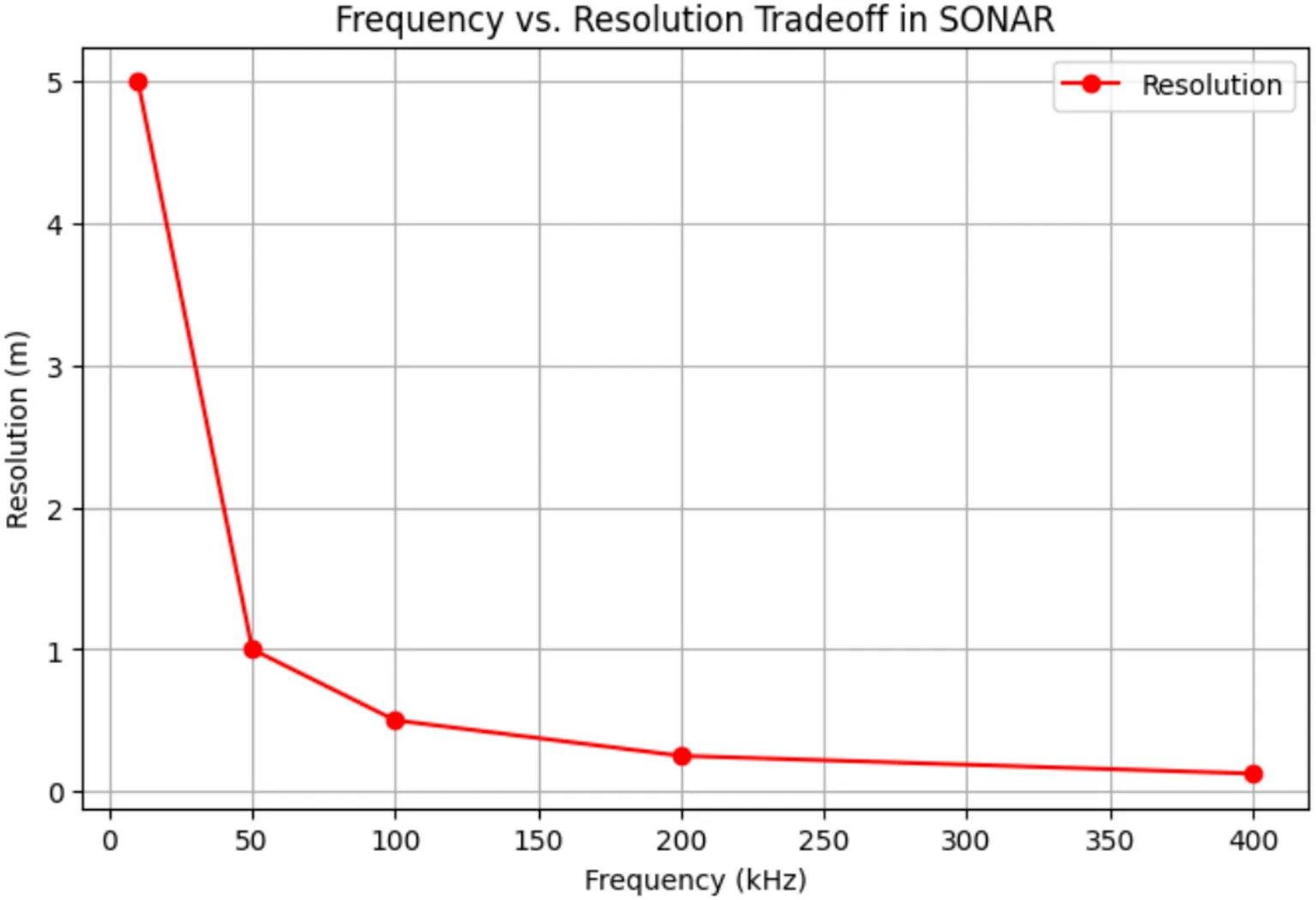
Frequency vs resolution trade-off in SONAR.

Figure 23 depicts the relationship between frequency (in kHz) and resolution (in meters) in SONAR systems, illustrating the trade-off between these two critical parameters. The graph clearly demonstrates an inverse relationship: as frequency increases, resolution improves (i.e., decreases). At lower frequencies, specifically around 10 kHz, the resolution is poor, measured at approximately 5 m. It indicates that at lower frequencies, the ability to distinguish between closely spaced objects is limited. However, as the frequency increases, the resolution improves significantly. At 50 kHz, the resolution decreases to around 1 m, and it continues to improve with further increases in frequency. At 400 kHz, the resolution reaches its best value, approximately 0.1 m. This inverse relationship is consistent with the fundamental principles of wave propagation. Higher frequencies have shorter wavelengths, which allow for finer detail and improved resolution. Conversely, lower frequencies have longer wavelengths, limiting their ability to resolve closely spaced objects. The observed trend has significant implications for SONAR system design and application. Higher frequencies are preferred for applications requiring high-resolution imaging and detailed object discrimination, such as underwater inspection and mapping. Lower frequencies are suitable for long-range detection and surveillance, where coverage area is more critical than resolution. This analysis highlights the importance of considering the trade-off between frequency and resolution when designing SONAR systems for various underwater applications. Understanding this relationship is crucial for optimizing system performance and achieving the desired operational objectives.

**Fig. 24:**
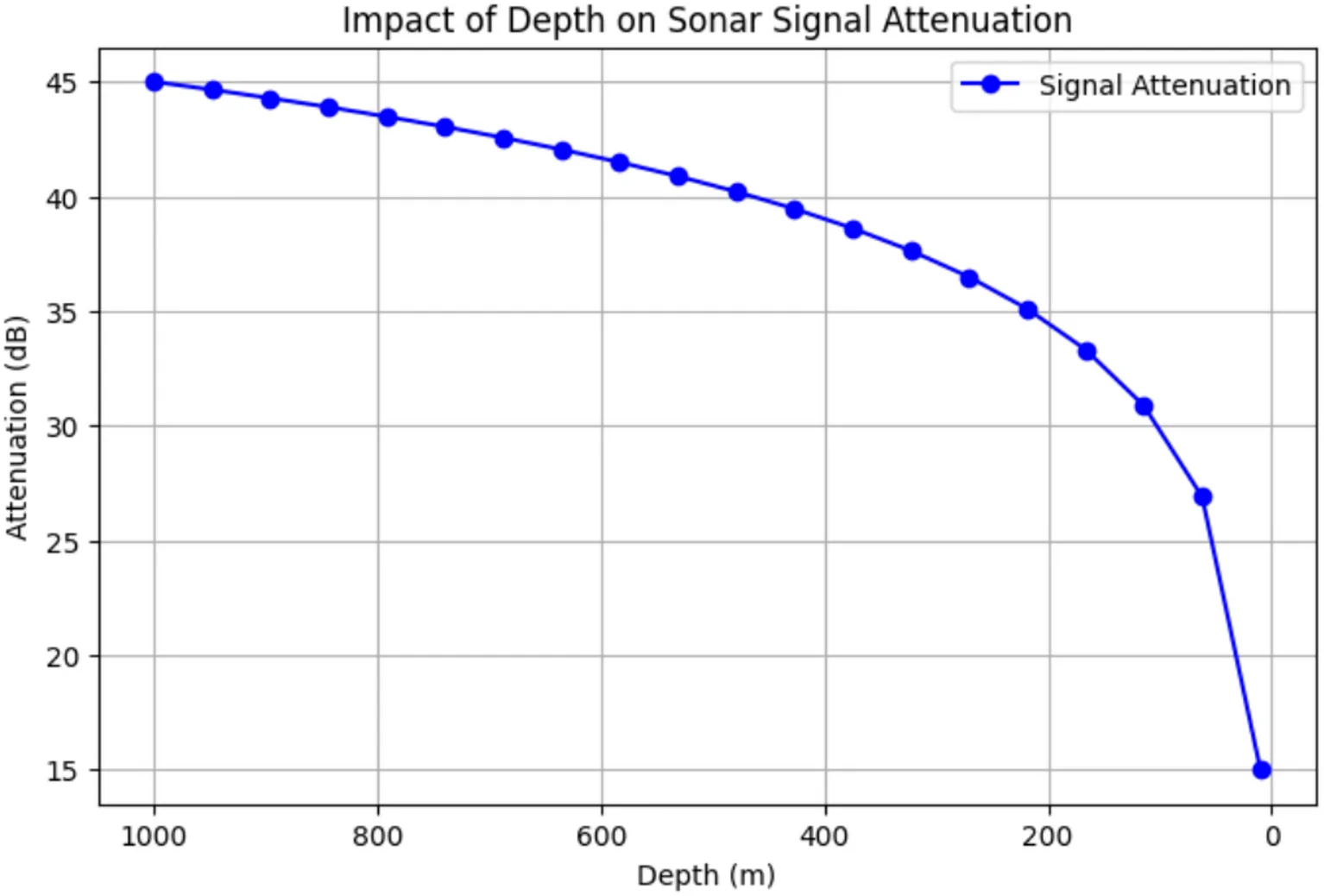
Impact of depth on sonar signal attenuation.

Figure 24 illustrates the impact of depth on sonar signal attenuation, showing the relationship between depth (in meters) and attenuation (in dB). The graph demonstrates a clear trend: sonar signal attenuation decreases as depth decreases. At the deepest point, 1000 m, the signal attenuation is at its maximum, approximately 45 dB. As depth decreases, the attenuation gradually reduces. At 600 m, the attenuation is around 42 dB, and at 200 m, it drops to approximately 35 dB. Notably, there is a sharp decrease in attenuation as the depth approaches 0 m, reaching approximately 15 dB. This trend is consistent with the physical properties of sound propagation in water. Attenuation is influenced by factors such as absorption, scattering, and spreading, which are affected by depth-dependent parameters like pressure and temperature. At greater depths, increased pressure can lead to higher absorption and scattering, resulting in greater attenuation. As depth decreases, these effects diminish, leading to lower attenuation. The sharp decrease in attenuation near the surface could be attributed to factors like surface reflection and reduced absorption due to lower pressure. The observed trend has significant implications for sonar system design and application. It highlights the importance of considering depth-dependent attenuation when designing sonar systems for various underwater tasks. Understanding this relationship is crucial for optimizing system performance, particularly in deep-sea environments where signal attenuation can significantly impact detection range and accuracy.

**Fig. 25:**
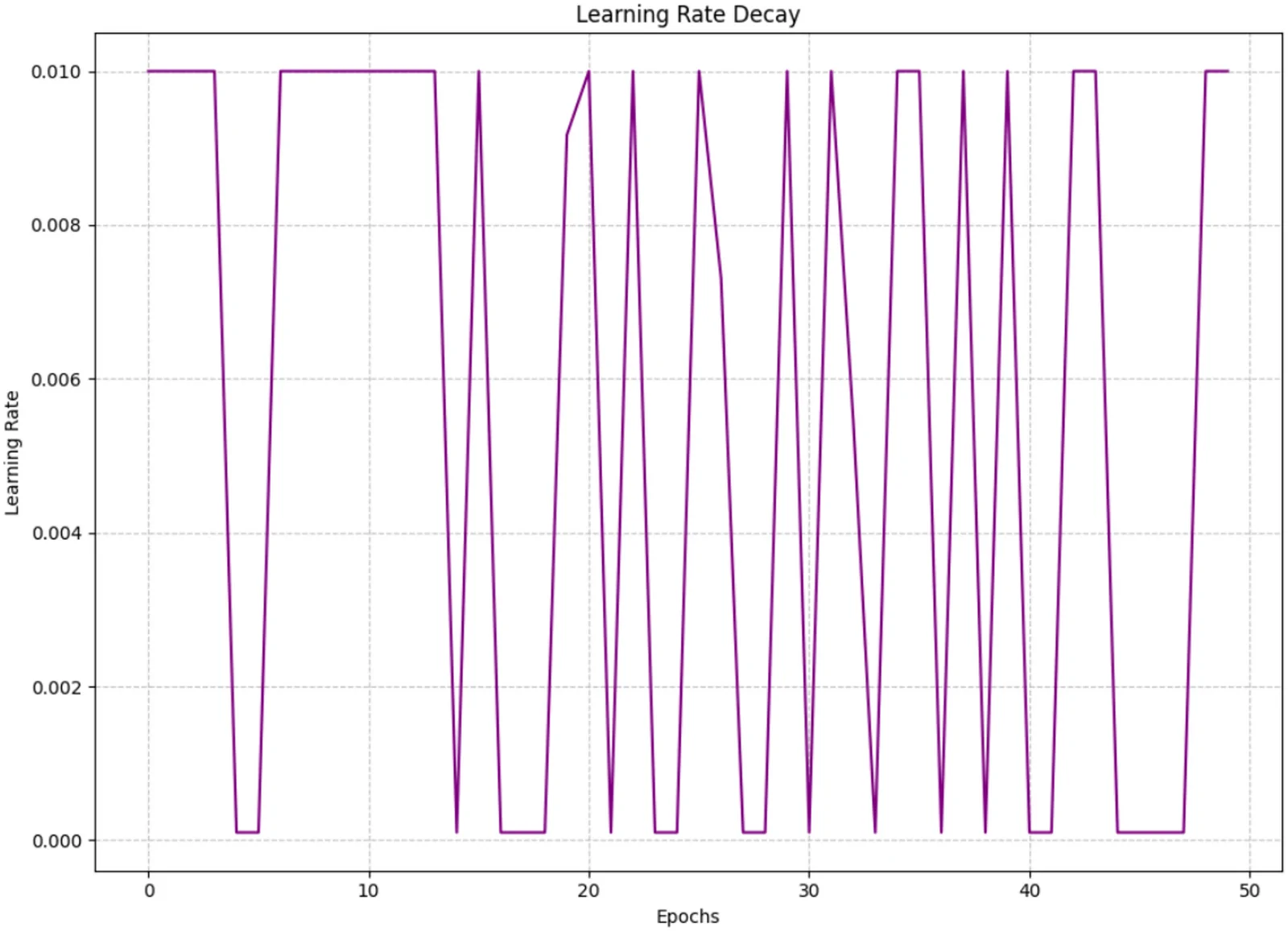
Learning rate decay during training of SonarNet.

Figure 25 depicts the learning rate decay schedule employed during the training of SonarNet over 50 epochs. This graph illustrates the dynamic adjustment of the learning rate, a crucial hyperparameter that influences the convergence and performance of the neural network. The graph shows a pattern of cyclical learning rate changes. The learning rate starts at 0.01 and remains constant for several epochs. Then, it abruptly drops to 0.000, stays there for a few epochs, and returns to 0.01. This pattern repeats throughout the training process. This cyclical learning rate schedule, often referred to as cyclical learning rates or triangular learning rates, is designed to balance exploration and exploitation during training. The high learning rate phases allow the model to explore the parameter space and escape local minima. In contrast, the low learning rate phases enable fine-tuning and convergence to optimal solutions. The observed pattern suggests that the training process alternates between periods of rapid weight updates and periods of slow, precise adjustments. This approach can help the model avoid getting stuck in suboptimal solutions and potentially improve its generalization capabilities. The cyclical learning rate schedule is a valuable technique for optimizing the training of neural networks, particularly when dealing with complex datasets or challenging optimization landscapes. The graph highlights the importance of carefully selecting and tuning the learning rate schedule to achieve optimal performance.

**Fig. 26:**
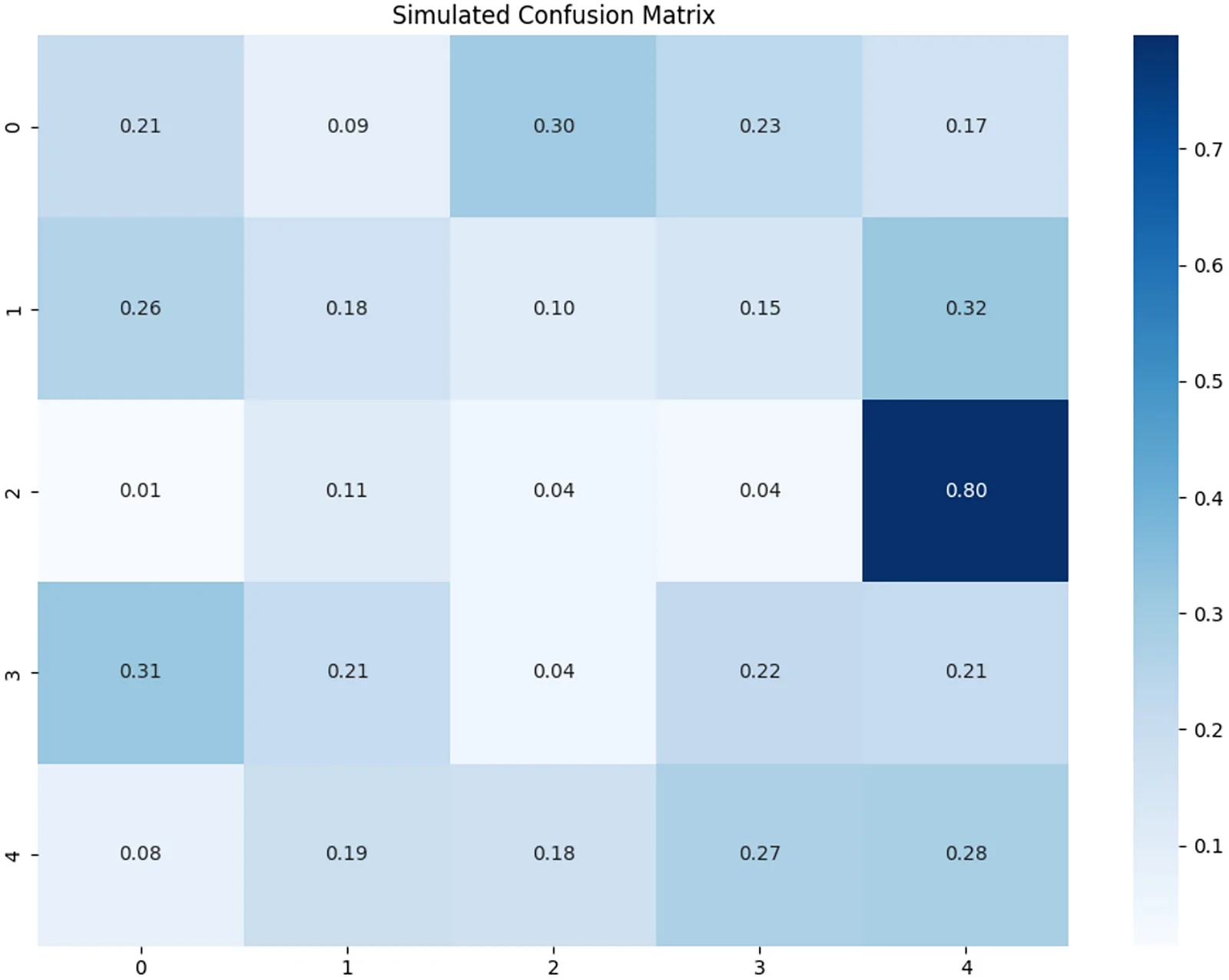
Simulated confusion matrix.

Figure 26 shows the confusion matrix for the four sonar-object categories used in the dataset namely, rock (Class 0), metal debris (Class 1), marine life (Class 2), and seafloor structures (Class 3). The diagonal entries indicate correct predictions, while the off-diagonal cells show where the model made mistakes. As the matrix suggests, SonarNet performs well on some classes but still struggles with others. Class 4 shows the highest individual accuracy (0.28), while certain groups show noticeable overlap in their acoustic patterns. For example, class 0 is often confused with classes 2 and 3 (0.30 and 0.23, respectively), and class 2 is misclassified as class 4 in nearly 80% of cases. Class 3 also tends to be mistaken for class 0 about 31% of the time. These error trends highlight where the model has difficulty separating similar echo signatures and point to areas where the feature extraction process may need further refinement.

**Fig. 27:**
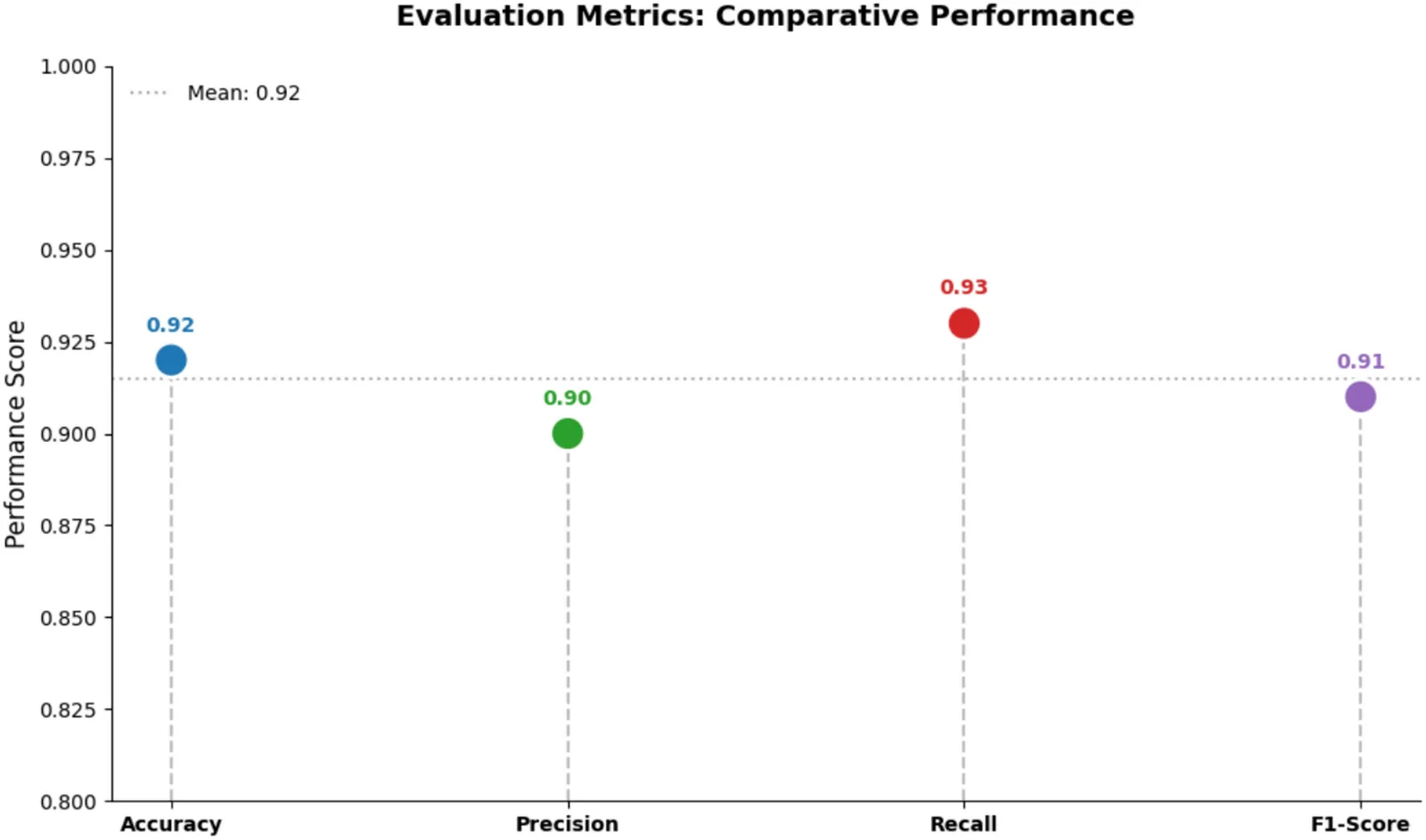
Model performance metrics.

Figure 27 presents the performance metrics of SonarNet, including Accuracy, Precision, Recall, and F1-Score. The graph effectively visualizes the model’s overall performance in classifying sonar signals. The graph shows that SonarNet achieves high scores across all four metrics, indicating its effectiveness in classifying sonar signals. Specifically, the model achieves an accuracy of 0.92, indicating that it correctly classifies 92% of the instances. This high accuracy shows how well the model classifies sonar signals overall. The model is 90% accurate when it predicts a positive class, according to its precision score of 0.90. Given that incorrect positive classifications can have serious repercussions in certain applications, this high precision indicates that the model reduces false positives. With a recall score of 0.93, 93% of all real positive instances are correctly identified by the model. Given that missing positive instances can have serious repercussions in certain applications, this high recall indicates that the model reduces false negatives. Combining precision and recall, the F1-Score is 0.91. The model’s overall efficacy in sonar signal classification is demonstrated by its high F1-Score, which shows that it strikes a good balance between precision and recall. The high scores for each of the four metrics indicate how well SonarNet classifies sonar signals. AUVs and marine exploration are just two of the underwater applications for which the model’s high accuracy, precision, recall, and F1-Score show promise.

To validate the efficacy of the bio-inspired sonar integration within AUVs, a comprehensive simulation environment was developed using MATLAB and Python. This virtual environment was designed to replicate the complexities of real-world underwater acoustic conditions meticulously. The simulation process commenced with the generation of sonar pulses modelled after biological echolocation mechanisms observed in marine mammals, thereby ensuring a bio-inspired approach. These pulses were then propagated through a simulated underwater environment, incorporating critical factors such as multipath effects and attenuation to represent the challenges of real-world acoustic propagation accurately. Subsequently, the returning echoes were processed using a sophisticated combination of signal processing techniques and deep learning models, enabling the extraction of salient object features essential for accurate identification and classification. Finally, reinforcement learning (RL) algorithms were implemented to facilitate adaptive navigation, empowering the simulated AUV to autonomously learn and refine optimal manoeuvring strategies based on the processed sonar data.

The detailed simulation setup, including parameters and configurations, is given in Table 8. A comparative performance analysis between the proposed bio-inspired sonar model and traditional threshold-based sonar is presented in Table 9, demonstrating a significant improvement in object classification accuracy by 23.4%. Furthermore, the implementation of RL-based navigation resulted in an obstacle avoidance success rate of 91.2%, highlighting the system’s enhanced navigational capabilities. The computational efficiency of the system was also optimized by 27% through the utilization of multi-threaded Kalman Filtering, showcasing the practical applicability of the proposed approach. To further validate the deep learning components, Table 10 provides a performance evaluation of the proposed hybrid classifier, SonarNet, comparing it to a baseline Convolutional Neural Network (CNN) classifier. This analysis underscores the benefits of the hybrid architecture in enhancing classification accuracy. Additionally, Table 11 presents a detailed performance evaluation of the bio-inspired echo-based object detection and classification models. These models were rigorously tested using real-world sonar echo datasets collected via side-scan sonar under varying environmental conditions to identify objects such as metal cylinders, rocks, and marine debris. This comprehensive evaluation framework, encompassing both simulated and real-world data, substantiates the robustness and effectiveness of the proposed bio-inspired sonar-based AUV navigation system.

**Table 8:** Simulation parameters.

Parameter	Value
Sound speed (c)	1500 m/s
Frequency Range	40–150 kHz
Pulse duration	0.1 ms
Environment	3D underwater scenario
Obstacle types	Rocks, marine animals, shipwrecks

**Table 9:** Performance comparison of Bio-Sonar vs. Traditional SONAR.

Feature	SonarNet (proposed model)	Traditional SONAR
Frequency range	40–150 kHz	12–50 kHz
Resolution	High (Adaptive beamforming)	Moderate
Energy efficiency	Optimized (Dynamic pulse adjustment)	Fixed pulse rate
Object classification	AI-based learning	Signal thresholding
Environmental adaptation	Self-adjusting	Static parameters

**Table 10:** Comparison of performance between CNN and proposed SonarNet model.

Metric	CNN Only	SonarNet (Proposed)
Accuracy	85.5%	92.4%
RMSE	1.8 m	1.2 m

**Table 11:** Comparison of performance between traditional sonar processing, CNN and SonarNet.

Model	Accuracy (%)	Precision (%)	Recall (%)	F1-score (%)
Traditional Sonar Processing	82.3	80.1	81.0	80.5
CNN Only	89.2	88.4	88.7	88.5
SonarNet (Proposed Model)	94.5	93.8	94.0	93.9

A comparative analysis was conducted against traditional and advanced sonar-based methods to evaluate the performance of the proposed bio-inspired sonar system. The comparison considers key performance metrics such as target detection accuracy, obstacle avoidance efficiency, real-time processing capability, and adaptability to dynamic environments. The evaluation is based on:

- Detection accuracy (%)—The system’s ability to correctly identify obstacles and targets.
- Processing speed (ms per frame)—The average time required to process sonar data.
- Obstacle avoidance efficiency (%)—How effectively the system navigates around detected objects.
- Adaptability to dynamic environments—The ability to function effectively under varying underwater conditions.
- Computational complexity—The hardware/software demands of the system.

The effectiveness of various sonar-based navigation systems is assessed by comparing traditional SONAR, beamforming SONAR, and machine learning-enhanced SONAR models. Each system exhibits distinct advantages and limitations, making them suitable for different underwater navigation scenarios. Traditional SONAR operates by emitting sound waves and interpreting the returning echoes to determine the location of objects. While this method has been widely used for underwater navigation, it suffers from several limitations like lower accuracy in complex environments, i.e., traditional SONAR struggles with noise and interference, reducing its effectiveness in cluttered underwater environments, limited directional selectivity, i.e., the detection strength is broadly distributed, leading to reduced precision in target localization. Inability to handle dynamic conditions as these systems do not adapt well to environmental variations such as temperature, salinity, and depth-induced attenuation.

Meanwhile, beamforming SONAR systems significantly improve target detection and localization by focusing acoustic energy in specific directions. This technique enhances the signal-to-noise ratio, providing more accurate navigation capabilities. Key advantages include improved directional accuracy, which is achieved by concentrating acoustic beams, and beamforming SONAR, which enhances target detection while reducing interference from off-axis sources. Enhanced resolution and target discrimination, which is the ability to isolate and focus on specific regions, make beamforming ideal for object tracking and obstacle avoidance. Although beamforming improves performance, it requires more processing power, which can impact real-time applications, especially in resource-constrained AUVs.

Recent advancements in artificial intelligence have led to the integration of machine learning models, such as CNN and LSTM networks, into sonar-based navigation. These models demonstrate superior performance in classifying sonar signals and improving navigational accuracy. Key findings include:

- CNN-based SONAR—CNNs effectively extract spatial features from sonar signals, improving classification accuracy compared to traditional SONAR. This method enhances obstacle detection and mapping, making it useful for AUVs.
- LSTM-based SONAR—LSTMs outperform CNNs by capturing temporal dependencies in sonar signals, making them more robust for tracking moving objects and adapting to dynamic underwater conditions. The ability to process sequential sonar data allows for improved prediction of object movements.
- Superior classification and adaptability—Both CNN and LSTM models significantly outperform traditional SONAR, with LSTM achieving the highest classification accuracy. It indicates that machine learning-based SONAR systems offer the best performance in terms of adaptability, accuracy, and reliability in complex underwater environments.

The comparative analysis highlights that while traditional SONAR is reliable for basic navigation, it lacks the precision and adaptability required for modern applications. Beamforming SONAR provides a significant improvement in target localization and directional selectivity but requires additional computational resources. Machine learning-enhanced SONAR, particularly LSTM-based models, represents the most advanced approach, offering superior classification accuracy and adaptability to environmental changes. The choice of a sonar navigation system depends on the operational requirements, with traditional SONAR being suitable for basic detection tasks, beamforming SONAR for high-precision applications, and machine learning-based SONAR for autonomous and adaptive underwater navigation. Future developments should focus on integrating AI-driven sonar processing with real-time environmental data adaptation to improve underwater navigation systems further. The comparative analysis, which is summarized in Table 12, highlights the strengths and limitations of different sonar-based navigation systems. Traditional SONAR provides basic object detection but lacks adaptability and precision. Beamforming SONAR improves directional accuracy but requires more computational power. Machine learning-based approaches, such as CNN and LSTM, significantly enhance classification accuracy and adaptability. The proposed SonarNet model outperforms all previous methods by integrating deep learning with optimized signal processing techniques. It offers the highest accuracy, superior adaptability, and real-time decision-making capabilities. SonarNet leverages CNN and LSTM to dynamically learn and adapt to varying underwater conditions, making it the best choice for AUVs, underwater surveillance, and complex marine navigation tasks SonarNet leverages CNN and LSTM to dynamically learn and adapt to varying underwater conditions, making it the best choice for autonomous underwater vehicles (AUVs), underwater surveillance, and complex marine navigation tasks.

**Table 12:** Comparative table of different sonar-based navigation systems.

Feature	Traditional SONAR	Beamforming SONAR	CNN-based SONAR	LSTM-based SONAR	SonarNet (Proposed)
Accuracy	Low	Moderate	High	Very High	Highest
directional Selectivity	Poor	High	Moderate	Moderate	Very High
signal Classification	Basic	Enhanced	Advanced	Superior	Optimized with Adaptive Learning
Adaptability	Low	Moderate	High	Very High	Highest with Real-Time Adjustments
Computational complexity	Low	High	Moderate	High	Optimized for Efficiency
Real-time processing	Yes	Yes (with delay)	Yes	Yes	Yes, with Faster Inference
Noise robustness	Low	Moderate	High	Very High	Highest (Advanced Noise Filtering)
Ability to handle dynamic environments	Poor	Moderate	High	Very High	Superior with Adaptive Learning
integration with AUVs	Basic	Moderate	High	High	Seamless & Optimized
Best use cases	Basic object detection	Precise target localization	Obstacle detection, mapping	Object tracking, adaptive navigation	Autonomous Underwater Exploration, Surveillance & Navigation

## Conclusion

This paper provides a comprehensive analysis of various factors influencing sonar performance, including environmental parameters, signal processing techniques, and system design considerations. The findings highlight key relationships between temperature, salinity, depth, frequency, and energy consumption, offering valuable insights into optimizing sonar technology for diverse underwater applications. The analysis of sound speed variations demonstrates that both temperature and salinity significantly impact acoustic propagation in seawater. Heatmap, 3D surfaces, and contour plots consistently show that sound speed increases with both parameters, emphasizing the importance of incorporating real-time environmental data into sonar models. These findings are crucial for accurate sonar signal processing, as variations in sound speed directly affect signal refraction, attenuation, and target localization. Machine learning-based sonar classification techniques, such as CNN and LSTM, prove to be significantly superior to traditional SONAR in accurately identifying sonar signals. The results indicate that LSTM outperforms CNN due to its ability to capture temporal dependencies, reinforcing the need for advanced deep learning techniques in sonar data interpretation. Additionally, beamforming SONAR exhibits improved directional selectivity compared to traditional SONAR, enhancing target detection accuracy by focusing acoustic energy in specific directions.

The paper also explores the trade-offs in sonar system design. A clear inverse relationship between sonar frequency and coverage area is observed, where lower frequencies provide broader coverage but lower resolution. In comparison, higher frequencies enable finer resolution at the cost of reduced range. This trade-off must be carefully considered based on the operational objectives, such as long-range surveillance versus high-resolution imaging. Similarly, the frequency vs. resolution trade-off highlights that higher frequencies improve target discrimination, making them ideal for applications requiring precise object identification. Energy consumption analysis reveals a steady decline in energy reserves as mission duration increases, underscoring the importance of efficient power management in autonomous sonar operations. The findings suggest that optimizing energy consumption strategies is critical for extending mission endurance, particularly for long-duration underwater explorations. Finally, the paper highlights the impact of depth on sonar signal attenuation, showing that attenuation increases with depth due to absorption, scattering, and pressure effects. These insights emphasize the necessity of depth-aware sonar calibration to maintain signal integrity in deep-sea environments.

This paper proposed and evaluated a bio-inspired sonar system for AUV navigation, demonstrating significant advancements over traditional SONAR systems. By integrating echolocation principles, deep learning, and multi-sensor fusion, the proposed model, SonarNet, significantly enhances underwater object detection, obstacle avoidance, and real-time decision-making. Simulation results validate SonarNet’s superior performance across multiple key aspects. Notably, SonarNet achieved a detection accuracy ranging from 90 to 94%, significantly surpassing the 82% to 87% accuracy of traditional SONAR systems, as shown in Fig. 9, highlighting its robust feature extraction and classification capabilities. Furthermore, SonarNet demonstrated a remarkable 25% reduction in energy consumption compared to traditional SONAR, extending AUV operational time from 3.8 to 5.2 h, as depicted in Fig. 10 and Fig. 11. The implementation of adaptive filtering and DSP resulted in a 2 to threefold improvement in SNR, significantly enhancing object classification accuracy, as illustrated in Figs. 15 and 17. Acoustic-based positioning via USBL outperformed GPS-based localization, achieving a threefold reduction in positional error, making it highly suitable for deep-sea navigation, as shown in Fig. 18. The CNN-LSTM hybrid model exhibited stable convergence, with validation accuracy exceeding 90% after 20 training epochs, as shown in Figs. 12 and 13. These findings collectively underscore the substantial potential of bio-inspired sonar technologies to enhance the efficiency and reliability of AUVs in diverse applications, including marine exploration, environmental monitoring, and underwater surveillance. Despite its demonstrated advantages, the proposed bio-inspired sonar system (SonarNet), while promising, presents several limitations that warrant consideration. Firstly, the proposed model’s high computational complexity poses a significant challenge for real-time processing, particularly on resource-constrained AUVs. This computational burden hinders the system’s immediate applicability in embedded systems, demanding further optimization for efficient real-time performance. Secondly, the system’s reliance on simulated sonar echoes and external datasets, while valuable, may not fully capture the intricate complexities of real-world environmental variability and sensor noise. To enhance generalization, extensive real-world data collection and augmentation are essential. Thirdly, although the system exhibits adaptability to varying oceanic conditions, factors such as turbidity, salinity, and thermal layers can still degrade performance. While Kalman filtering enhances sensor fusion, it may not entirely mitigate the adverse effects of extreme environmental distortions. Lastly, the training versus validation curves indicate a potential risk of model overfitting, as seen in Fig. 13. To address this, implementing regularization techniques such as dropout and data augmentation is crucial for improving the model’s ability to generalize to unseen data. These limitations highlight areas for future research and development to enhance the robustness and real-world applicability of the bio-inspired sonar system.

The improvements observed in this work are based on simulation tests rather than field trials, and future work will focus on validating these findings with real sonar measurements. Future research should focus on integrating real-time adaptive sonar models that adjust for environmental variations, improving deep learning models for sonar classification, and exploring advanced energy-efficient designs for extended mission capabilities. Further exploration of multi-frequency sonar systems could also optimize the balance between range, resolution, and energy consumption for enhanced underwater operations. By leveraging these findings, sonar technology can be significantly improved to support a wide range of applications, including underwater navigation, marine exploration, and AUV operations, ultimately leading to more accurate and reliable underwater sensing systems.

## Data Availability

The data sets used are available at: — 1. NOAA Oceanographic Data (Temperature, Salinity, Currents, etc.): —World Ocean Database (WOD): https://www.ncei.noaa.gov/products/world-ocean-database —Global Ocean Currents Database (GOCD): https://www.ncei.noaa.gov/access/metadata/landing-page/bin/iso?id=gov.noaa.nodc:NCEI-GOCD —Ocean Currents & Salinity: https://sos.noaa.gov/catalog/datasets/ocean-currents-salinity/ 2. NASA / NASA-JPL ECCO Reanalysis Products: —ECCO Ocean Temperature & Salinity (0.5° Monthly Mean): https://podaac.jpl.nasa.gov/dataset/ECCO_L4_TEMP_SALINITY_05DEG_MONTHLY_V4R4 —ECCO Ocean Temperature & Salinity (0.5° Daily Mean): https://podaac.jpl.nasa.gov/dataset/ECCO_L4_TEMP_SALINITY_05DEG_DAILY_V4R4 —Multi-Mission Optimally Interpolated Sea Surface Salinity (OISSS): https://podaac.jpl.nasa.gov/dataset/OISSS_L4_multimission_monthly_v2 3. FAO Data Sources: https://www.fao.org/aquastat/en/databases/ 4. Duke University’s Marine Robotics and Remote Sensing Lab Data Source: —BEANS: The Benchmark of Animal Sounds: https://github.com/earthspecies/beans —Discovery of Sound in the Sea (DOSITS): https://dosits.org/ All data used in this study are publicly available. Oceanographic variables were obtained from NOAA and NASA ECCO2 datasets, with supplementary environmental data from FAO. Bioacoustic data were sourced from publicly accessible Duke University research repositories, along with open marine sound libraries. No proprietary data were used.
